# Effectiveness of the Global Integration Method (Método de Integração Global - MIG) for improving motor and functional outcomes in children with autism spectrum disorder: a randomised controlled trial protocol

**DOI:** 10.3389/fped.2026.1804826

**Published:** 2026-04-22

**Authors:** Thalita Karla Flores Cruz, Reinaldo da Costa Paulino Netto, Fabiana Rachel Martins Costa, Elisa Braz Cota, Amanda Aparecida Alves Cunha Nascimento, Simone Rosa Barreto, Deisiane Oliveira Souto

**Affiliations:** 1 Postgraduate Program in Neuroscience, Federal University of Minas Gerais, Belo Horizonte, Minas Gerais, Brazil; 2 Institute of Neurodevelopment, Cognition and Inclusive Education (INCEI), Belo Horizonte, Minas Gerais, Brazil; 3 Postgraduate Program in Psychology, Cognition and Behavior, Federal University of Minas Gerais, Belo Horizonte, Minas Gerais, Brazil; 4 Postgraduate Program in Speech-Language Pathology, Federal University of Minas Gerais, Belo Horizonte, Minas Gerais, Brazil

**Keywords:** autism spectrum disorder, child development, neurodevelopmental disorders, motor skills, randomized controlled trial, rehabilitation

## Abstract

**Introduction:**

Motor impairments are highly prevalent in children with Autism Spectrum Disorder (ASD) and have been associated with reduced functional independence, participation, and sociocommunicative development. Despite the growing body of evidence supporting the relevance of motor functioning in ASD, motor-based interventions remain underrepresented in high-quality randomized clinical trials. The Global Integration Method (Método de Integração Global - MIG) is an intensive, interdisciplinary intervention grounded in the theories of predictive coding and embodied cognition, with emphasis on motor organization, proprioceptive stimulation, and generalization of functional skills in real-life contexts.

**Objective:**

The aim of this study is to evaluate the effectiveness of the MIG program in improving fundamental motor skills and achieving functional goals, compared with conventional physiotherapy and psychological interventions, in children with ASD. Secondary objectives are to investigate the effects of MIG on balance, sociocommunicative skills, and motor performance.

**Methods and analysis:**

This is a three-arm randomized controlled trial with concealed allocation and blinded outcome assessors. Sixty-six children with ASD, aged 6 to 12 years, classified as requiring level 1 or 2 support, will be randomized into one of three groups: (I) MIG program, (II) conventional psychological intervention, and (III) conventional motor physiotherapy. Interventions will last five weeks. Assessments will be conducted at baseline, immediately post-intervention, and three months after completion. Primary outcomes include fundamental motor skills and functional goal attainment. Secondary outcomes include measures of balance, sociocommunicative skills, and motor performance. Data will be analyzed using linear mixed-effects models, following the intention-to-treat principle.

**Ethics and dissemination:**

The protocol was approved by the Research Ethics Committee of the Faculty of Medical Sciences of Minas Gerais, Brazil (Approval No. 7,456,658). Written informed consent will be obtained from parents or legal guardians, and assent will be sought from participating children when developmentally appropriate. Study findings will be disseminated through peer-reviewed publications, conference presentations, and reports to participating families and institutions.

**Clinical Trial Registration:**

This protocol was prospectively registered in the Brazilian Registry of Clinical Trials https://ensaiosclinicos.gov.br/rg/RBR-7r6n8zd, identifier U1111-1326-2272.

## 1 Introduction

Autism Spectrum Disorder (ASD) is a neurodevelopmental condition defined by impairments in social communication and the presence of restricted and repetitive patterns of behavior (1). Although diagnostic criteria predominantly emphasize sociocommunicative aspects, a growing body of evidence indicates that motor impairments are widely observed characteristics in this population (2–6).

In one of the largest population-based studies on motor skills in ASD, Bhat found that approximately 88% of 13,528 children in the United States were at high risk for motor impairment. Despite this striking figure, only 32% of the children received physiotherapy and 13% participated in motor-based therapeutic activities, highlighting a substantial discrepancy between the prevalence of impairments and access to interventions (6). Licari et al. analyzed data from 2,084 Australian children diagnosed by six years of age and observed that motor difficulties are common yet underrecognized in clinical practice. The study found that 35.4% had clinically significant motor deficits and 43.7% showed moderately below-average performance, totaling nearly 80% with some level of motor impairment (2).

Motor impairments described in the literature include difficulties in fine and gross motor coordination, balance, motor planning, postural control, and manual dexterity—skills that are essential for autonomy and participation in school, social, and family activities (4). Evidence indicates that these deficits may emerge in the first years of life, even preceding sociocommunicative symptoms (7), and tend to persist across development, with cumulative effects on functionality and quality of life (8).

The Global Integration Method (Método de Integração Global - MIG) represents an innovative, intensive, and interdisciplinary intervention approach for children and adolescents with ASD (9). Unlike traditional approaches that fragment development into predominantly cognitive, behavioral, or linguistic components, MIG is based on the premise that motor function, proprioception, and bodily organization constitute foundational processes for the emergence of communication, social interaction, and functional participation in everyday life (9).

Operationally, MIG is organized into intensive interdisciplinary programs, with a monthly workload ranging from 60 to 80 h, distributed among different professionals according to functional priorities jointly defined with the family (9). Interventions take place in highly structured environments known as City of Tomorrow (Cidade do Amanhã), which replicate natural daily-life contexts such as home, school, supermarkets, and other community settings (10). In this way, training occurs in environments that simulate real life, promoting the immediate generalization of skills to contexts of greatest relevance to the child or adolescent and their family (11).

MIG is grounded in two major contemporary theoretical frameworks. The first is predictive coding theory, according to which the brain continuously operates by generating hypotheses about the environment and comparing them with incoming sensory information. In recent years, there has been a substantial increase in studies identifying predictive coding as one of the most robust and parsimonious explanations for typical and atypical brain functioning (12–14). In the case of ASD, there is consistent evidence that children and adolescents experience difficulties integrating sensory, motor, and social cues, which compromises their ability to form stable predictions about the environment and to update those predictions efficiently (15, 16).

The second theoretical foundation is embodied cognition, according to which complex cognitive and sociocommunicative processes do not develop independently of the body, but rather emerge in close integration with motor experiences, bodily sensations, and actions in the environment. The literature has shown that movement patterns, postural stability, coordination, rhythm, and active exploration directly influence the development of communicative and social competencies from the earliest years of life (17, 18). This perspective challenges traditional views that treat language, social interaction, and motor function as independent domains, instead suggesting that these systems are organized in a mutually dependent manner.

One of the central components of MIG, directly aligned with the principles of predictive coding and embodied cognition, is the use of the MIG Flex therapeutic suit, an exoskeleton developed based on the mapping of myofascial lines and designed to provide continuous proprioceptive stimulation, postural support, and bodily organization (9). By stabilizing the trunk and aligning body segments, the suit promotes more consistent bodily signals, thereby reducing the amount of prediction error generated by the organism. This sensorimotor stability increases the predictability of bodily experiences, helping the child better anticipate the consequences of their movements and organize actions more efficiently.

Another distinguishing feature of MIG is the systematic integration of cognitive, behavioral, and structured teaching strategies organized through narrative grammar (9). The literature consistently demonstrates the effectiveness of cognitive and behavioral techniques in enhancing adaptive skills, promoting functional communication, and developing social repertoires in children with ASD (19, 20). In parallel, narrative grammar—understood as a cognitive schema that organizes events into comprehensible structures with characters, intentions, actions, and consequences—has been shown to be a promising resource in interventions for individuals with ASD, as it provides predictability, temporal coherence, and support for processing complex social information (21).

In addition, MIG adopts a family-centered therapeutic planning model, in which goals are collaboratively defined and based on real functional needs within daily routines (10, 22). This alignment between intervention and everyday life increases the practical relevance of the program and strengthens adherence, ensuring that the skills developed have immediate utility in contexts of greatest importance to the child and their caregivers.

Despite growing evidence indicating that motor impairments are highly prevalent in ASD and influence sociocommunicative development, a significant gap remains in clinical practice, which the MIG protocol seeks to address. The effects of MIG have been investigated in children and adolescents with ASD and indicate that the intervention promotes meaningful functional improvements when assessed through parent-reported outcomes and measures sensitive to change (10, 11, 22). *Although no previous feasibility studies have been conducted, pre- and post-intervention studies indicate that children and their families are able to attend and complete most of the sessions required by this intensive program, suggesting good feasibility and adherence* (10, 11, 22)*.* Taken together, these studies suggest that MIG is a promising intervention capable of producing functional, communicative, and motor improvements, with strong family acceptance and alignment with contemporary family-centered care guidelines. However, MIG has not yet been evaluated in randomized controlled trials. Therefore, it is necessary to conduct a study that evaluates the potential effects of MIG when compared with conventional interventions.

## 2 Objectives

This study aims to evaluate the effectiveness of the MIG program for children and adolescents with ASD in improving fundamental motor skills (capacity) and in achieving functional goals, compared with conventional treatments (psychology based on cognitive and behavioral techniques and Applied Behavior Analysis, and motor physiotherapy). As a secondary objective, this study seeks to verify the effectiveness of the MIG program on balance, sociocommunicative skills, and motor skills (performance).

## 3 Methods

### 3.1 Design

This study is a three-arm, single-blind randomized controlled trial designed to compare the Global Integration Method (MIG) program with conventional physiotherapy and conventional psychological interventions. Participants will be randomly allocated to one of three groups, with concealed allocation and blinded outcome assessors. Assessments will be conducted at three time points: baseline, immediately after the intervention period, and three months after intervention completion. The trial protocol was developed in accordance with the Standard Protocol Items: Recommendations for Interventional Trials (SPIRIT) guidelines (23).

### 3.2 Patient and public involvement (PPI)

Patients and the public were not involved in the design of the study or in the definition of research questions and outcome measures. However, to ensure that the perspectives of healthcare professionals and families are integrated into the study, a monitoring committee will be established. This group will consist of a clinical specialist and a family representative (a caregiver of a child with ASD), acting in an advisory capacity during the analysis and interpretation of the results. Their contribution is expected to enrich the discussion by integrating technical expertise with practical, real-world perspectives, helping to ensure that the final conclusions are both clinically meaningful and socially relevant.

### 3.3 Setting

This study will be conducted at two distinct sites. MIG program sessions will take place at the Reabilitar Clinic, within the “Cidade do Amanhã” (City of Tomorrow) facility, located in Ribeirão das Neves, Minas Gerais, Brazil. Conventional physiotherapy and psychology sessions will be conducted in the standard treatment rooms of the Reabilitar Clinic.

### 3.4 Participants

The study sample will be recruited by convenience from public institutions, philanthropic organizations, and private physiotherapy facilities located in the municipality of Ribeirão das Neves, Minas Gerais, Brazil. Children aged 6 to 12 years at the start of the intervention, with a confirmed diagnosis of Autism Spectrum Disorder (ASD) and classified as requiring level 1 or 2 support, will be included. Participants presenting cognitive, behavioral, or clinical comorbidities that may interfere with adequate comprehension of instructions or with the safe performance of the tasks included in the proposed intervention program will be excluded.

### 3.5 Sample size

The sample size was set at 66 participants, distributed into three groups with 22 individuals in each group. Sample size determination was based on the findings of a previous investigation that analyzed the effects of the MIG program in children with ASD (10). The calculation considered an effect size of 0.87, derived from outcomes of the Canadian Occupational Performance Measure, a statistical power of 80%, a significance level of *α* = 5%, and an estimated attrition rate of 20% over time. The calculation was performed using G*Power software version 3.1 (24).

### 3.6 Randomization and blinding

**Figure 1:**
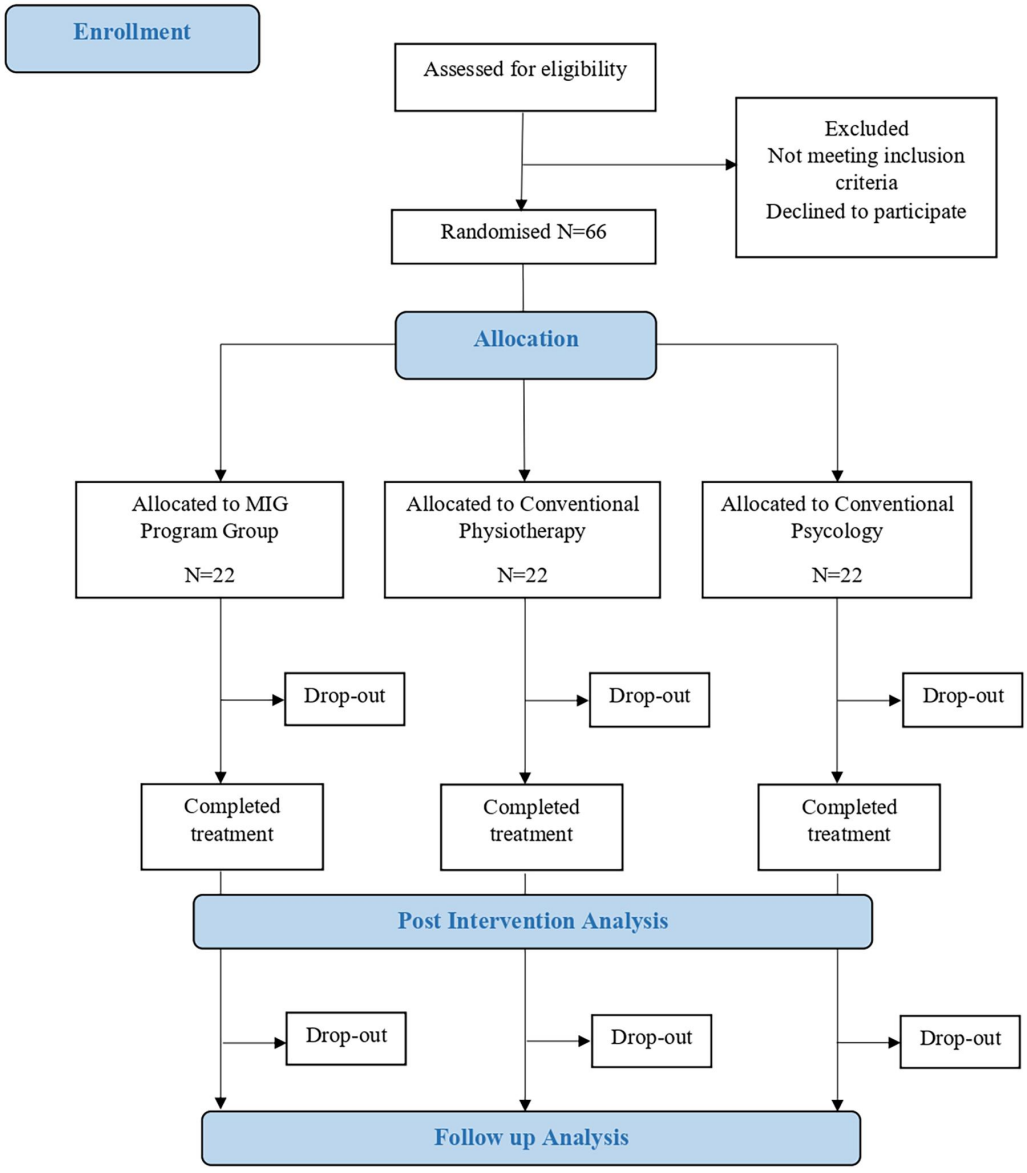
Study flowchart.

Participant recruitment and enrollment will be conducted by the principal investigator (TKFC). Allocation will follow an individual randomization scheme into three groups: (I) MIG program, (II) conventional psychology, and (III) conventional physiotherapy. The randomization sequence will be computer-generated by an independent researcher (IC) who is not involved in assessment or intervention procedures. To ensure allocation concealment, sequentially numbered, sealed, opaque envelopes will be used. Immediately prior to the first session, a designated researcher will open the corresponding envelope to determine participant allocation. All baseline assessments will be performed prior to randomization, and outcome assessors will remain blinded to group assignment. Due to the nature of the interventions, blinding of participants and therapists will not be feasible. At the end of the study, the effectiveness of blinding will be assessed by asking evaluators about their perceptions regarding participant allocation. The participant flow process is presented in Figure 1.

### 3.7 Interventions

#### 3.7.1 MIG program

Participants in this group will receive a protocol specifically structured to assess the motor component of the MIG program. The intervention will be delivered by physiotherapists with prior experience in MIG and additional training provided by an interdisciplinary team (psychologists, speech-language pathologists, and occupational therapists), ensuring standardization of the research protocol implementation. The motor component will be delivered in three weekly sessions, each lasting 50 min, over a period of five weeks, following a previously defined structure. *Although the MIG is conceived as an intensive interdisciplinary program, the present trial was designed to isolate and evaluate only its motor component; therefore, the dosage reported here refers exclusively to the physical therapy sessions and does not correspond to the total interdisciplinary workload of the complete MIG protocol.*

In all sessions of the motor component protocol of the MIG program, the child will wear the MIG Flex therapeutic suit. This suit provides proprioceptive input and postural support during motor activities. This resource serves a dual function by promoting bodily stability and improved motor organization, while reducing the cognitive load required for postural maintenance and movement coordination, thereby freeing attentional and cognitive resources that can be directed toward interaction, play, and communication. At the beginning of each session, the physiotherapist will place and adjust the MIG Flex on the child and then initiate the narrative designed for that session. The narrative will introduce the main character, the setting, and the temporal context (initial situation), as well as the problem, triggering event, or central challenge (initiating event). The character's feelings or emotions and the plan for problem resolution will also be explicitly presented; this plan will correspond directly to the motor activities the child must perform to “help” the character, together with psychoeducational components about emotions aimed at promoting generalization of concepts and strengthening cognitive flexibility by allowing the participant to recognize common principles across different symbolic situations. The narratives used in sessions 01 and 15 are described in Supplementary Material 1.

The entire protocol structure will be contextualized and guided by narrative grammar, which will organize the session stages: (I) movement preparation with active myofascial mobilization; (II) core strengthening; (III) motor skills circuit, including balance, coordination, strength, motor planning, and power; and (IV) contextualized target activity. For these stages, various pieces of motor circuit equipment will be used, such as cones, balls, and unstable surfaces. The program will include functional training of motor skills, involving multiple physical capacities (strength, endurance, balance, flexibility, agility, power, and conditioning) trained in an integrated manner through activities such as running, jumping, hopping, galloping, throwing, and kicking. Table 1 presents a technical description of the activities, in which the playful and narrative components will be used as engagement and task-contextualization strategies, while maintaining the structural elements necessary for replication of the intervention. Session delivery will be based on narrative grammar and psychoeducational components related to emotions, integrated into motor activities as a means of promoting greater engagement and meaning for the child. Task progression will follow a gradual increase in complexity, always accompanied by positive feedback and continuous encouragement. At the end of each session, the child will receive a home-based task to reinforce and consolidate the narrative elements addressed that day. An example of this material completed by the child is provided in Supplementary Material 2.

**Table 1:** MIG program motor component protocol.

Week	Preparation of the movement		Skill				Target movement		
	Myofascial tracks (spiral line and functional line)	Core	Activity 01	Activity 02	Activity 03	Activity 04	Activity 05	Activity 06	Activity 07
			Balance/proprioception	Dynamic balance	Motor coordination/ Strength	MMSS	Motor planning	Power	Target activity
1	Functional reach with trunk rotation in bipedal standing	Trunk flexion (upper abdominal crunch) with anterior reach and target between the lower limbs.	Functional reach with ring removal in bipedal support.	Tandem gait in a straight line	Squat	Bilateral ball throw with return by the therapist.	Throwing and catching toward a vertical target	Stationary running on a trampoline	Running and jumping
2	Functional reach with trunk rotation in bipedal standing	Trunk flexion with rotation associated with a functional task.	Anterior functional reach in unipodal standing	Functional gait with cone transposition	Functional squat with displacement and alternating object collection using the feet.	Bilateral transfer of objects in a horizontal plane at eye level.	Throwing and catching a vertical target with lateral movement.	Lower limb power training with bipedal jumps	Baseball
3	Functional reach with trunk rotation in bipedal standing	Hip flexion in supine position with functional task of picking up with the feet.	Static balance training in bipodal support on unstable surfaces.	Zigzag gait with visual guidance on the ground.	Lunge	Resisted load traction in a horizontal plane with lateral transfer.	Jumping over obstacles with object transfer	Sequential bipodal jumps with changes of direction (front and side)	Sequential unipodal jumps guided by visual stimulus (path by color)
4	Functional reach above the head with object grasping in bipedal standing position.	Core stabilization in supine position with limb dissociation (Dead Bug pattern)	Static balance training in unipodal stance on an unstable surface.	Lateral gait training with obstacle crossing	Alternating stationary Lunge	Low-level locomotion (crawling) with obstacle crossing and object transfer.	Step-up and step-down with alternating support.	Burpee	Lateral displacement
5	Functional reach with trunk rotation in bipedal standing	Ventral plank with elbows and knees supported.	Stationary running with transition to unipodal stance on command.	Sequential unipodal jumps to discrete floor targets	Advancement with anteroposterior displacement	Locomotion using manual support with elevation of the lower limbs (wheelbarrow standard)	Passing and receiving the ball with the feet in therapist-participant interaction.	Rhythmic bipedal rope jumping	Goal kicking

MMSS, upper limbs.

The motor component protocol of the MIG program is grounded in the systematic integration of cognitive and behavioral strategies that constitute intrinsic elements of the method and are aligned with the principles of predictive coding and embodied cognition. These strategies aim to reduce sensorimotor unpredictability, facilitate action organization, and support the construction of more stable internal models during interaction with the environment. Operationally, MIG incorporates resources widely recommended in the literature for optimizing motor learning in children with ASD (9), including the use of visual supports, modeling, prompting, task modification, immediate and positive feedback, adjusted instructions, and varied practice schedules. The structured application of these elements seeks to promote greater engagement, facilitate retention, and enhance the generalization of motor and functional skills to meaningful everyday contexts.

#### 3.7.2 Conventional psychology

Participants in this group will receive intervention delivered by psychology professionals, based on cognitive and behavioral techniques and Applied Behavior Analysis, characterizing conventional psychological treatment. The intervention will be delivered over five weeks, with 50-minute sessions twice per week, totaling 10 sessions. The protocol will focus on the development of emotional regulation skills, including recognition of basic emotions (happiness, sadness, fear, disgust, anger, and anxiety) and their expressions, identification of emotions in oneself and in others, as well as strategies for appropriate emotional expression and regulation. Sessions will follow a structured therapeutic script, with clinical flexibility according to individual needs, without direct approaches to motor training or motor functional goal attainment. Interventions will be conducted in standard treatment rooms at the Reabilitar Clinic, in Ribeirão das Neves, Minas Gerais, Brazil.

#### 3.7.3 Conventional physiotherapy

Participants in this group will receive conventional neurofunctional physiotherapy intervention, characterizing the usual motor treatment available in clinical services. The intervention will be delivered over five weeks, with two weekly sessions lasting 50 min each. Sessions will follow a minimally standardized structure consisting of warm-up (5 min), motor skills training (20 min), balance training (10 min), ball-based activities (10 min), and cool-down (5 min), with clinical flexibility for adjustments according to individual participant needs. The intervention will not include the use of therapeutic suits, narrative structures, cognitive strategies, or systematically integrated interdisciplinary components. Sessions will be conducted in standard treatment rooms at the Reabilitar Clinic, in Ribeirão das Neves, Minas Gerais, Brazil.

### 3.8 Outcome measures

#### 3.8.1 Primary outcomes

The Test of Gross Motor Development–Second Edition (TGMD-2) will be used to assess fundamental motor skills (25, 26). This standardized instrument quantitatively and qualitatively evaluates 12 motor skills, subdivided into locomotor and object control skills. Scores are derived from the sum of raw scores converted into motor quotients, allowing for normative performance analysis. Its validity and reliability for the Brazilian population with typical development were established by Valentini et al. (27), with subsequent application in studies involving children with ASD (28).

The Canadian Occupational Performance Measure (COPM) will be used to assess functional goal attainment (29). This semi-structured interview allows parents to identify, describe, and prioritize treatment goals relevant to their child's occupational performance. Each goal is rated on a 10-point scale for importance, performance, and satisfaction. The instrument demonstrates validity, reliability, and sensitivity to clinical change (29, 30).

#### 3.8.2 Secondary outcomes

The Pediatric Balance Scale (PBS) will be used to assess functional balance. The PBS consists of 14 items that evaluate progressively more complex tasks, simulating fundamental activities of daily living and challenges to static and dynamic balance. Each item is scored from 0 to 4, with total scores ranging from 0 to 56, where higher scores indicate better functional balance performance. Its psychometric properties have been validated for the Brazilian population, demonstrating adequate responsiveness (31, 32).

The ABFW Child Language Test will be used to assess functional communicative skills (33, 34). Based on systematic observation of spontaneous interaction, it allows quantitative and qualitative analysis of communicative acts, communicative means, and communicative functions, demonstrating sensitivity for assessing pragmatic language performance (33–35).

The Behavioral Observation Protocol (PROC) will assess communicative and cognitive skills through a structured play-based interaction situation (36). This instrument allows analysis of language development and symbolic play and is particularly useful for characterizing cognitive-communicative functioning patterns in children with developmental disorders (37).

The motor subscale of the Vineland Adaptive Behavior Scales (VABS) will be used to assess gross and fine motor skills through a parent-reported questionnaire (38). The VABS-II version presents adequate psychometric properties, with concurrent validity of 0.77 when compared with the Peabody Developmental Motor Scales (39).

The Developmental Coordination Disorder Questionnaire (DCDQ) will be used as a parent-reported screening tool for motor coordination difficulties in daily activities (40). The DCDQ consists of 15 items rated on a 5-point Likert scale and provides age-adjusted cut-off scores to identify children at risk for developmental coordination disorder. The instrument has demonstrated good validity and reliability and has been culturally adapted and validated for use in Brazil.

### 3.9 Procedures

Assessments will be administered by four independent and blinded assessors (a physiotherapist, a psychologist, a speech-language pathologist, and an occupational therapist), according to the specific technical expertise required for each instrument. The PBS and TGMD-2 will be administered by the physiotherapist; the COPM and the DCDQ by the occupational therapist through parent interviews; the VABS by the psychologist through parent interviews; and the PROC and ABFW by the speech-language pathologist. Assessments will be conducted at three time points: baseline, immediately post-intervention, and three months after the intervention. Baseline assessments will be completed prior to randomization. Recruitment is expected to begin in September 2025 and continue until January 2026.

### 3.10 Data analysis

Analyses will be performed using IBM SPSS Statistics software. Descriptive analyses will initially be used to characterize the sample in terms of age, sex, and level of support. Data distribution will be assessed using the Shapiro–Wilk test. Baseline group comparisons will be conducted using one-way analysis of variance (ANOVA) or Kruskal–Wallis tests, as appropriate. All analyses will follow the intention-to-treat principle. Linear mixed-effects models will be used to evaluate intervention effects over time, including group-by-time interaction terms. In the presence of significant interactions, *post hoc* analyses with Bonferroni correction will be performed. Missing data will be handled using maximum likelihood estimation within the mixed-effects models. A two-sided significance level of 5% (*p* < 0.05) will be adopted for all analyses.

## 4 Ethics and dissemination

Ethical approval was obtained from the Research Ethics Committee of the Faculty of Medical Sciences of Minas Gerais, Brazil (Approval No. 7,456,658). The trial was prospectively registered in the Brazilian Registry of Clinical Trials (RBR-7r6n8zd) and the WHO Universal Trial Number system (UTN: U1111-1326-2272). Written informed consent will be obtained from parents or legal guardians, and assent will be sought from participating children when developmentally appropriate, prior to any study procedures. All procedures will comply with national and international ethical standards for research involving human participants. Adverse events will be monitored and recorded throughout the intervention period, and appropriate clinical referral will be provided if necessary. Results will be disseminated through peer-reviewed publications, scientific conferences, and reports to participating families and partner institutions.

## 5 Discussion

This RCT protocol describes a rigorous evaluation of MIG compared with conventional physiotherapy and psychology interventions for children with ASD. Grounded in contemporary theoretical frameworks of predictive coding and embodied cognition, MIG integrates intensive motor training, proprioceptive stimulation through a therapeutic suit, and structured narrative-based activities conducted in ecologically valid environments. By targeting fundamental motor skills and functional goal attainment as primary outcomes, this study addresses a critical gap in ASD research related to the scarcity of high-quality trials investigating interdisciplinary interventions centered on the motor component. If proven effective, the findings may support a paradigm shift by recognizing motor functioning as a foundational domain influencing broader developmental outcomes in ASD. In addition, this trial may inform clinical decision-making, contribute to evidence-based service planning, and offer a replicable model for integrating motor, cognitive, and contextual approaches within a family-centered care framework.
