# Supplementary material for: Effectiveness of the Global Integration Method (Método de Integração Global - MIG) for improving motor and functional outcomes in children with autism spectrum disorder: a randomised controlled trial protocol
Source: Front Pediatr. 2026 Apr 22;14:1804826. doi: 10.3389/fped.2026.1804826 (PMC13144026; doi:10.3389/fped.2026.1804826)
Supplement: Supplementary file 1 [file Datasheet1.pdf]

Livro de Aventuras

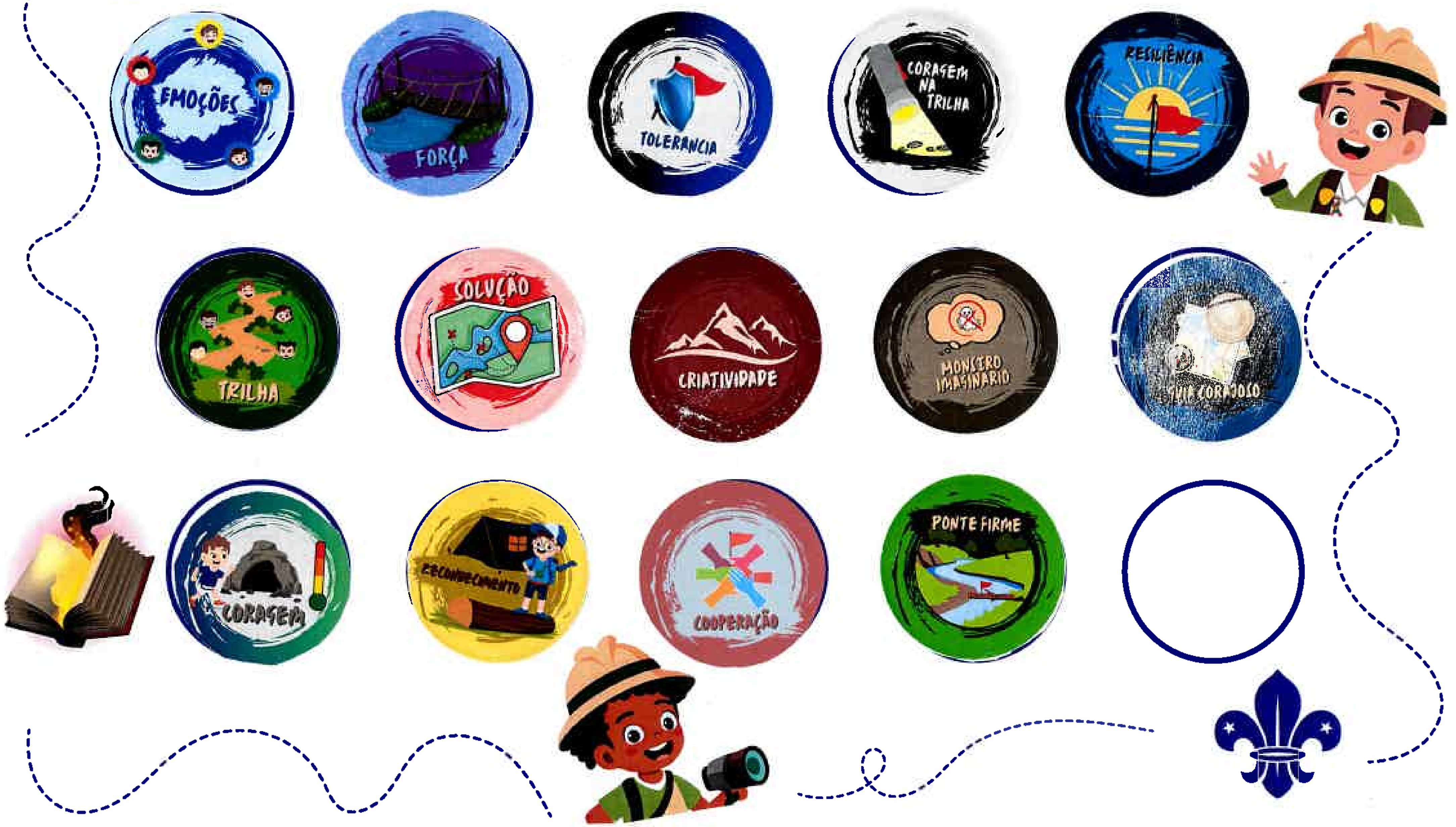

TAREFA DE CASA

No seu Livro de Aventuras, pense em uma situação em que você errou, caiu ou se machucou e teve vontade de desistir, como João, que tropeçou no pneu e sentiu raiva, tristeza e vergonha.

Agora responda:

- ***O que você estava tentando fazer?***
- ***Quais emoções apareceram (raiva, tristeza, vergonha, outra)?***
- ***Se você usasse um "Termômetro de Emoções", qual número daria para cada sentimento?***
- ***O que te ajudou a continuar ou voltar a tentar?***

Depois, crie uma nova página chamada:
"Meu plano para quando eu cair ou sentir vontade de desistir"
Nessa parte do seu livro, desenhe ou escreva duas coisas que você pode fazer nessas situações, como pedir ajuda, respirar fundo, fazer um curativo, torcer pelos outros, ou tentar de novo quando tiver chance.

Dica para os responsáveis:
Ajude a criança a usar a ideia do "Termômetro de Emoções" para reconhecer a intensidade de cada sentimento. Mostre que sentir raiva, tristeza ou vergonha não significa fracasso e que retomar, apoiar o grupo ou tentar de novo também são formas de coragem. Incentive que ela registre estratégias concretas que já funcionaram para se recuperar em momentos difíceis.

TAREFA DE CASA:

No seu Livro de Aventuras, escolha um pensamento triste ou ruim que apareceu na sua cabeça (pode ser aquele que você desenhou na tarefa anterior).

Agora responda: ***Esse pensamento é totalmente verdadeiro? Existe outra maneira de pensar sobre isso?***

Escreva ou desenhe uma nova versão desse pensamento, mais leve ou mais justa com você.

Você também pode imaginar um "pensamento amigo", ou seja, algo que você diria para um amigo que estivesse passando pela mesma situação.

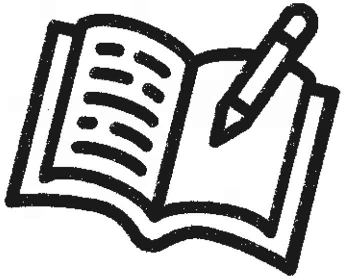

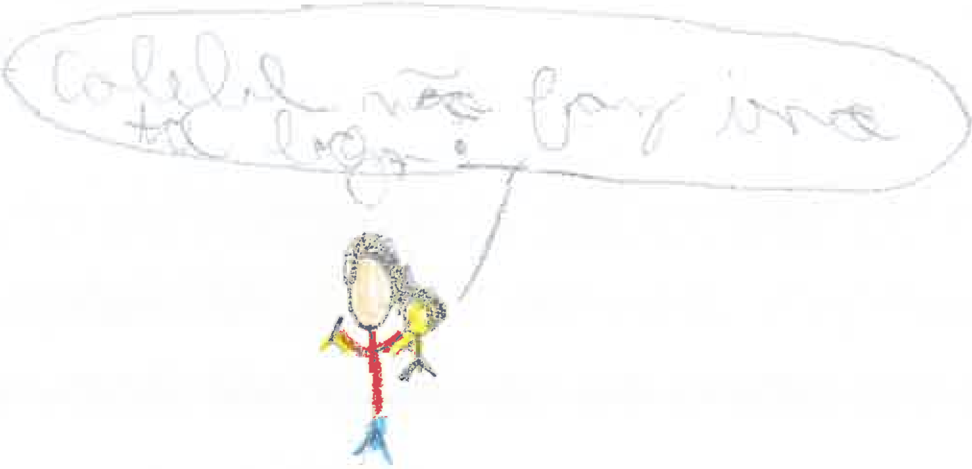

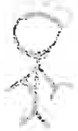

TAREFA DE CASA:

Desenhe no Livro de Aventuras situações do dia a dia em que você sente emoções fáceis ou difíceis. Explique o porquê elas são assim para você. Recorde-se de cada uma das emoções:

- *Alegria*
- *Tristeza*
- *Raiva*
- *Medo*
- *Nojo*

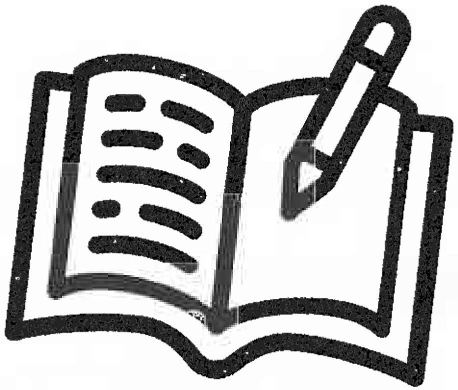

Para mim o nojo é uma sensação de explã-
vea.

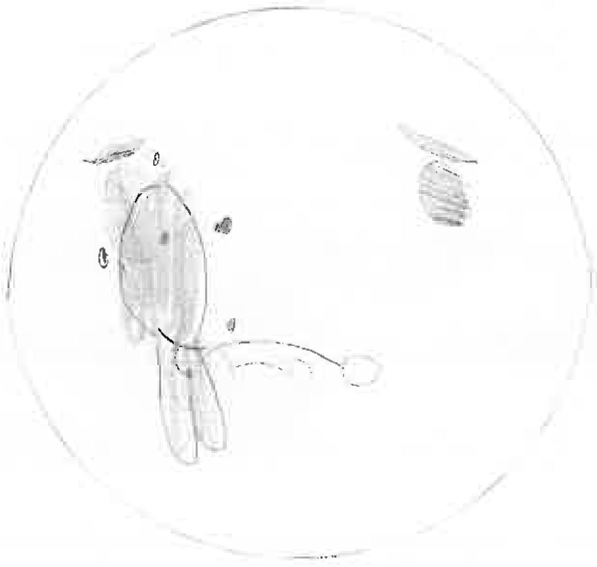

O medo para mim é como se fosse um filme de terror.

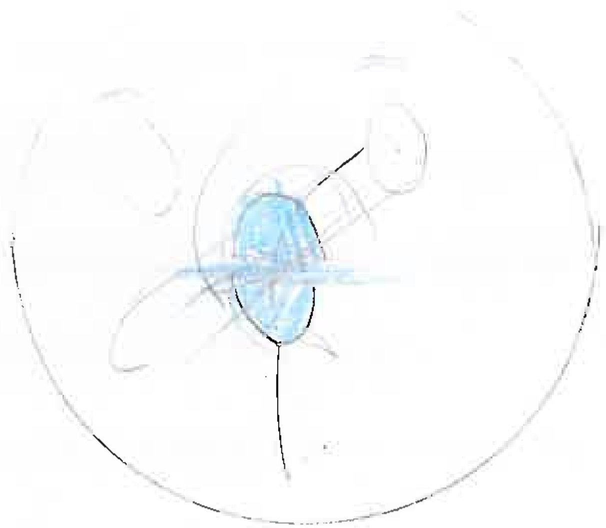

A tristeza para mim é um filme triste.

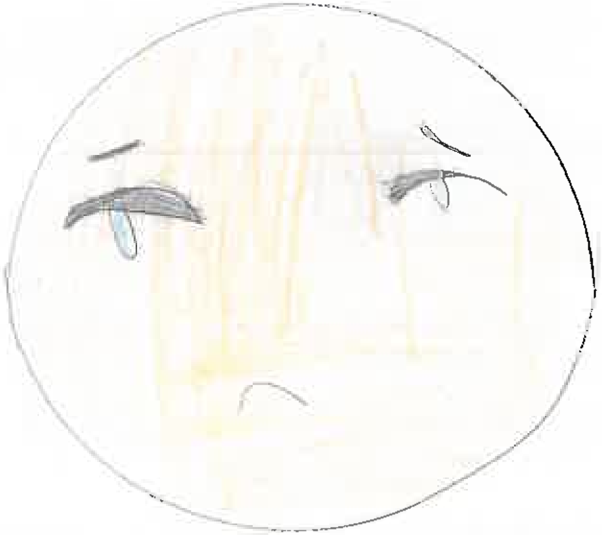

(Peça desmontada)

A alegria para mim é um parque de diversão.

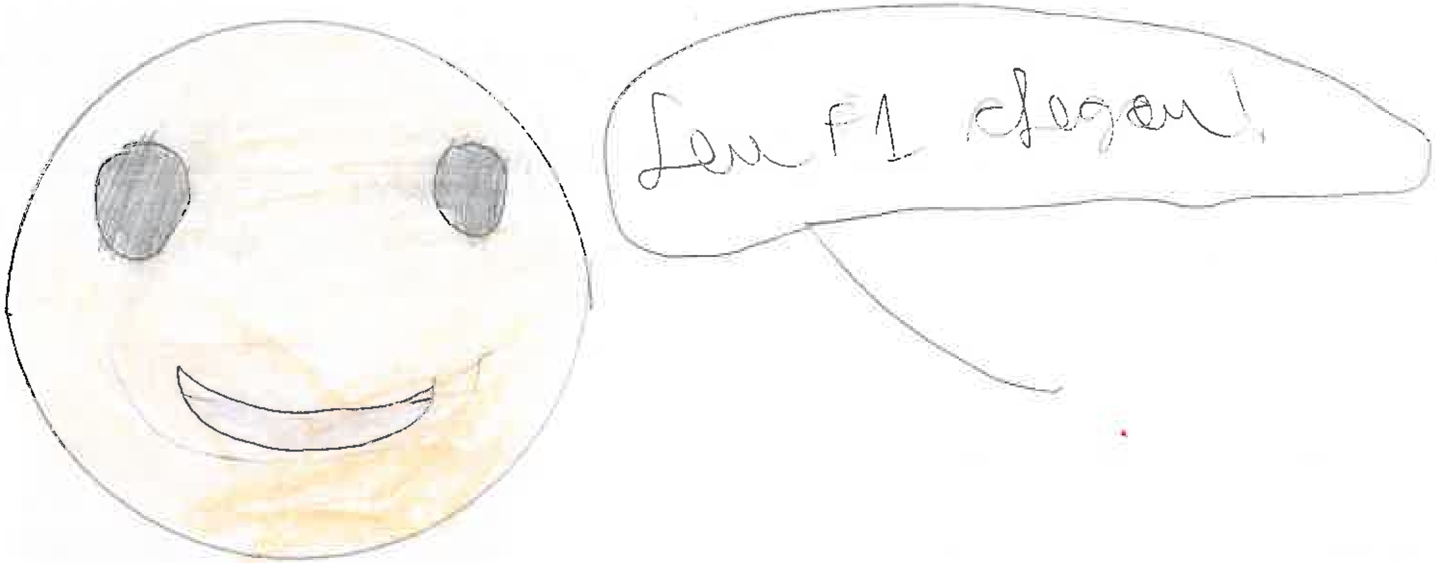

A Raiva para mim é um desafio completo.

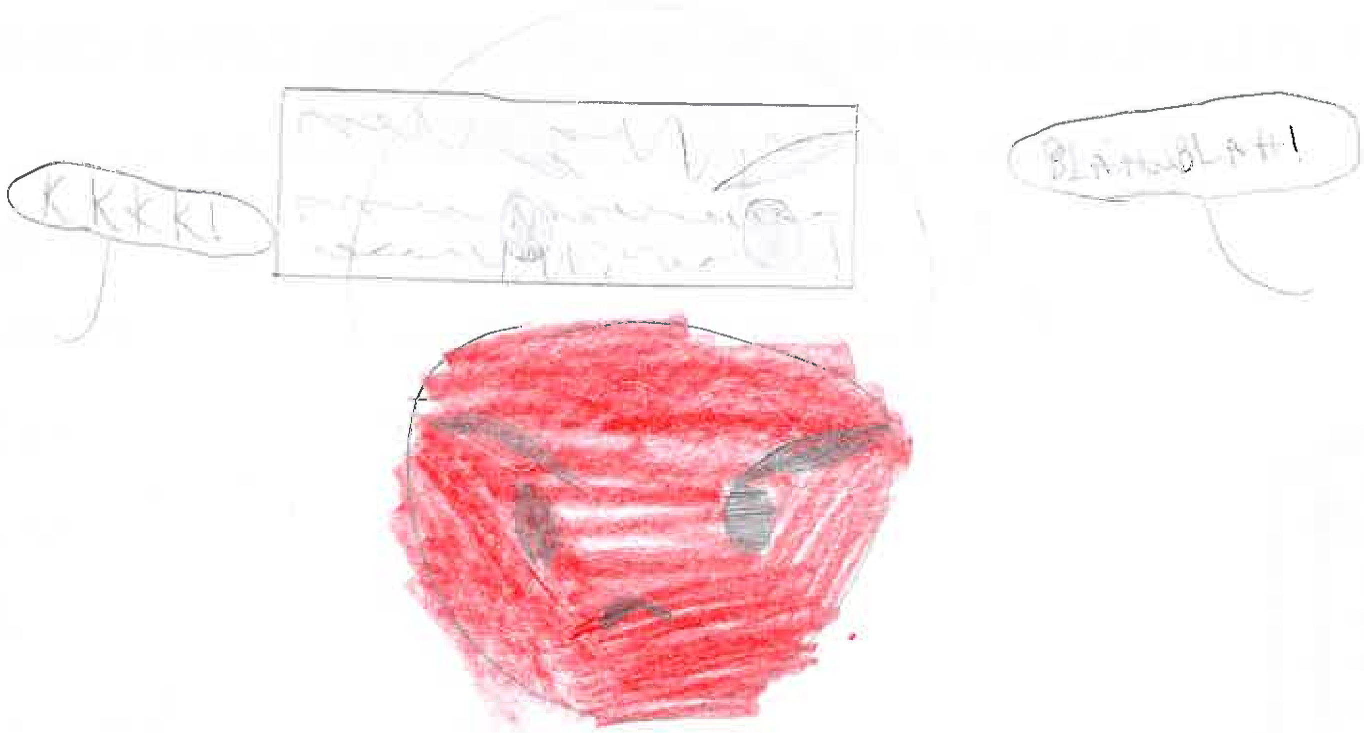

TAREFA DE CASA:

Desenhe no Livro de Aventuras situações em que você sinta cada uma das emoções e o nível (de 0 a 5):

- *Alegria*
- *Tristeza*
- *Raiva*
- *Medo*
- *Nojo*
- *Surpresa*

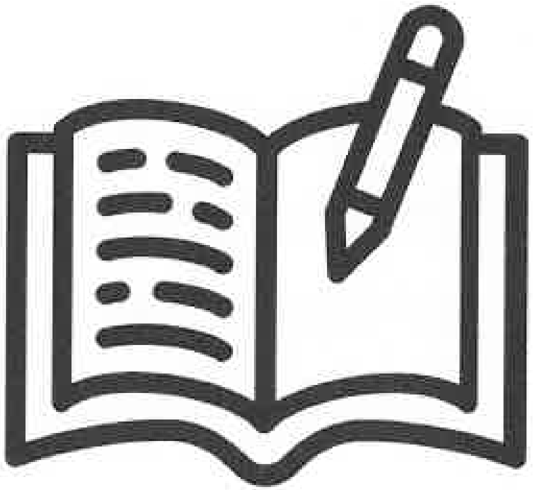

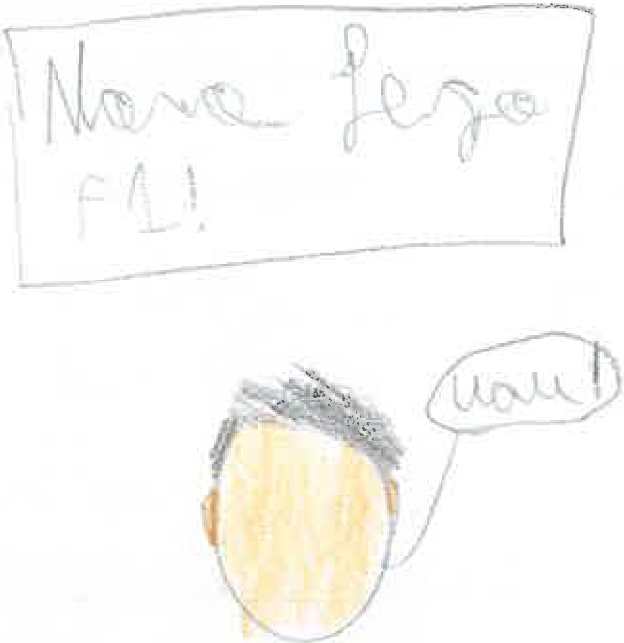
Nova Lego
F1!
uau!

(Alguién peida en sala)

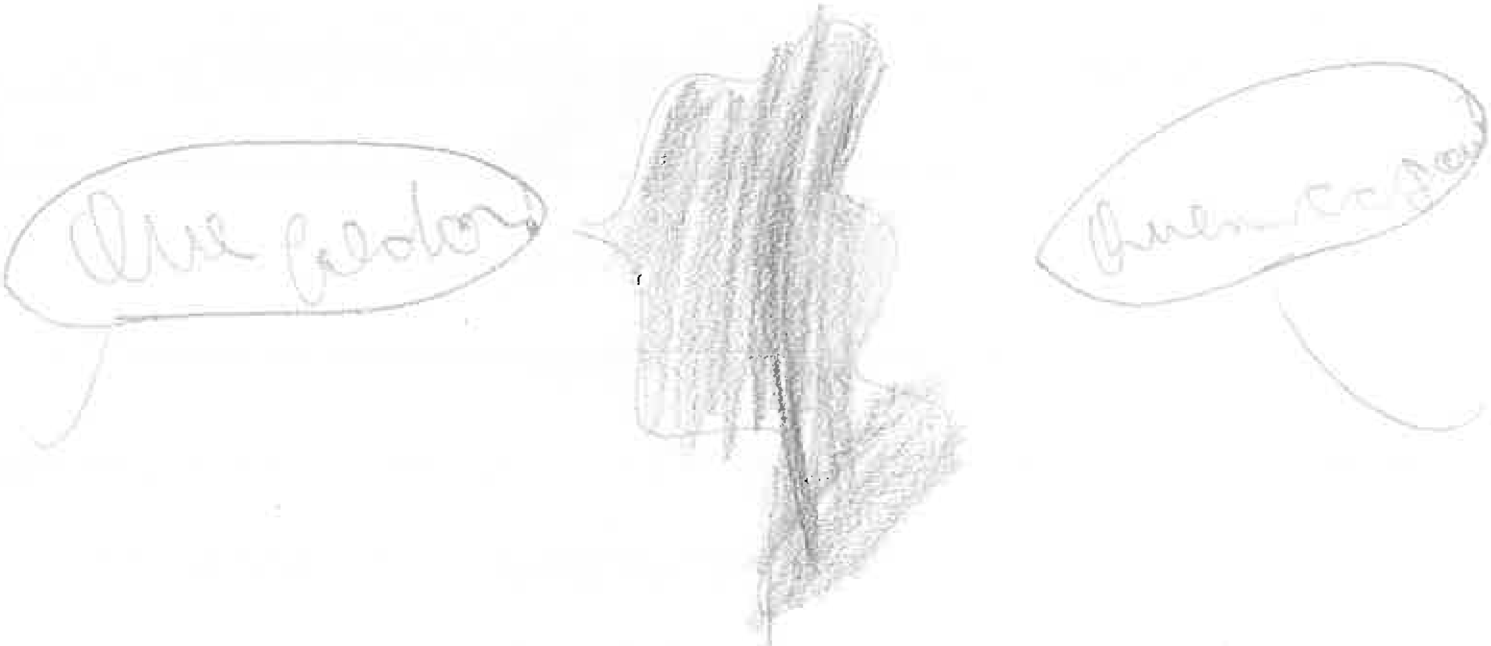

nivel: 2

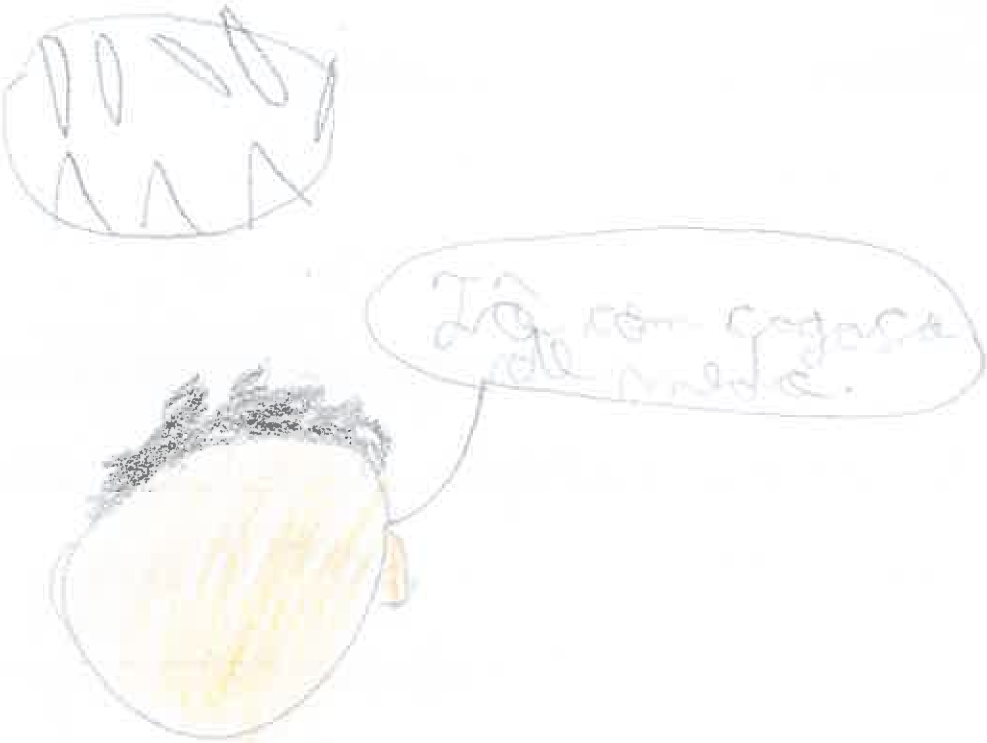

Nível: 4

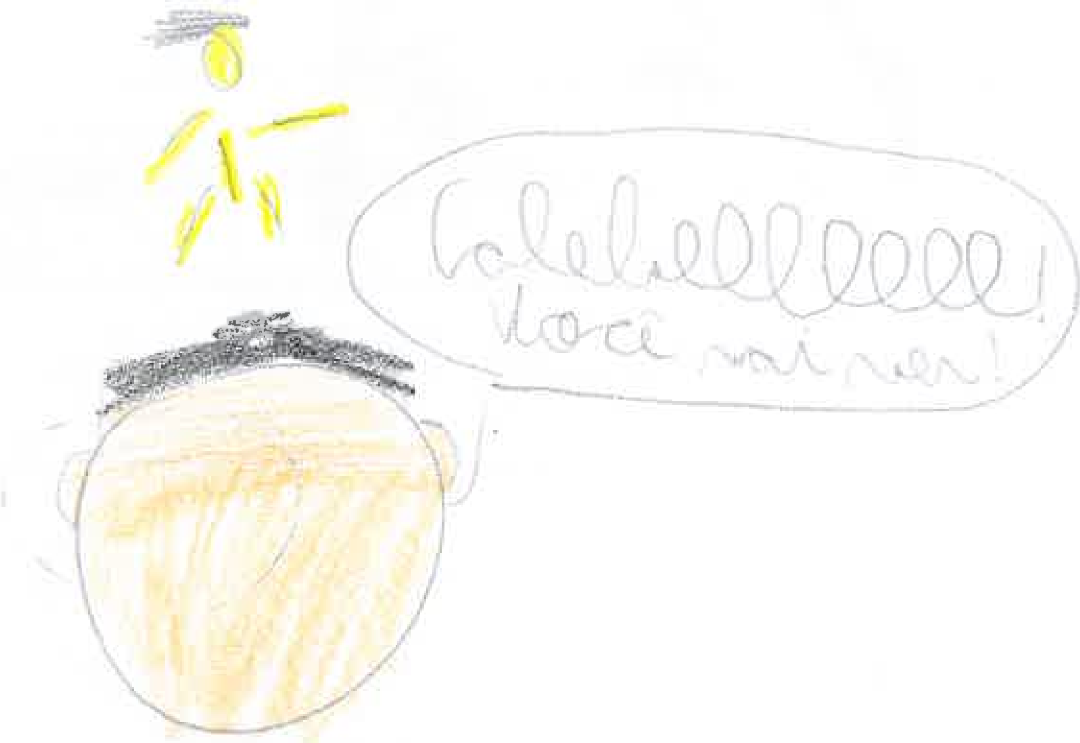

nível : 3

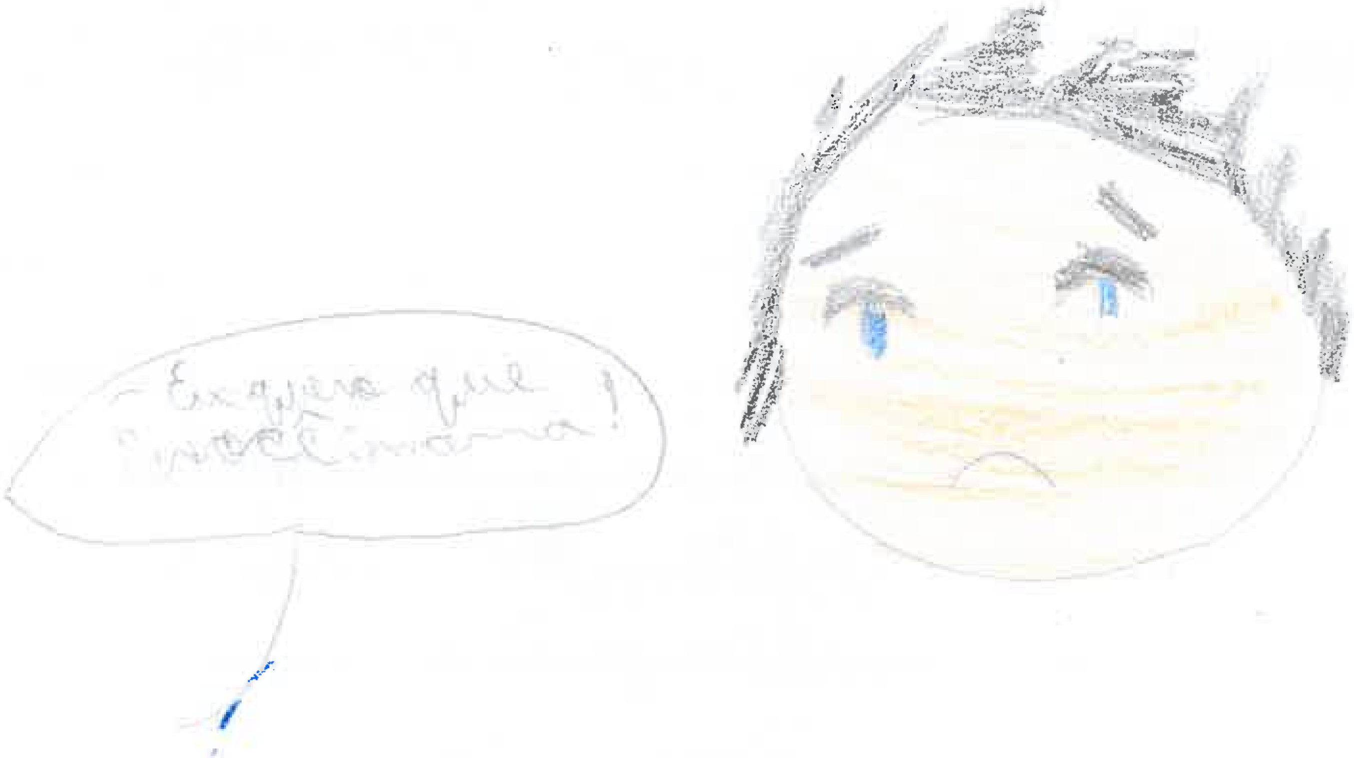

nível: 5

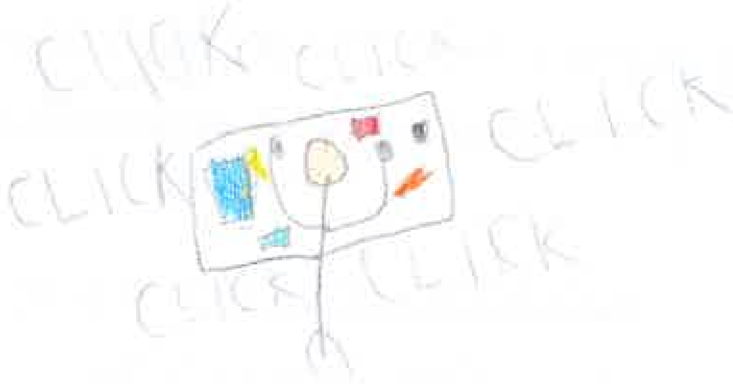

nível: 4

TAREFA DE CASA:

No seu Livro de Aventuras, desenhe uma situação em que você ficou com raiva durante essa semana. Depois, marque o nível da raiva que sentiu (de 0 a 5, sendo 0 "bem fraquinho" e 5 "muito forte").

Vamos aprender a respirar para acalmar:

1. *Cheire a flor pelo nariz por 4 segundos.*
2. *Sopre a flor pela boca bem devagar por 6 segundos.*

Dica para os responsáveis:
Pratique junto com a criança usando uma flor de verdade, um desenho ou até mesmo um brinquedo. Faça a contagem com voz calma e lenta.

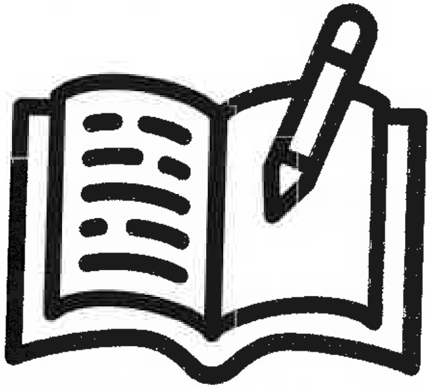

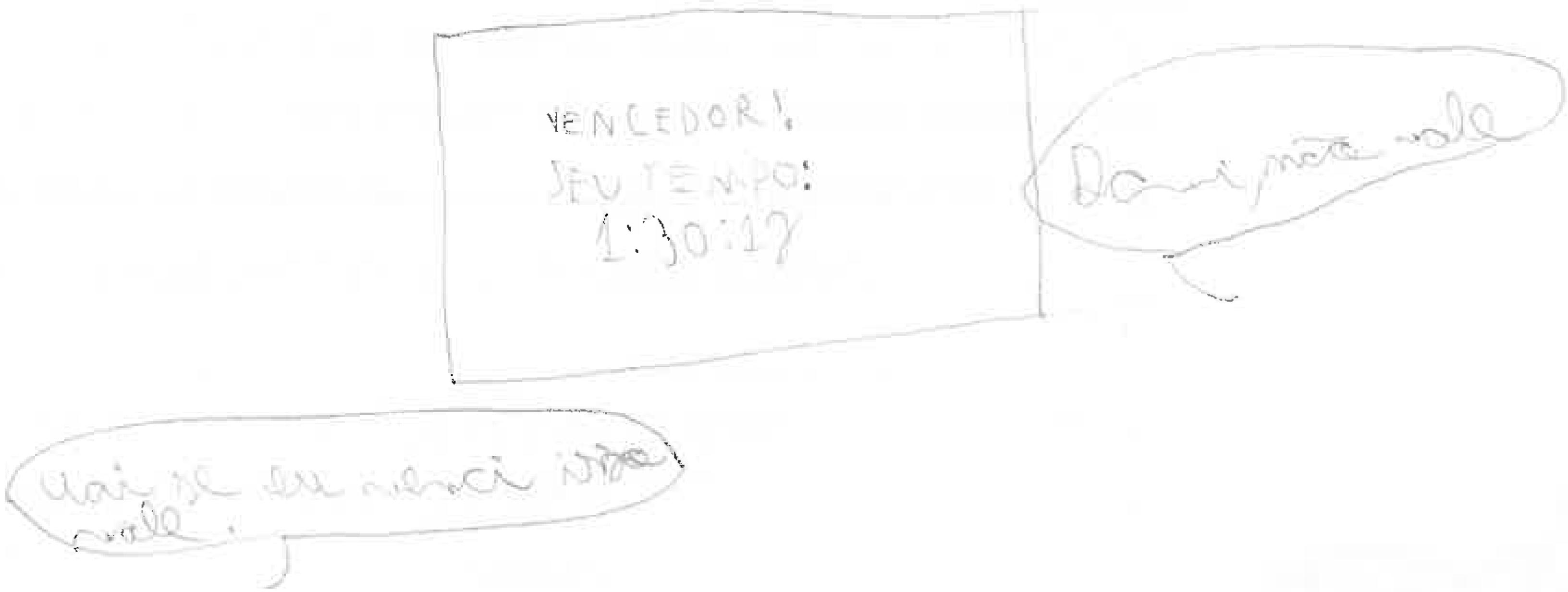

Nível: 7

TAREFA DE CASA:

No seu Livro de Aventuras, desenhe uma situação em que você ficou com raiva durante essa semana. Depois, marque o nível da raiva que sentiu (de 0 a 5, sendo 0 "bem fraquinho" e 5 "muito forte").

Respire fundo 10 vezes. Explique o que aconteceu e como você se sentiu sem gritar.

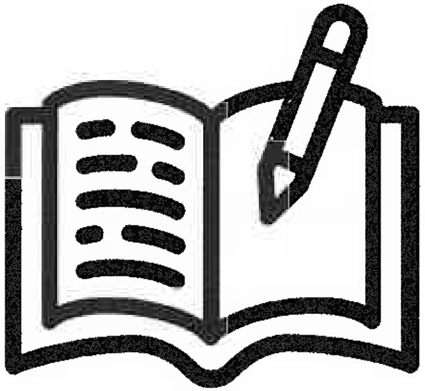

Dica para os responsáveis:
Ajude a criança falar sobre o que aconteceu e o que ela sentiu em situações estressantes. Estimule-a a respirar fundo algumas vezes para se acalmar. Então, pergunte o que aconteceu e elogie sempre que ela explicar com calma.

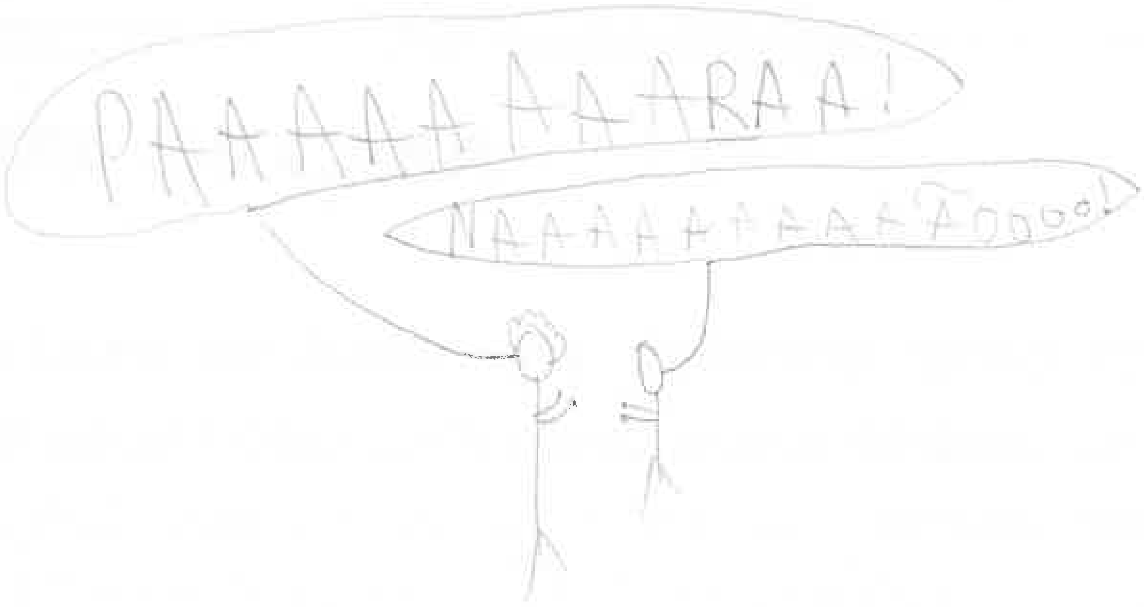

[illegible] :S

TAREFA DE CASA:

No seu Livro de Aventuras, desenhe uma situação em que você ficou com raiva durante essa semana. Depois, marque o nível da raiva que sentiu (de 0 a 5, sendo 0 "bem fraquinho" e 5 "muito forte").

Respire fundo 10 vezes. Pense como você poderia ter resolvido o problema depois de se acalmar.

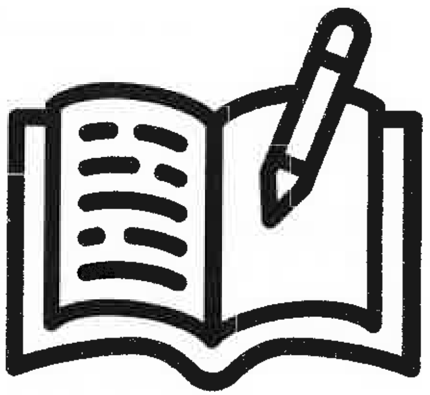

Dica para os responsáveis:
Ajude a criança a pensar em diferentes formas de resolver o problema. Deixe que ela levante diferente opções e pergunte as vantagens e desvantagens de cada uma.

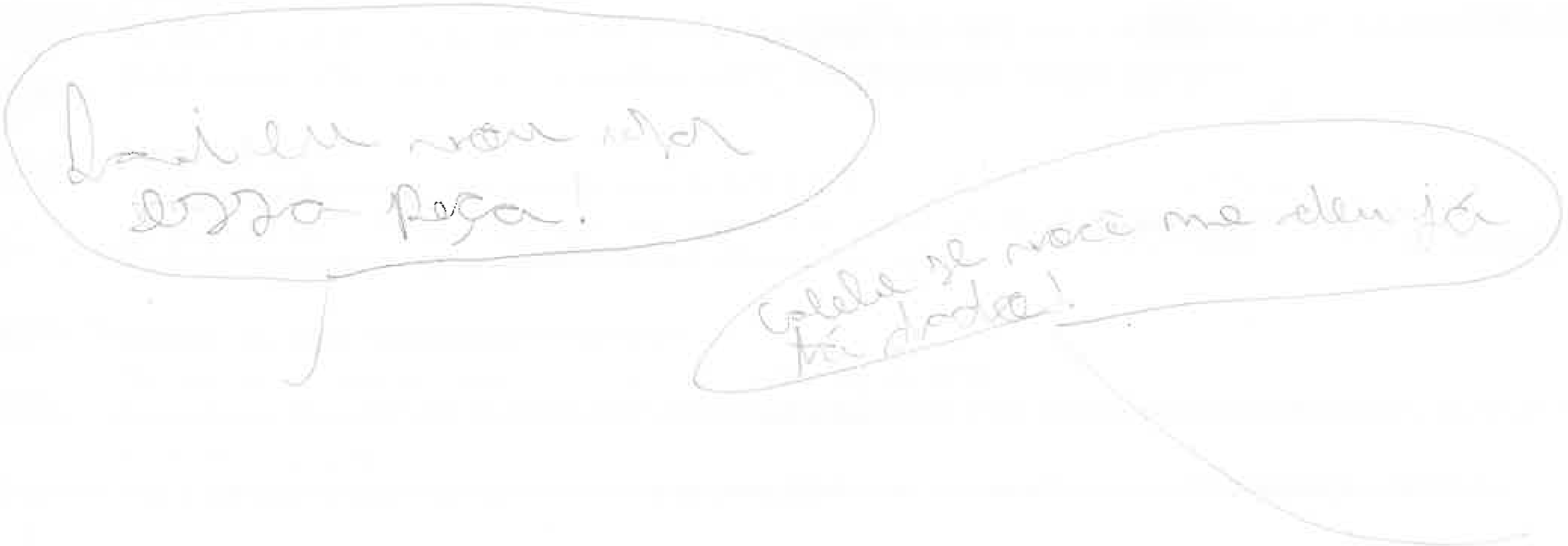

nível: 3

TAREFA DE CASA:

No seu Livro de Aventuras, pense em uma situação em que você teve medo de alguma coisa que ainda não tinha acontecido — como se sua cabeça tivesse inventado uma história assustadora.

Agora responda:

1. *O que você pensou que poderia acontecer?* Não sei.
2. *Você viu ou ouviu algo real que confirmasse esse medo? Ou foi só um pensamento?* Escutei, não sei.
3. *O que aconteceu de verdade no final?* Nada.

Depois, crie uma nova página chamada:
"Pensamentos que assustam — e o que aconteceu de verdade"
Escreva ou desenhe um pensamento que te assustou e, ao lado, o que realmente aconteceu quando você enfrentou a situação.
Você também pode usar cores diferentes: uma para o pensamento e outra para o que era verdade.

Dica para os responsáveis:
Ajude a criança a perceber a diferença entre pensamentos e fatos. Incentive que ela lembre de momentos em que se sentiu segura depois de enfrentar algo que parecia muito assustador. Reforce que os pensamentos nem sempre dizem a verdade — e que podemos aprender a verificar com calma o que é real.

TAREFA DE CASA

No seu Livro de Aventuras, pense em uma situação em que você errou alguma coisa na frente de outras pessoas — como João, que deixou a corda cair no riacho e sentiu vergonha.

Agora responda:

- ***O que você tinha que fazer?***
- ***Que pensamentos apareceram na sua cabeça?***
- ***O que você sentiu no corpo naquele momento?***
- ***Você conseguiu continuar depois do erro? O que te ajudou?***

*Depois, crie uma nova página chamada: **"Meu plano para quando eu errar e sentir vergonha"** Nessa parte do seu livro, desenhe ou escreva duas coisas que você pode fazer quando sentir vergonha por ter errado, como pedir desculpas, tentar de novo, pedir ajuda, respirar fundo ou pensar em outra forma de resolver.*

Dica para os responsáveis:
Ajude a criança a reconhecer que sentir vergonha ou tristeza diante de um erro é natural, mas que essas emoções não precisam impedir a continuidade. Incentive-a a nomear os pensamentos que surgiram na hora e a registrar estratégias que já funcionaram em situações parecidas, reforçando que pedir desculpas, corrigir o erro e recomeçar são atitudes valiosas para aprender e conviver em grupo.

Plano de coragem

1º um Lego de F1

1º Montar Lego

A situação do meu Lego (17)
F1

TAREFA DE CASA:

No seu Livro de Aventuras, desenhe ou escreva sobre uma situação em que você sentiu ansiedade ou ficou muito preocupado com algo que ainda nem tinha acontecido (como João na trilha escura).

Depois, responda:

1. *Que pensamentos passaram pela sua cabeça naquele momento?* Assalto
2. *O que você sentiu no seu corpo? (Ex: coração acelerado, pernas tremendo, barriga apertada?)* Nada
3. *Você conseguiu se acalmar? O que te ajudou?* Eu consegui nada
4. *O que você poderia tentar da próxima vez para se sentir mais seguro?* [illegible] my Lego

Agora, crie uma página especial no seu livro chamada:
"Plano de Coragem"
Ali, desenhe ou escreva duas ideias que te ajudam a enfrentar a ansiedade sem fugir. Pode ser algo que você diga para si mesmo, algo que te acalme, ou uma atitude corajosa que já teve antes.

Dica para os responsáveis:
Ajude a criança a diferenciar medo real de preocupação imaginada (ansiedade). Incentive que ela nomeie o que ajuda a se sentir segura e valide pequenos atos de coragem, como continuar mesmo ansiosa. Reforce que coragem não é a ausência do medo, mas a decisão de seguir em frente apesar dele, assim como João fez na trilha.
