# Supplementary material for: Effectiveness of the Global Integration Method (Método de Integração Global - MIG) for improving motor and functional outcomes in children with autism spectrum disorder: a randomised controlled trial protocol
Source: Front Pediatr. 2026 Apr 22;14:1804826. doi: 10.3389/fped.2026.1804826 (PMC13144026; doi:10.3389/fped.2026.1804826)
Supplement: Supplementary file 2 [file Datasheet2.pdf]

JOÃO E O LIVRO DE AVENTURAS

Sessão 1

JOÃO ERA ESCOTEIRO. MAS SER ESCOTEIRO NÃO ERA SÓ USAR UM LENÇO NO PESCOÇO, CANTAR MÚSICAS DIVERTIDAS OU APRENDER A ACENDER FOGUEIRAS. ERA MUITO MAIS QUE ISSO!

SER ESCOTEIRO SIGNIFICAVA RESPEITAR TODAS AS PESSOAS, AJUDAR QUEM PRECISASSE E CUIDAR DA NATUREZA, COMO UM GUARDIÃO DA FLORESTA. TAMBÉM ERA SOBRE TRABALHAR EM EQUIPE, PORQUE, JUNTOS, OS ESCOTEIROS ERAM MAIS FORTES!

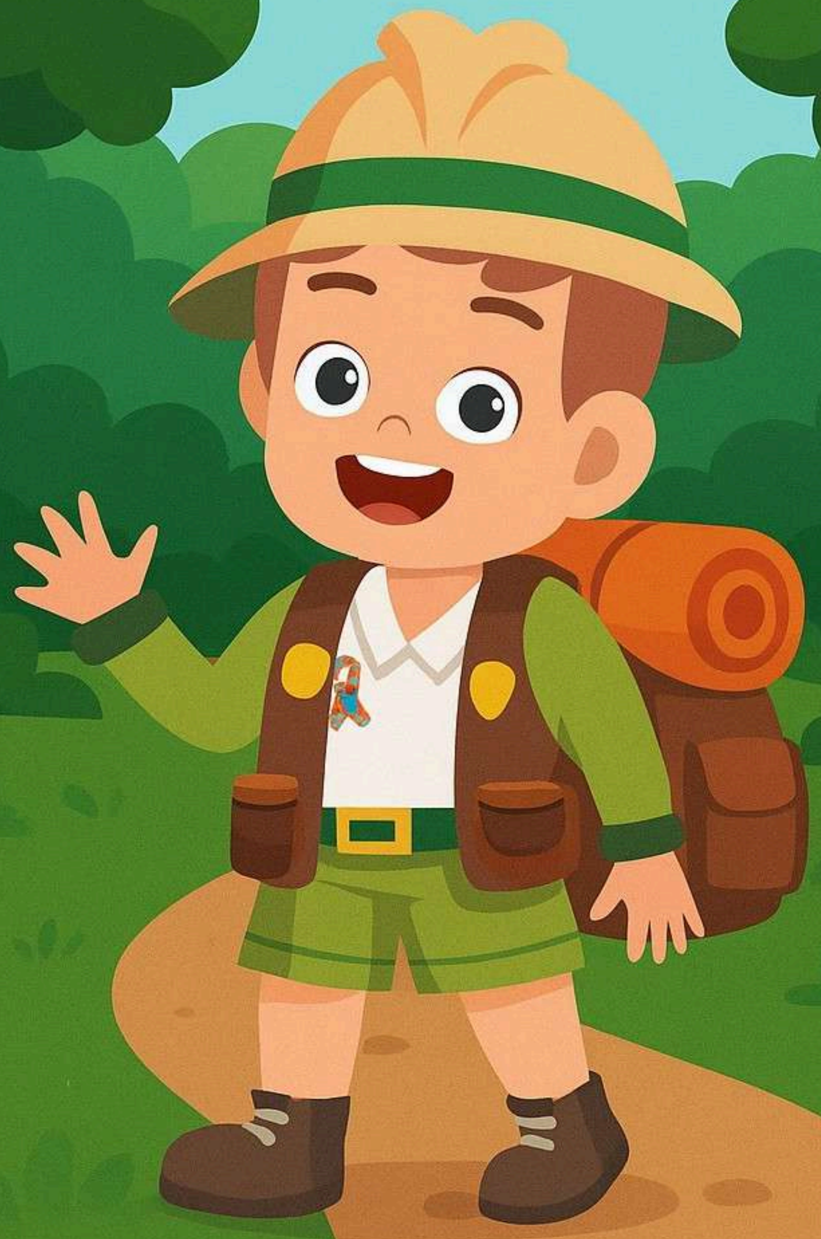

JOÃO ADORAVA EXPLORAR TRILHAS, MONTAR BARRACAS E DESCOBRIR COISAS NOVAS. GOSTAVA MUITO TAMBÉM DE FAZER AMIZADES E DE APRENDER COM O GRUPO SOBRE CORAGEM E RESPEITO.

PARA ELE, SER ESCOTEIRO ERA COMO VIVER UMA GRANDE AVENTURA, ONDE CADA DIA TRAZIA UMA DESCOBERTA DIFERENTE!

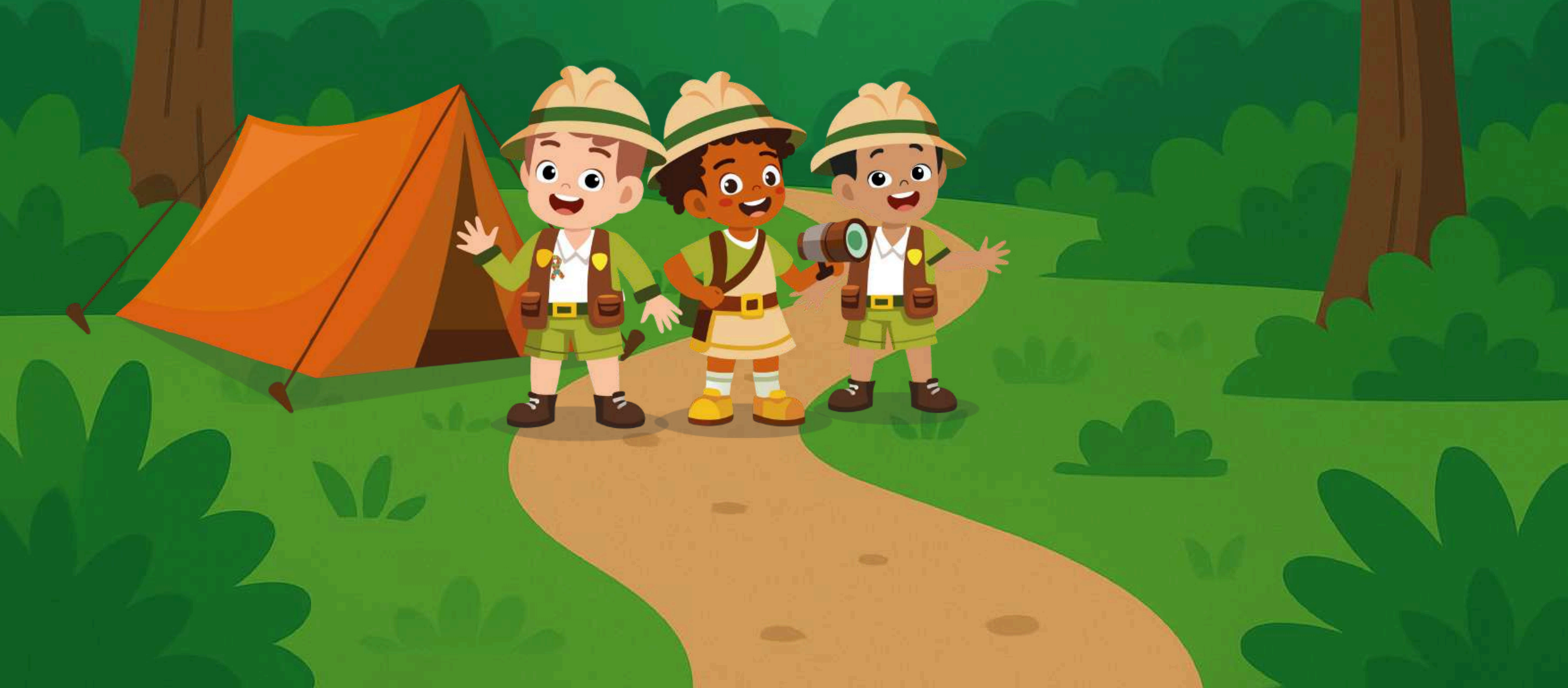

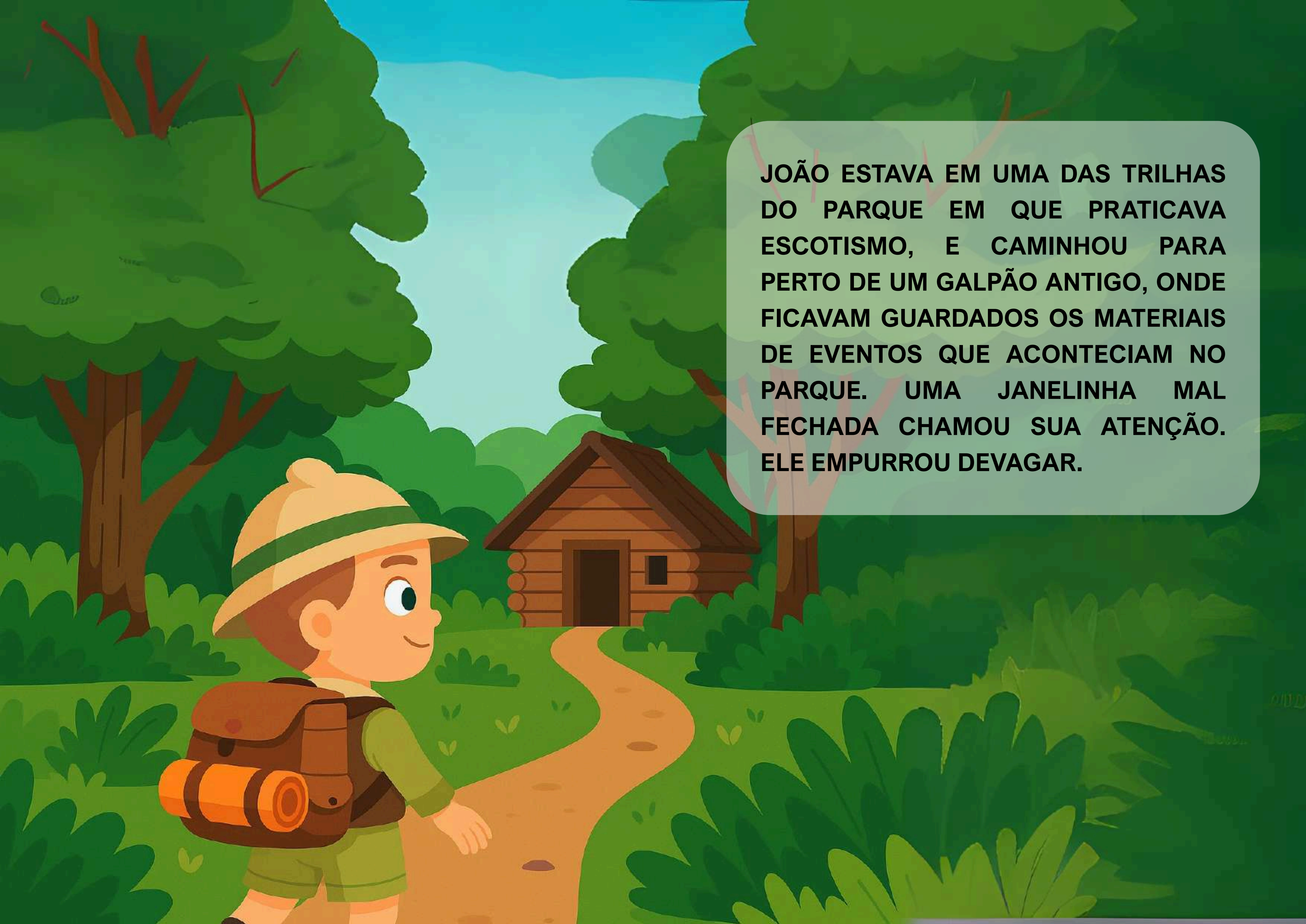

JOÃO ESTAVA EM UMA DAS TRILHAS DO PARQUE EM QUE PRATICAVA ESCOTISMO, E CAMINHOU PARA PERTO DE UM GALPÃO ANTIGO, ONDE FICAVAM GUARDADOS OS MATERIAIS DE EVENTOS QUE ACONTECIAM NO PARQUE. UMA JANELINHA MAL FECHADA CHAMOU SUA ATENÇÃO. ELE EMPURROU DEVAGAR.

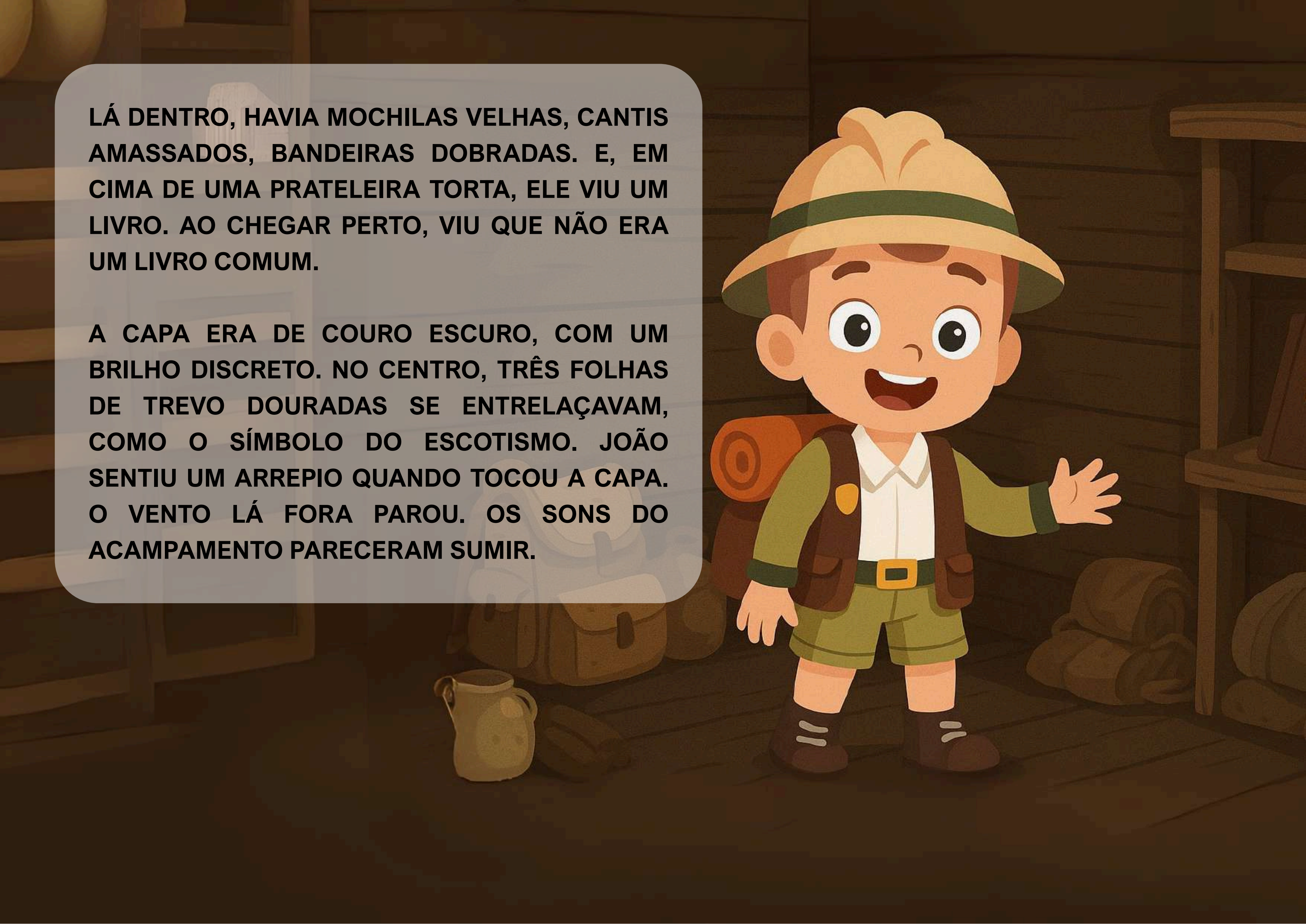

LÁ DENTRO, HAVIA MOCHILAS VELHAS, CANTIS AMASSADOS, BANDEIRAS DOBRADAS. E, EM CIMA DE UMA PRATELEIRA TORTA, ELE VIU UM LIVRO. AO CHEGAR PERTO, VIU QUE NÃO ERA UM LIVRO COMUM.

A CAPA ERA DE COURO ESCURO, COM UM BRILHO DISCRETO. NO CENTRO, TRÊS FOLHAS DE TREVO DOURADAS SE ENTRELAÇAVAM, COMO O SÍMBOLO DO ESCOTISMO. JOÃO SENTIU UM ARREPIO QUANDO TOCOU A CAPA. O VENTO LÁ FORA PAROU. OS SONS DO ACAMPAMENTO PARECERAM SUMIR.

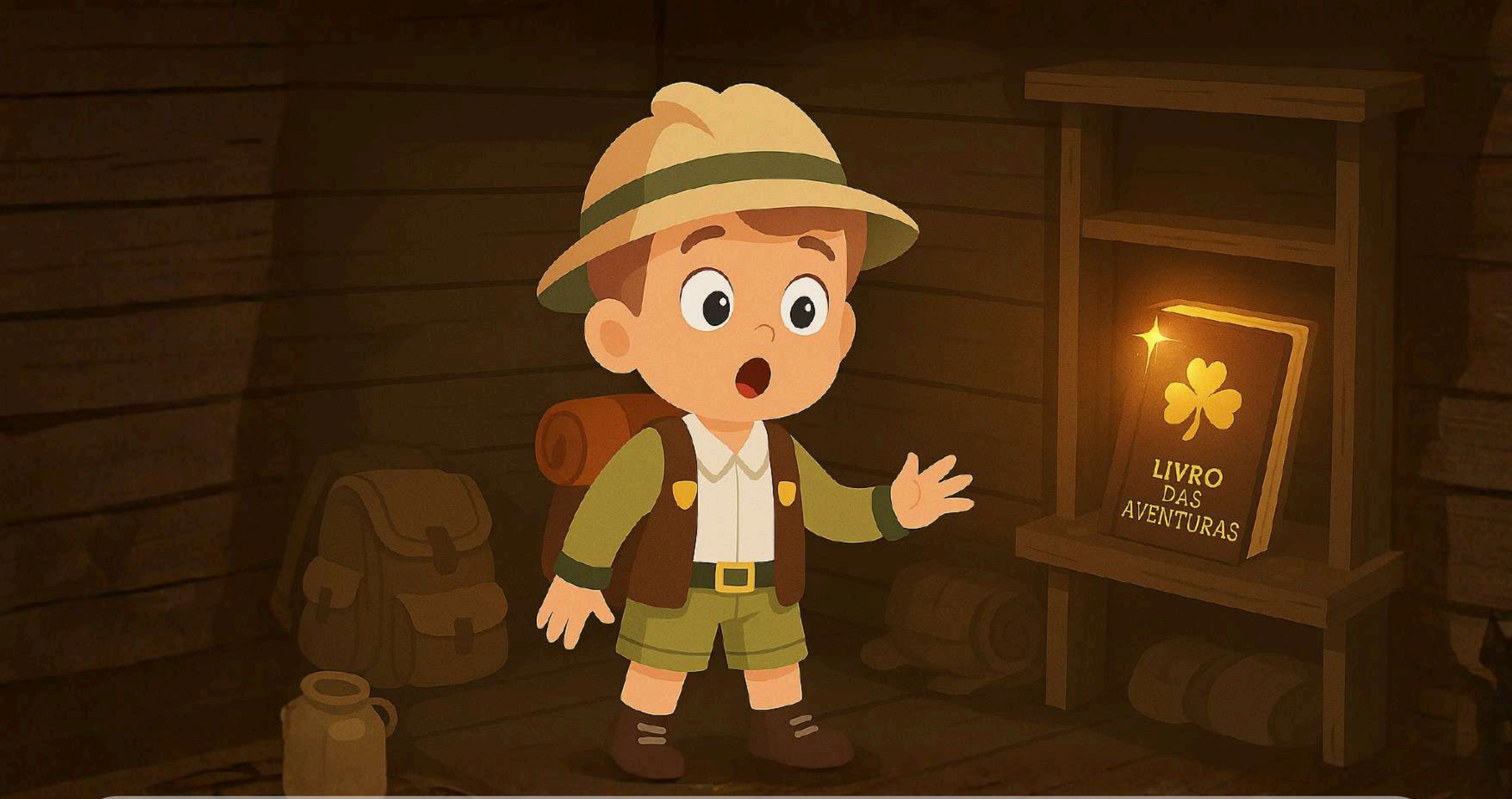

O LIVRO BRILHAVA E O SEGUINTE TÍTULO APARECEU: LIVRO DAS AVENTURAS.

JOÃO ABRIU A BOCA EM FORMA DE O, E SE AFASTOU DO LIVRO. ELE COMEÇOU A SENTIR MUITAS COISAS, QUE NÃO SABIA BEM O QUE ERA, MAS NÃO TIROU OS OLHOS DELE.

O LIVRO SE ABRIU E E ALGUMAS PALAVRAS FORAM SURGINDO:

OI JOÃO, COMO VOCÊ ESTÁ?

JOÃO RECUOU ALGUNS PASSOS E FICOU OLHANDO O LIVRO. SUA BOCA ESTAVA EM FORMATO DE O, E A SUA RESPIRAÇÃO ACELERADA IGUAL AS BATIDAS DO SEU CORAÇÃO. ELE SABIA QUE ESTAVA SENTINDO ALGO, MAS NÃO IDENTIFICAVA MUITO BEM.

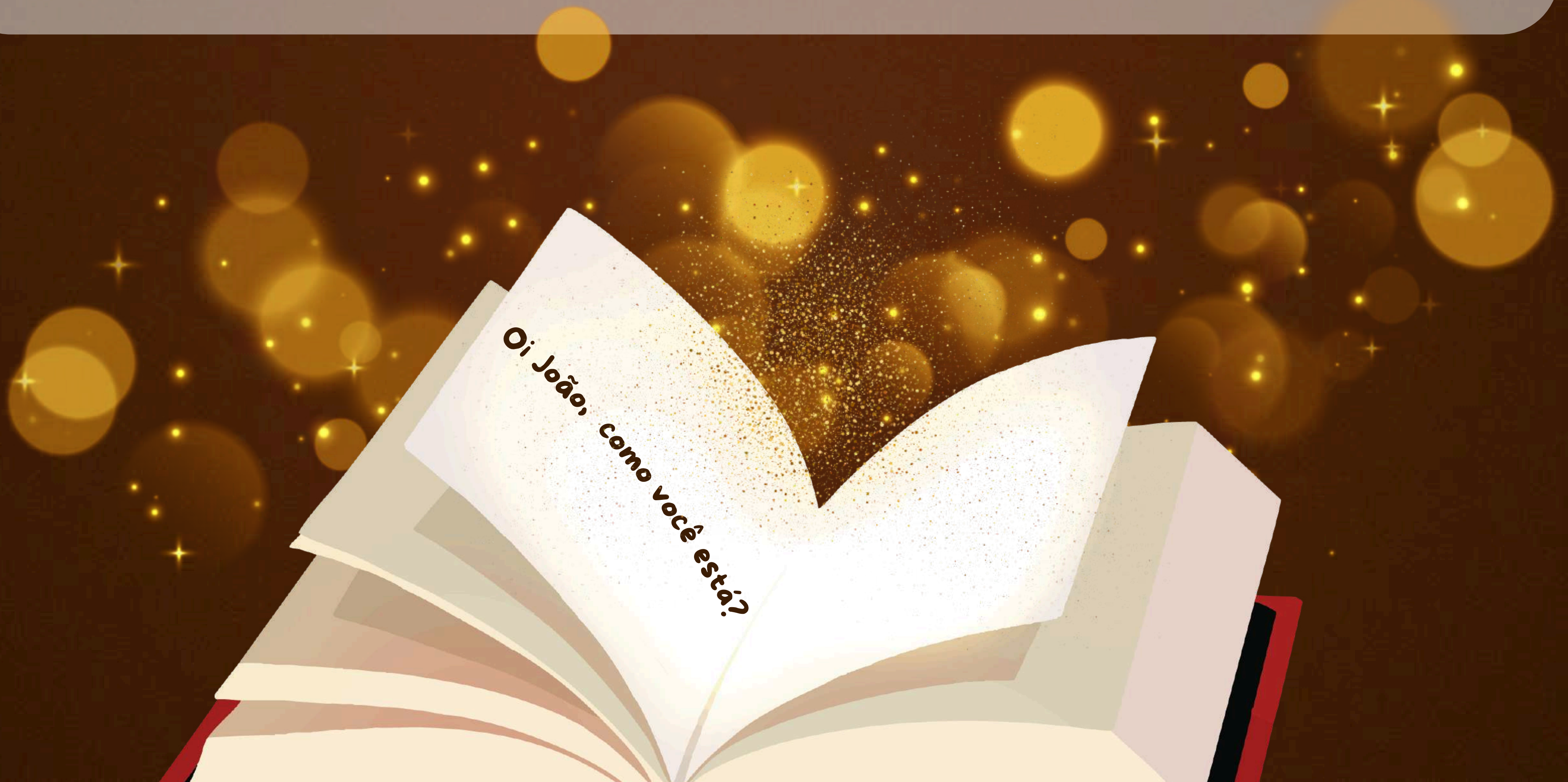

NOVAS PALAVRAS SURGIRAM NO LIVRO:

PARECE QUE VOCÊ FICOU BEM SURPRESO!

1. VOCÊ SABE O QUE SIGNIFICA FICAR SURPRESO?

2. O QUE VOCÊ SENTE QUANDO ALGO TE DEIXA ASSIM?

3. POR QUE JOÃO FICOU SURPRESO?

4. EM QUAIS SITUAÇÕES VOCÊ FICA SURPRESO?

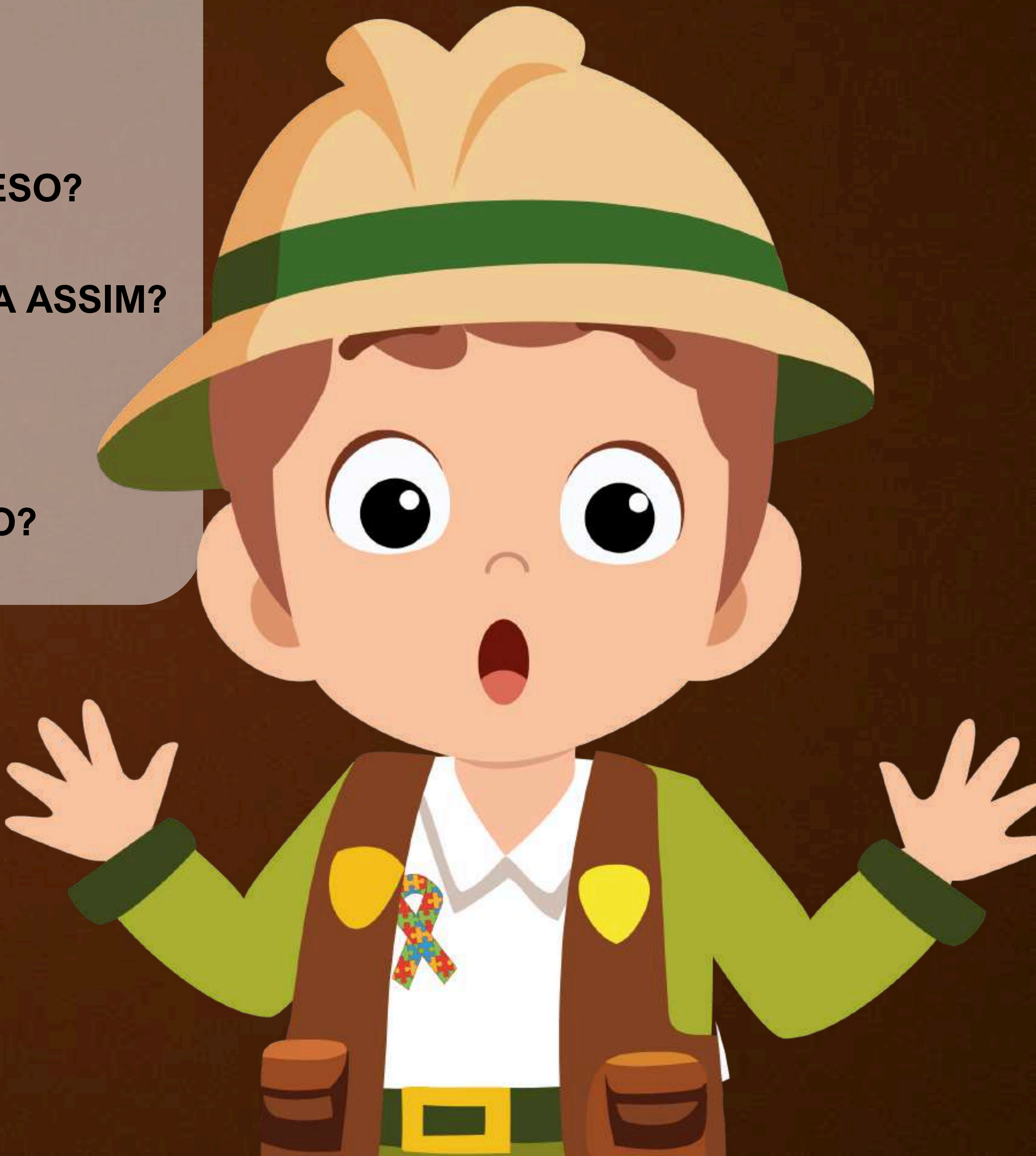

O LIVRO SE ABRIU E OUTRAS PALAVRAS FORAM SURGINDO:

"A SURPRESA É UMA EMOÇÃO QUE SURGE QUANDO ALGO ACONTECE DE REPENTE, E A GENTE NÃO ESPERAVA!"

JOÃO, AINDA COM SEU CORAÇÃO ACELERADO, MAS MUITO CURIOSO, PERGUNTOU AO LIVRO SE SEU CORAÇÃO E RESPIRAÇÃO ESTAVAM ACELERADOS PORQUE ELE ESTAVA SURPRESO.

PALAVRAS SURGIRAM:

SIM, ESSAS REAÇÕES SÃO COMUNS QUANDO FICAMOS MUITO SURPRESOS, MAS TAMBÉM PODEM ACONTECER COM OUTRAS EMOÇÕES!

COMO QUANDO ESTAMOS COM RAIVA! ELA É UMA EMOÇÃO MUITO FORTE QUE A GENTE SENTE QUANDO ALGO INJUSTO OU RUIM ACONTECE. VOCÊ JÁ SENTIU RAIVA?

JOÃO SE RECORDA QUE OUTRO DIA SENTIU RAIVA, QUANDO DERRUBARAM SUA MOCHILA E QUEBRARAM SUA BÚSSOLA.

1. O QUE SIGNIFICA SENTIR RAIVA?

2. COMO FICA NOSSO ROSTO QUANDO ESTAMOS COM RAIVA?

3. EM QUAIS SITUAÇÕES VOCÊ SENTE RAIVA?

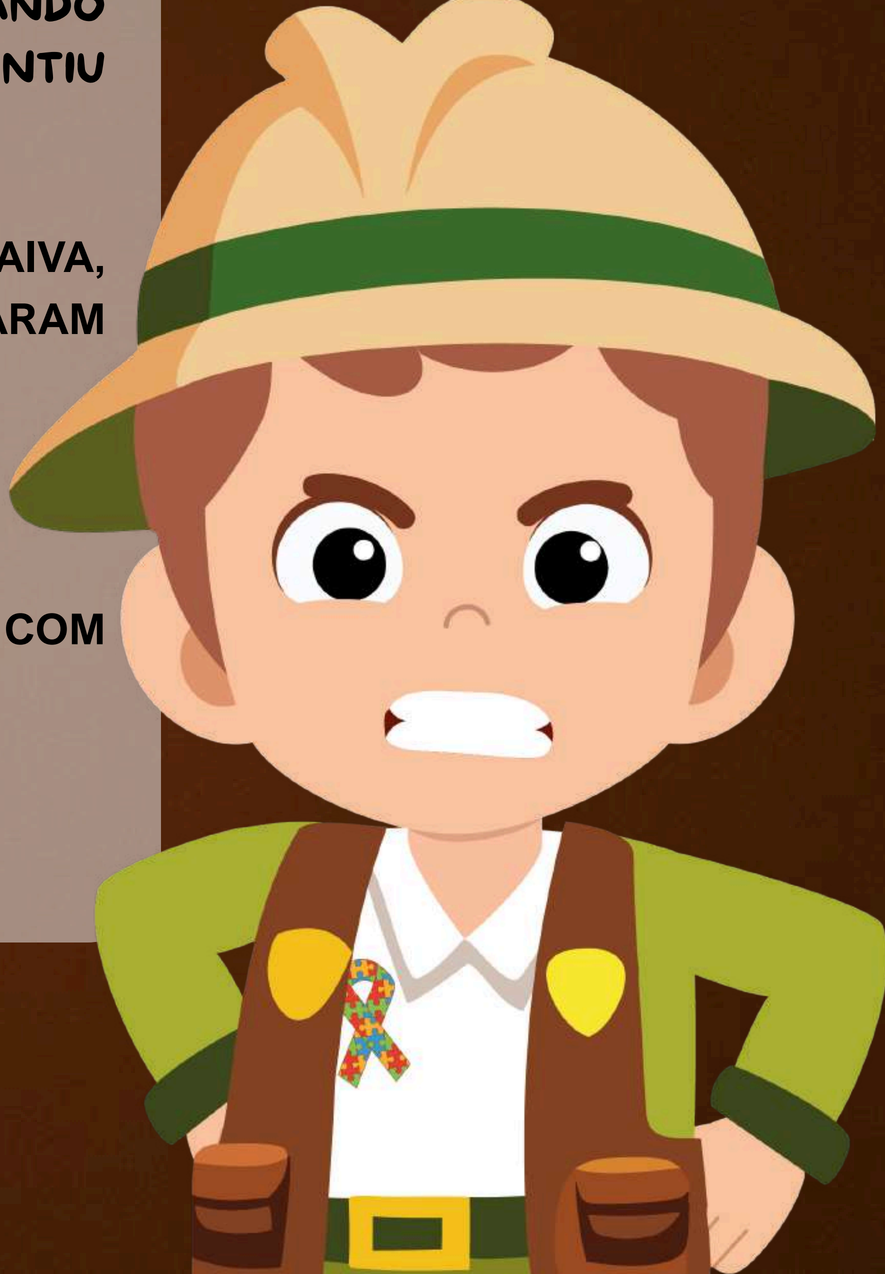

O LIVRO CONTINUA MOSTRANDO:

TAMBÉM TEM A ALEGRIA, ELA É UMA EMOÇÃO QUE A GENTE SENTE QUANDO ALGO BOM ACONTECE!

JOÃO LEMBROU DE MOMENTOS QUE VIVEU SENDO ESCOTEIRO:

- UM DIA EM QUE SENTIU ALEGRIA, PULANDO COM A SUA TROPA NAS POÇAS DE CHUVA.

1. O QUE SIGNIFICA SENTIR ALEGRIA?

2. COMO FICA NOSSO ROSTO QUANDO ESTAMOS ALEGRES?

3. EM QUAIS SITUAÇÕES VOCÊ SENTE ALEGRIA?

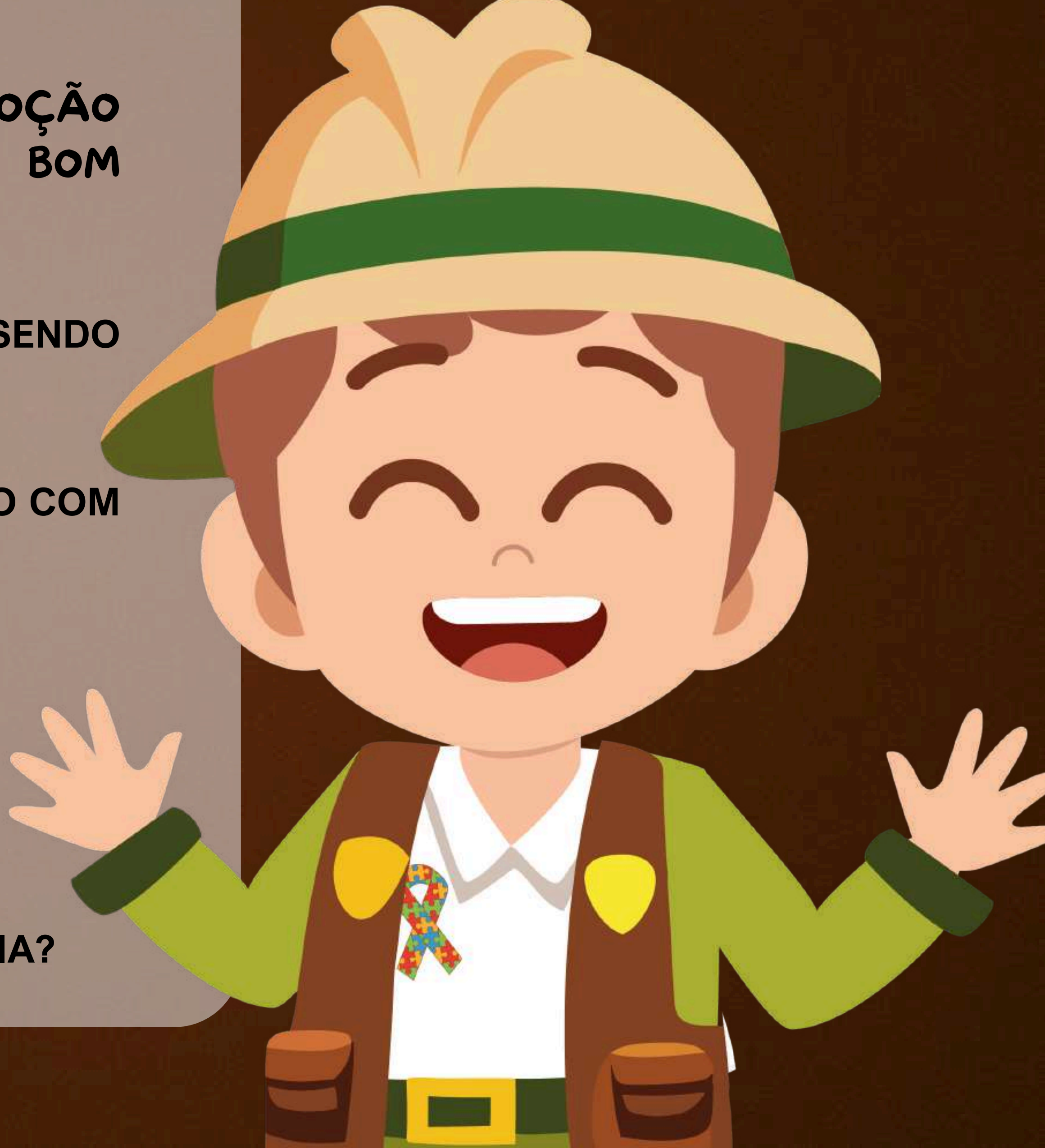

A TRISTEZA É UMA EMOÇÃO QUE A GENTE SENTE QUANDO ALGO RUIM OU DOLOROSO ACONTECE.

JOÃO SE LEMBROU QUE COMO SENTIU TRISTEZA, POR TER PERDIDO SUA BÚSSOLA DURANTE UMA TRILHA. ERA O ÚLTIMO PRESENTE QUE SEU AVÔ HAVIA LHE DADO.

1. O QUE SIGNIFICA SENTIR TRISTEZA?

2. COMO FICA NOSSO ROSTO QUANDO ESTAMOS TRISTES?

3. EM QUAIS SITUAÇÕES VOCÊ SENTE TRISTEZA?

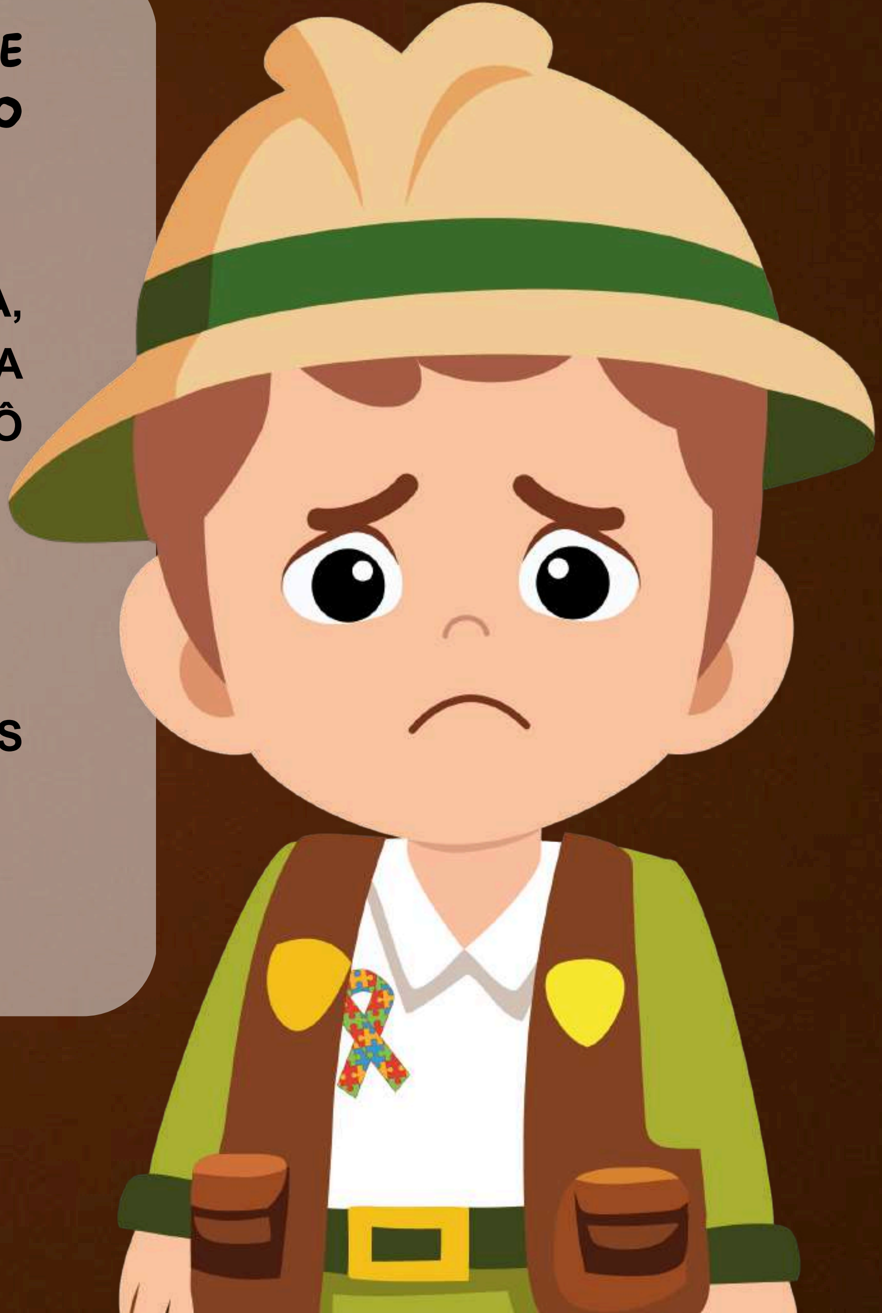

JÁ O MEDO SURGE QUANDO ALGO PARECE PERIGOSO OU DESCONHECIDO.

JOÃO SE LEMBROU DA TRILHA QUE FIZERAM NO ÚLTIMO MÊS, QUE VIRAM PEGADAS DE ANIMAIS DESCONHECIDOS E FICARAM ASSUSTADOS, E COM MEDO, RETORNANDO PARA O PARQUE SEM TERMINAR A TRILHA.

1. O QUE SIGNIFICA SENTIR MEDO?

2. COMO NOS SENTIMOS QUANDO ESTAMOS COM MEDO?

3. EM QUAIS SITUAÇÕES VOCÊ SENTE MEDO?

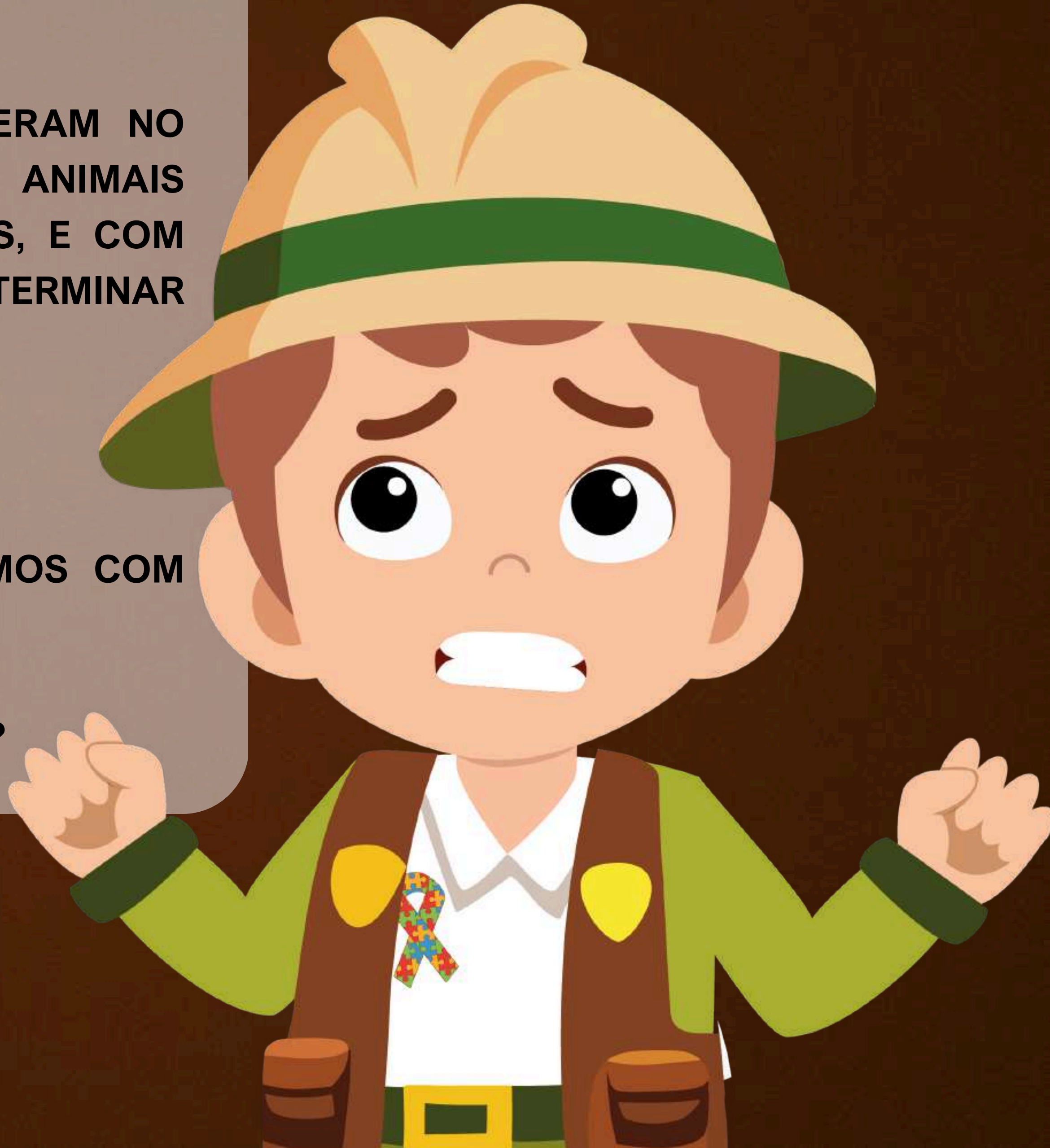

OUTRA EMOÇÃO É O NOJO, EMOÇÃO QUE SENTIMOS QUANDO VEMOS COISAS QUE PARECEM NOJENTAS OU PERIGOSAS PARA A SAÚDE.

JOÃO SE LEMBROU DE TER SENTIDO NOJO, AO LIMPAR UMA PANELA COM RESTOS DE COMIDA ESTRAGADA, QUE ESQUECERAM A NOITE EM UM ACAMPAMENTO..

1. O QUE SIGNIFICA SENTIR NOJO?

2. COMO FICA NOSSO ROSTO QUANDO ESTAMOS COM NOJO?

3. EM QUAIS SITUAÇÕES VOCÊ SENTE NOJO?

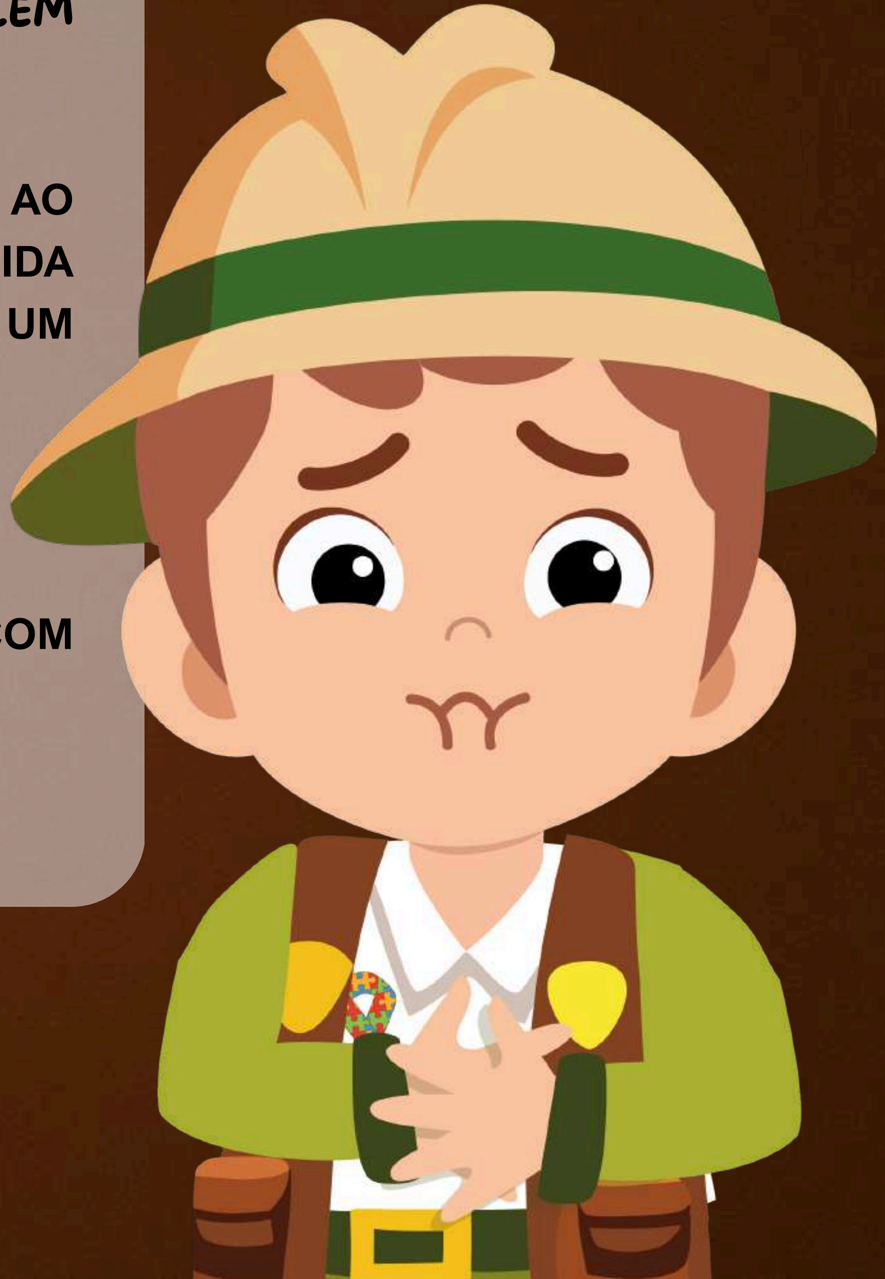

VAMOS CONVERSAR

1. Qual é o personagem da história?
2. Qual é a situação inicial?
3. Qual é o problema da história? O que aconteceu?
4. Como o personagem se sentiu?

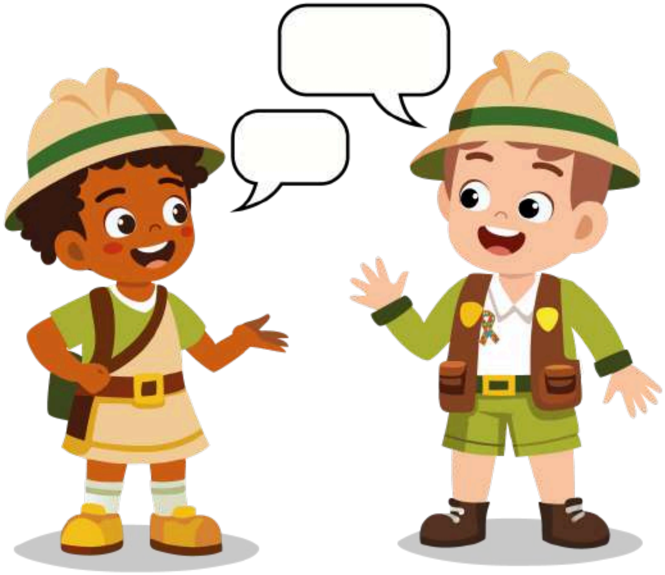

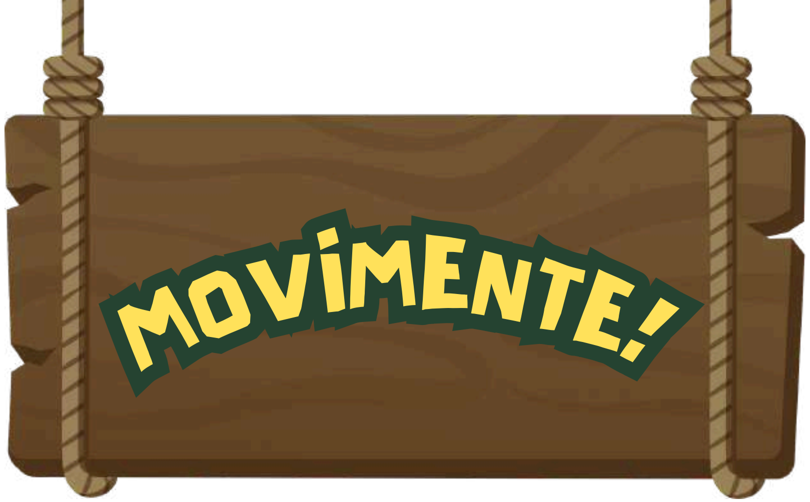

1. Tem muitas caixas velhas na frente do Livro das Aventuras. Retire as caixas da frente do livro e coloque atrás de você.

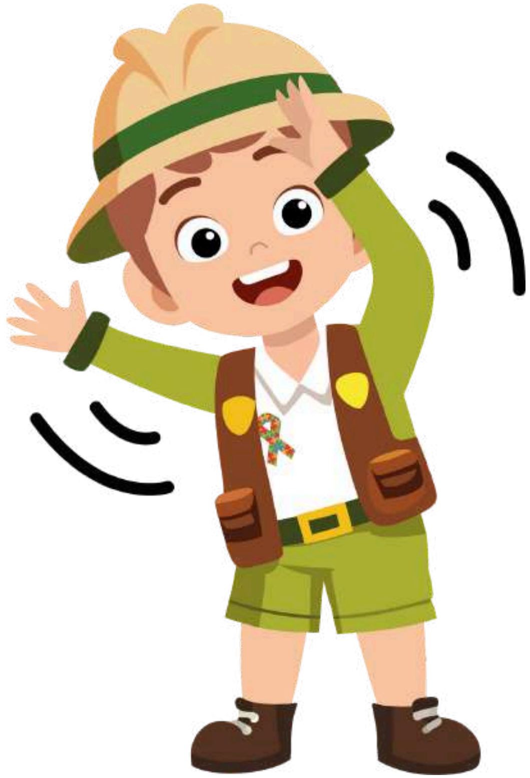

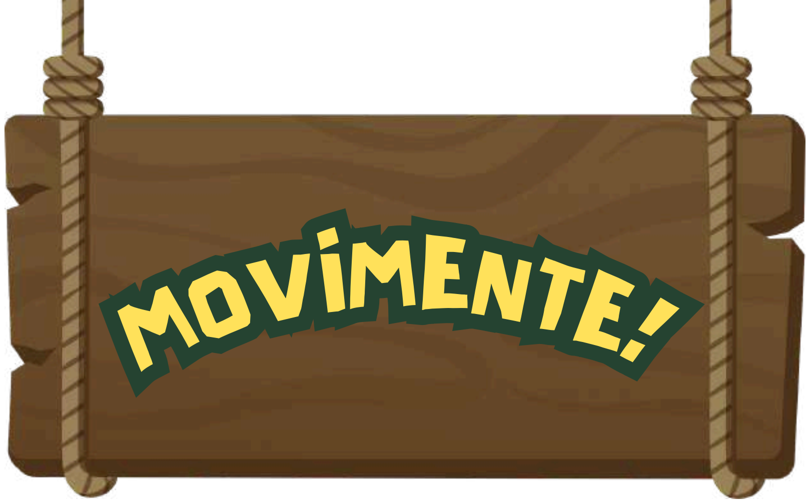

2. O Livro de Aventuras escorregou e caiu atrás da prateleira antiga no galpão. Agora ele está coberto por objetos antigos. Para ajudar João, deite-se no chão, alcance os objetos acima da cabeça e, usando a força da barriga, suba e coloque cada item no balde entre as pernas.

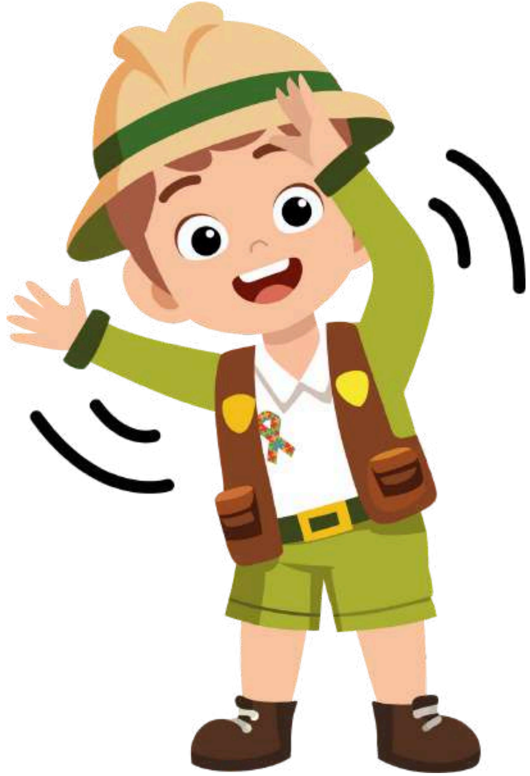

JOÃO ABRIU O LIVRO. AS PÁGINAS ESTAVAM EM BRANCO, MAS PULSAVAM. PEGOU O LÁPIS E ESCREVEU:

"HOJE SENTI MUITAS COISAS AO MESMO TEMPO: SURPRESA, MEDO, ALEGRIA, TRISTEZA, RAIVA E NOJO. FIQUEI CONFUSO."

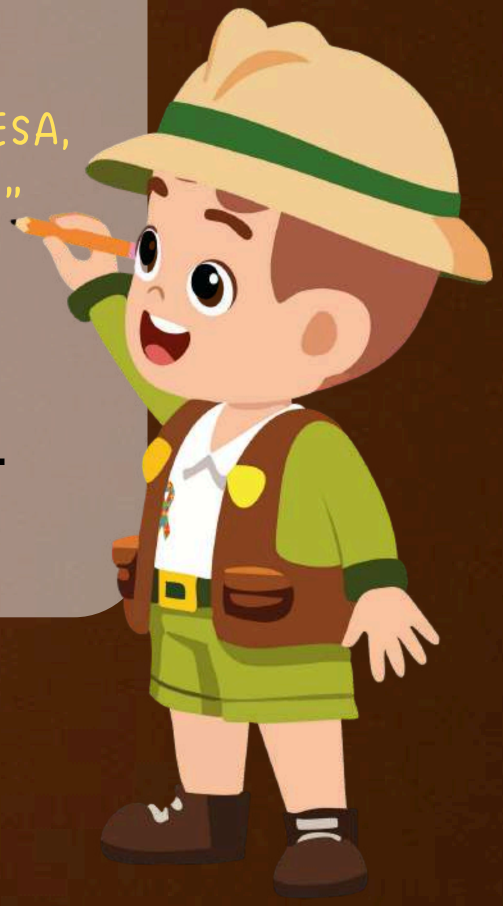

NA HORA, O LIVRO BRILHOU. AS PÁGINAS VIRARAM SOZINHAS.

TRÊS CAMINHOS SURGIRAM DIANTE DELE:

1. ESCONDER AS EMOÇÕES E FINGIR QUE ESTÁ TUDO BEM.
2. SE OCUPAR O DIA TODO PARA NÃO PENSAR NO QUE SENTE.
3. ESCREVER O QUE SENTE PARA ENTENDER MELHOR E CONVERSAR COM ALGUÉM DE CONFIANÇA.

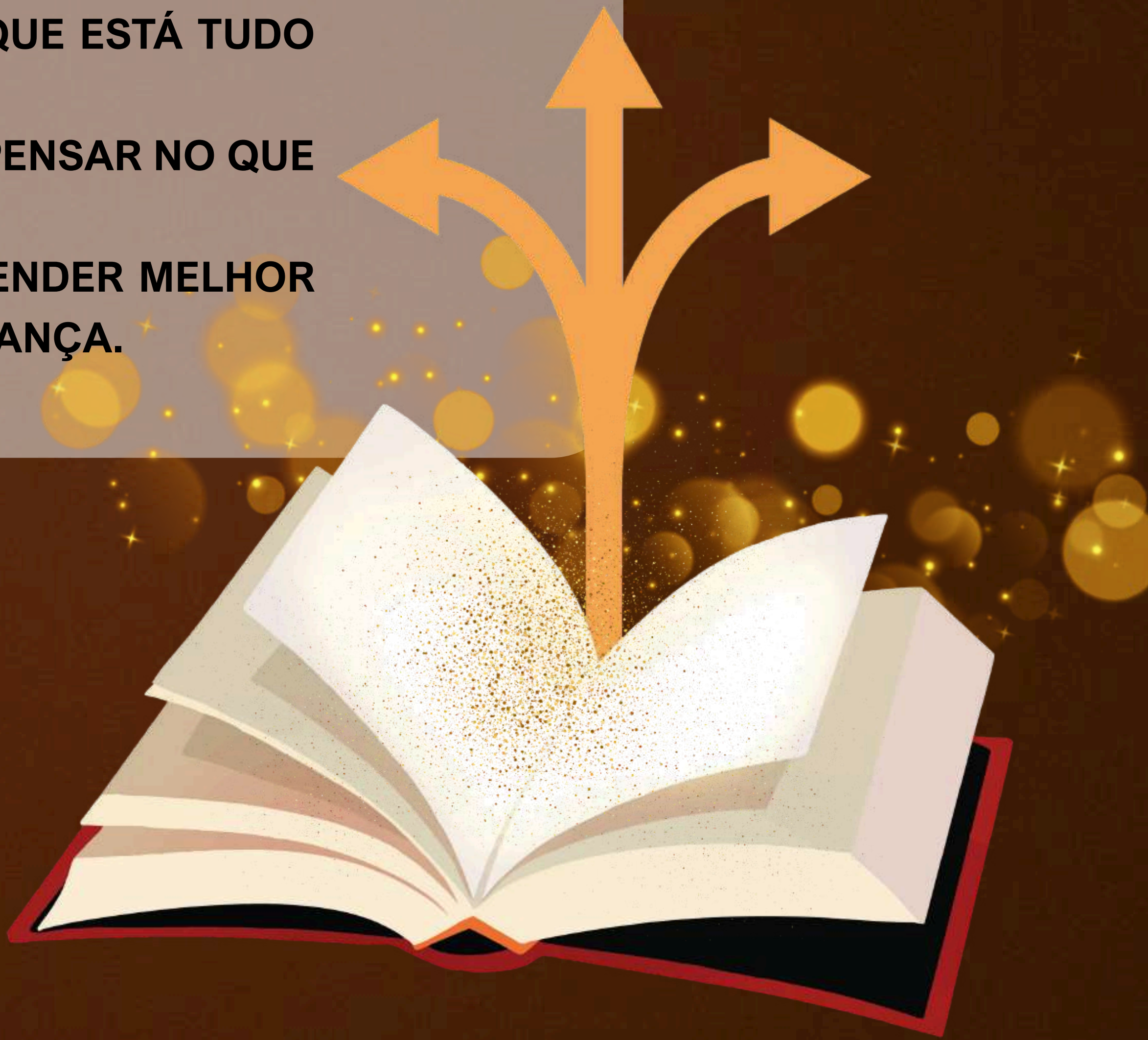

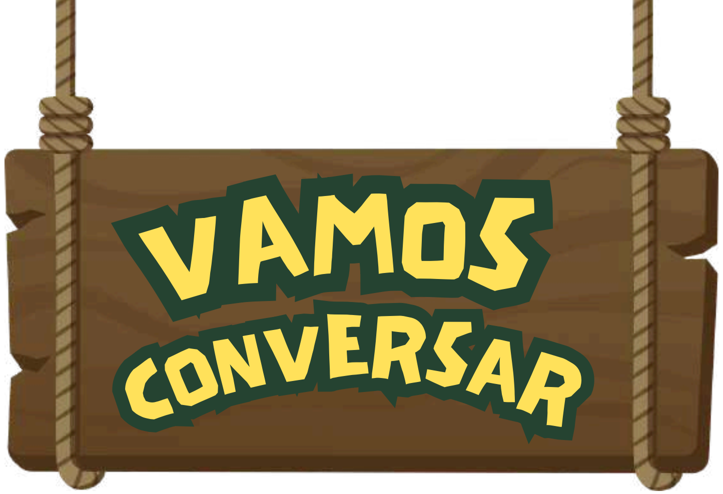

1. O que você acha que vai acontecer se João seguir cada um desses caminhos?

2. Qual é a melhor opção?

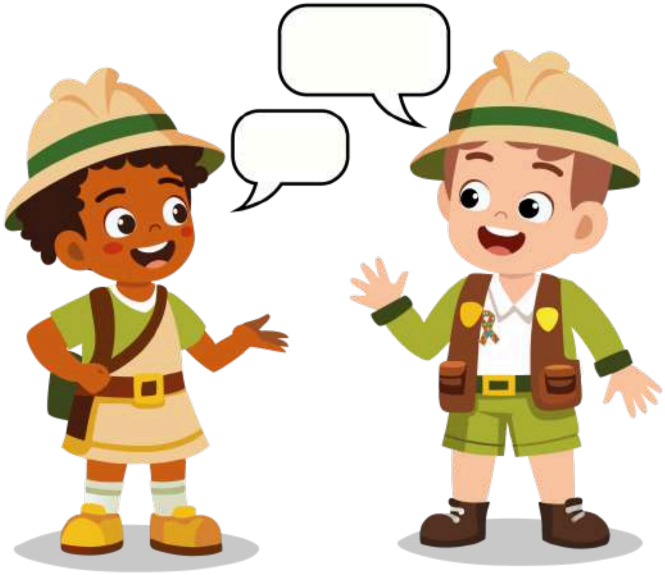

JOÃO LEU COM ATENÇÃO. CADA ESCOLHA VINHA COM DESENHOS: UMA NUVEM ESCURA QUE ESCONDIA O SOL, UM CORAÇÃO ACELERADO, UM ABRAÇO DE UM AMIGO.

ELE OLHOU COM CALMA PARA CADA UM DELES. SENTIA O CORAÇÃO MAIS QUIETO, COMO SE O LIVRO O AJUDASSE A ENTENDER TUDO O QUE PODE SER FEITO.

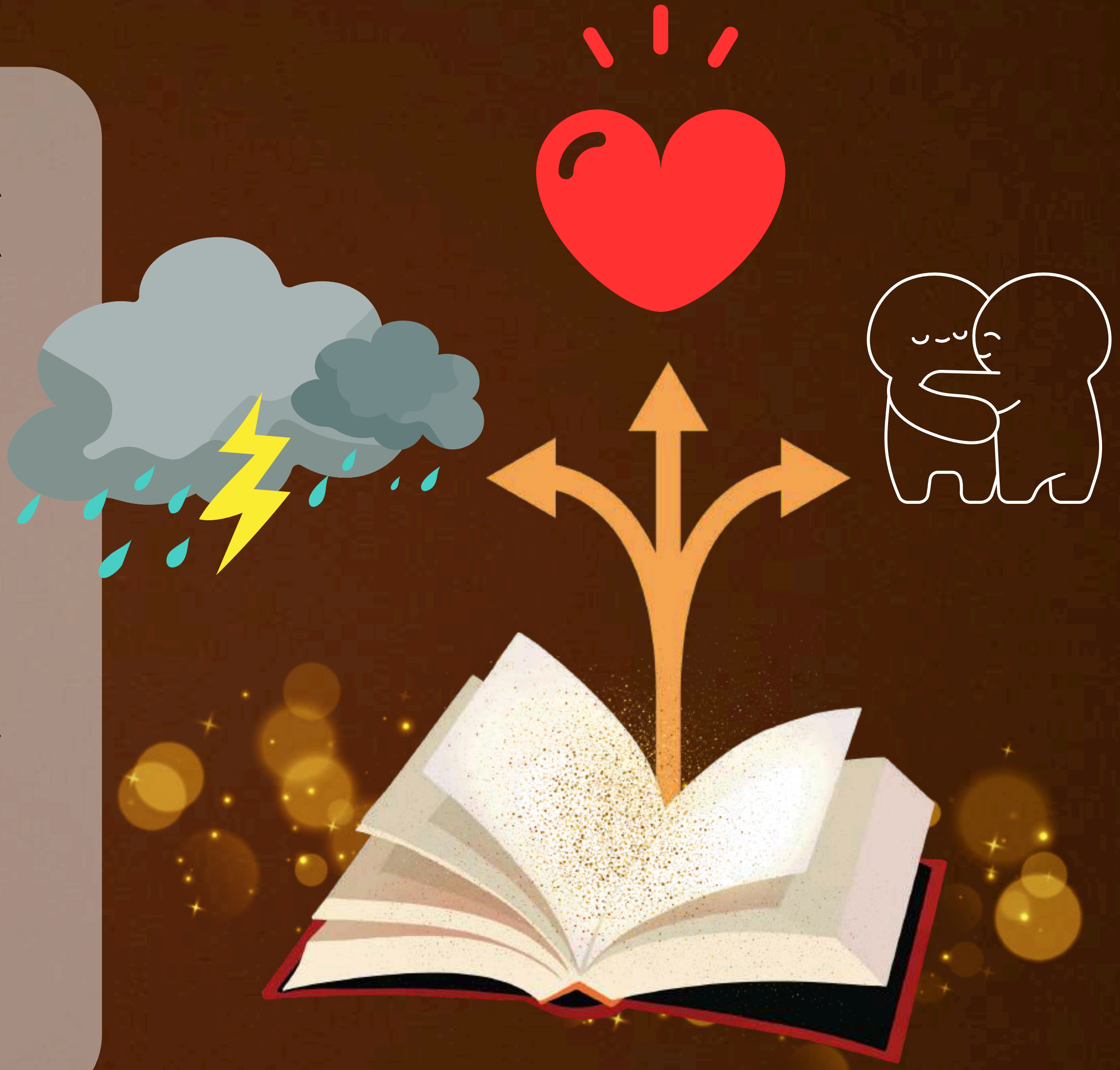

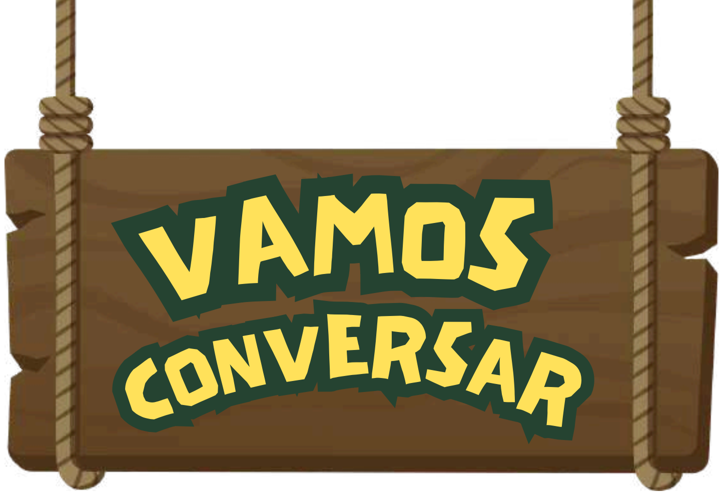

1. Quais podem ser os planos de João para resolver o problema?

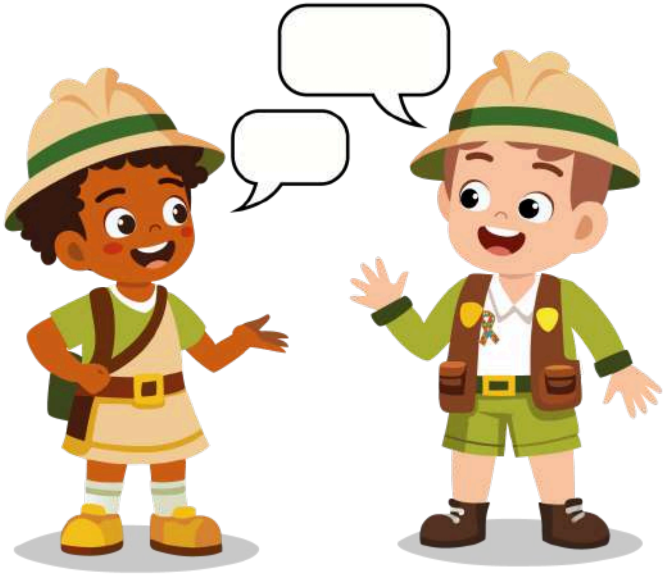

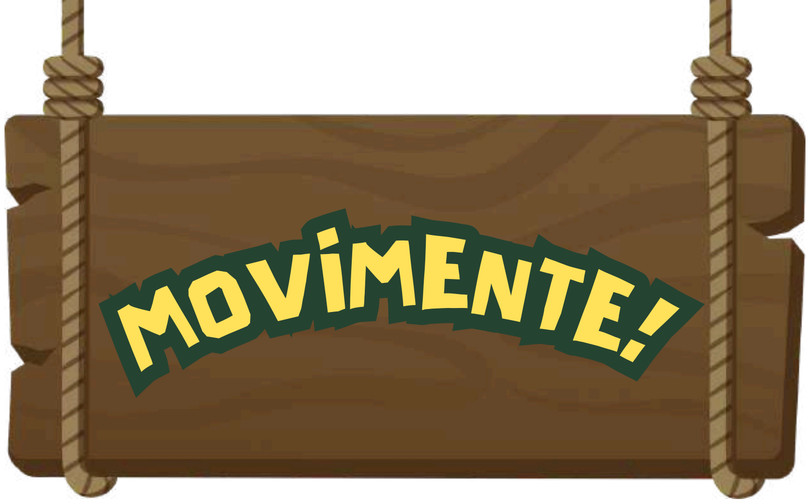

1. João tentou esconder seus sentimentos. Ele foi brincar de recolher argolas com a sua tropa.

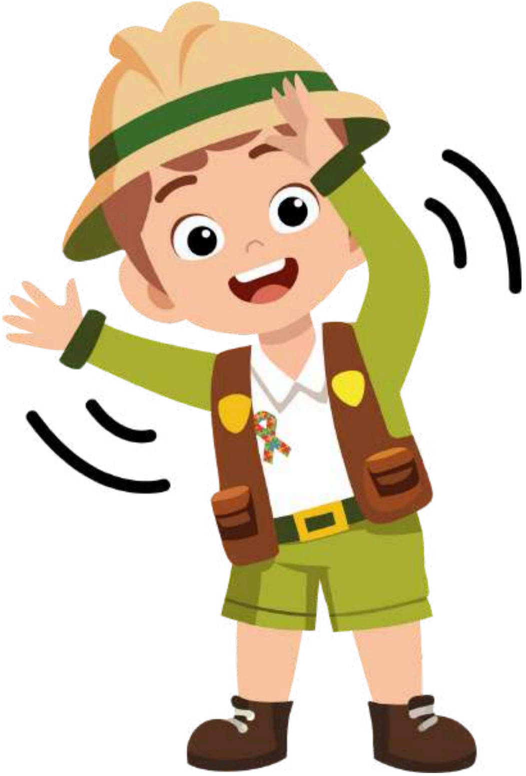

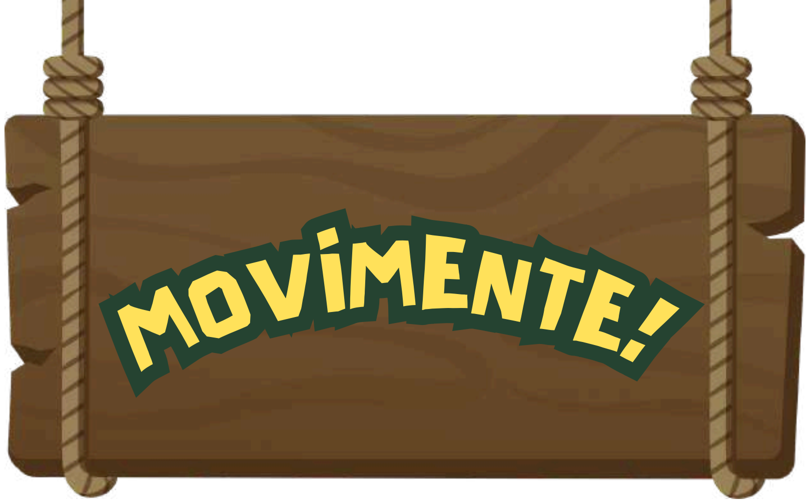

2. João tentou fazer muitas coisas para se distrair. Ele decidiu fazer uma caminhada em uma ponte muito estreita.

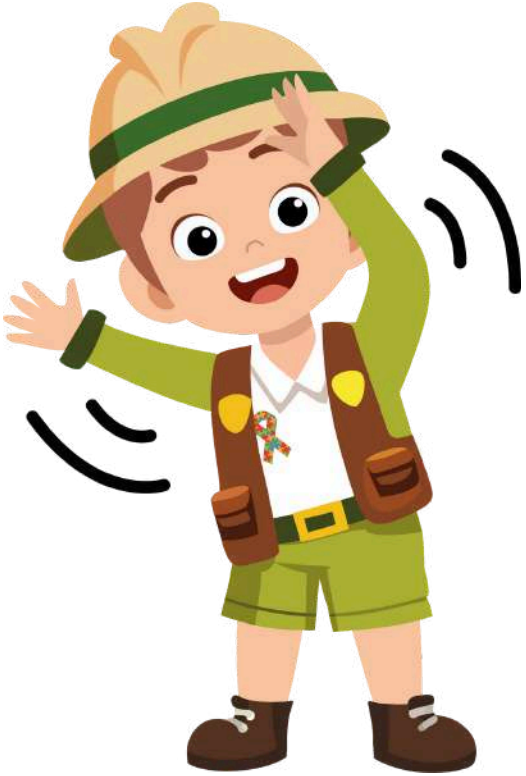

MOVIMENTE!

3. João tentou fazer muitas coisas para se distrair. Ele decidiu preparar a fogueira do grupo. João precisa juntar pequenos gravetos espalhados pelo chão.

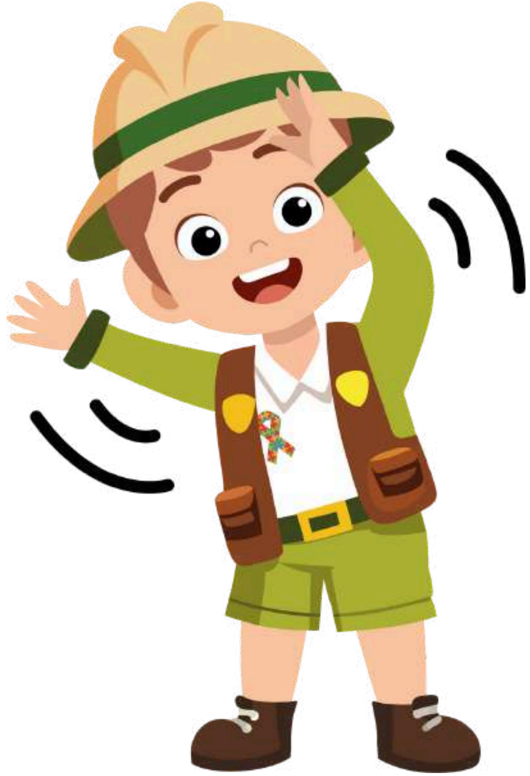

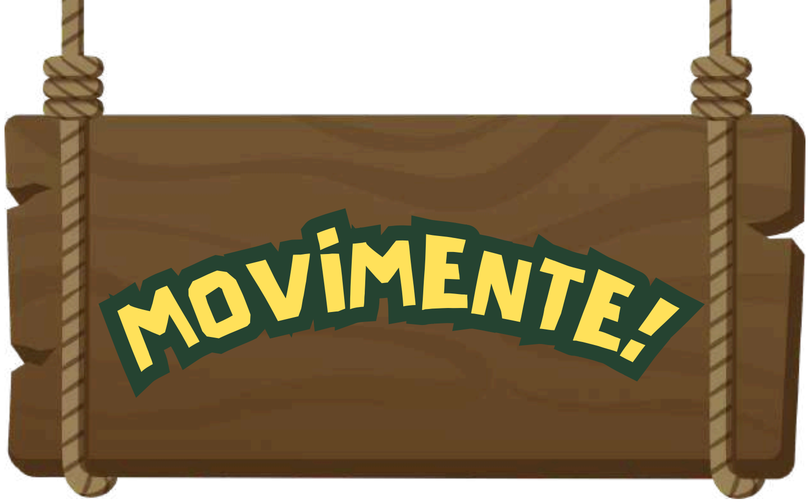

4. João decidiu escrever o que sente e conversar com alguém de confiança. Jogue bola com seu amigo enquanto conversa.

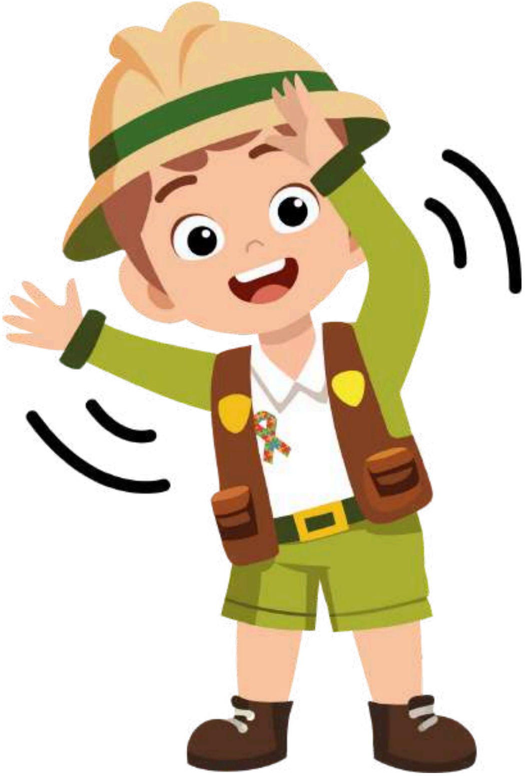

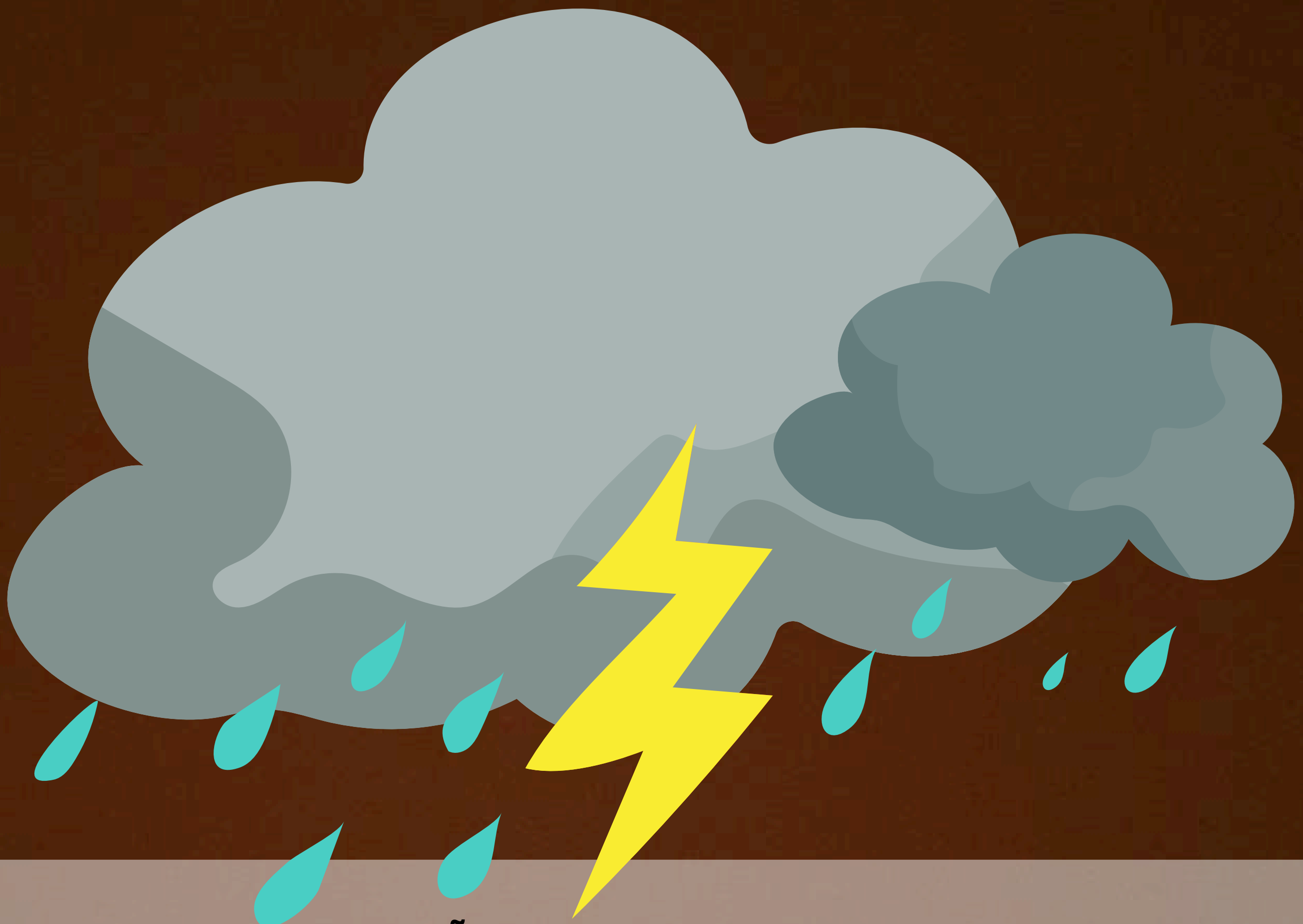

JOÃO TENTOU ESCONDER AS SUAS EMOÇÕES, E FINGIR QUE ESTAVA TUDO BEM. ELE FOI BRINCAR COM A SUA TROPA, MAS TODOS PERCEBERAM QUE ELE ESTAVA MAL E PERGUNTARAM O QUE ERA.

JOÃO TENTOU SE OCUPAR COM MUITAS COISAS PARA NÃO PENSAR, COMO FAZER CAMINHADAS E AJUNTAR GRAVETOS. MAS ELE CONTINUOU SE SENTINDO MAL.

ENTÃO, JOÃO DECIDIU ESCREVER SEUS SENTIMENTOS E PROCURAR UM AMIGO PARA CONVERSAR.

COM O LÁPIS NA MÃO, ESCREVEU NA PRIMEIRA PÁGINA DO LIVRO DAS AVENTURAS:

"HOJE SENTI MUITAS EMOÇÕES AO MESMO TEMPO. PENSEI QUE FOSSE SÓ CONFUSÃO, MAS ERA SURPRESA, MEDO, TRISTEZA, RAIVA E ALEGRIA MISTURADAS. ESCREVER ME AJUDOU A ENTENDER E DAR NOME PARA O QUE EU SINTO. ME SENTI MELHOR."

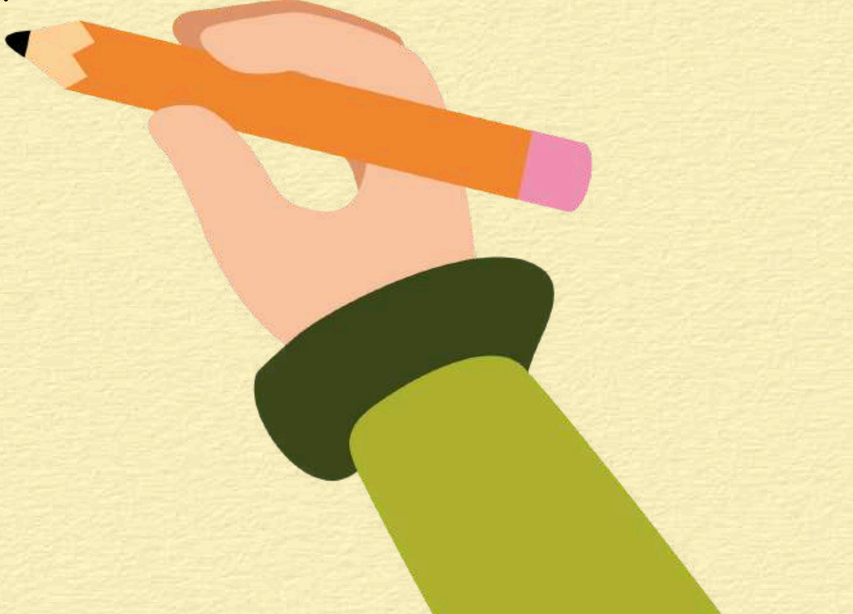

JOÃO VOLTOU DEVAGAR PARA PERTO DAS BARRACAS. VIU PEDRINHO, UM AMIGO DA TROPA, SENTADO PERTO DA MOCHILA, AMARRANDO O TÊNIS. JOÃO SE APROXIMOU. EI, POSSO TE CONTAR UMA COISA ESTRANHA QUE ACONTECEU HOJE?

PEDRINHO CONCORDOU, CURIOSO. ACORDEI CEDO, E AÍ COMECEI A LEMBRAR DE UM MONTE DE COISA... E SENTI UM MONTE DE COISA AO MESMO TEMPO. ALEGRIA, RAIVA, NOJO, TRISTEZA, MEDO... TUDO EMBOLADO. FIQUEI CONFUSO.

PEDRO FICOU EM SILÊNCIO, ESCUTANDO.

AÍ EU ENCONTREI UM LIVRO... É DIFÍCIL DE EXPLICAR... MAS ELE ME AJUDOU A VER O QUE EU PODERIA FAZER. QUANDO ESCREVI O QUE SENTIA, PARECIA QUE AS EMOÇÕES SE ORGANIZAVAM AQUI DENTRO. AINDA SINTO ALGUMAS DELAS… MAS AGORA SEI O NOME DE CADA UMA.

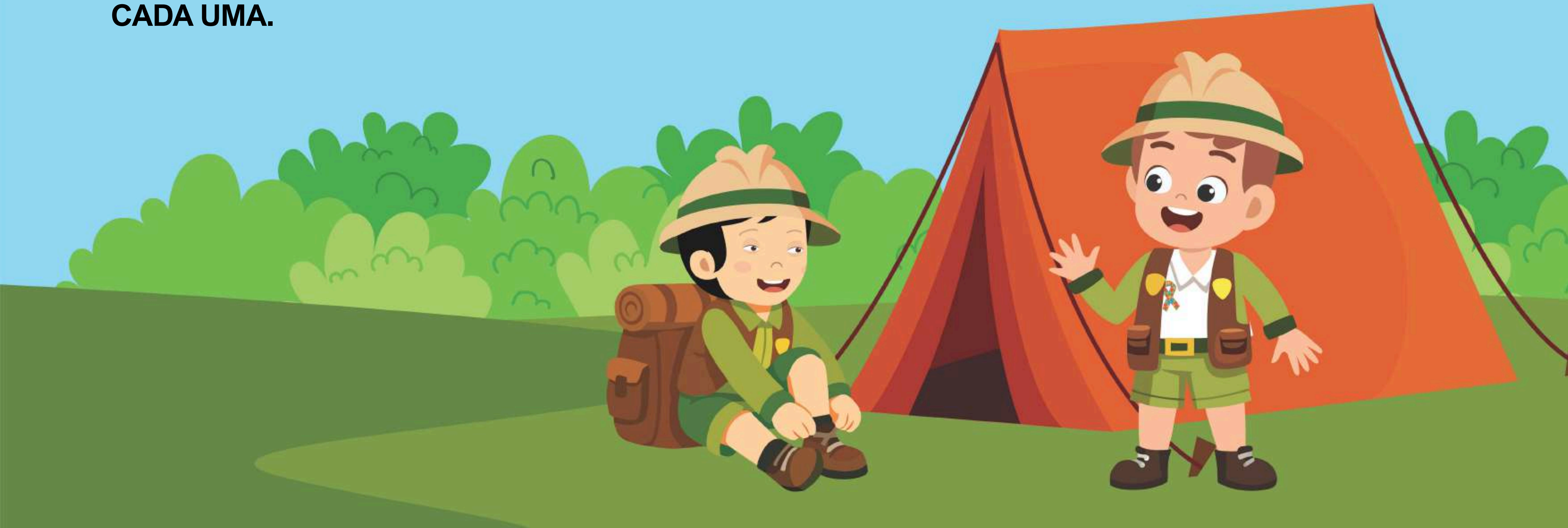

PEDRO SORRIU.

ACHO QUE ISSO ACONTECE COMIGO TAMBÉM. QUANDO FICO NERVOSO E NÃO ENTENDO POR QUÊ. NUNCA PENSEI EM ESCREVER.

JOÃO SORRIU DE VOLTA.

SENTIU QUE, QUANDO A GENTE ENTENDE O QUE SENTE — E CONVERSA COM ALGUÉM — AS EMOÇÕES CONTINUAM LÁ, MAS FICAM MAIS LEVES.

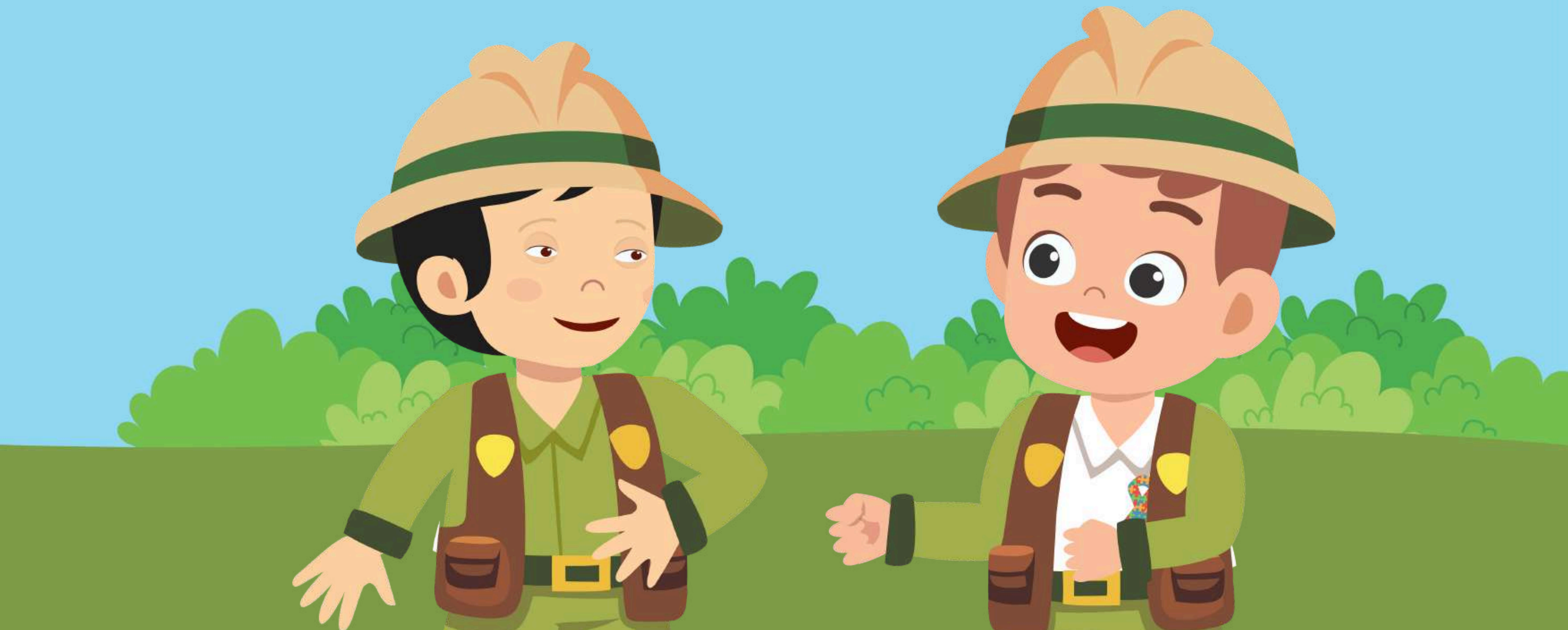

VAMOS CONVERSAR

1. Quais planos João colocou em prática?
2. Qual foi o melhor plano? Por que?
3. Qual foi a ação de João?
4. Qual é a situação final da história?
5. Reconte a história com as suas palavras.

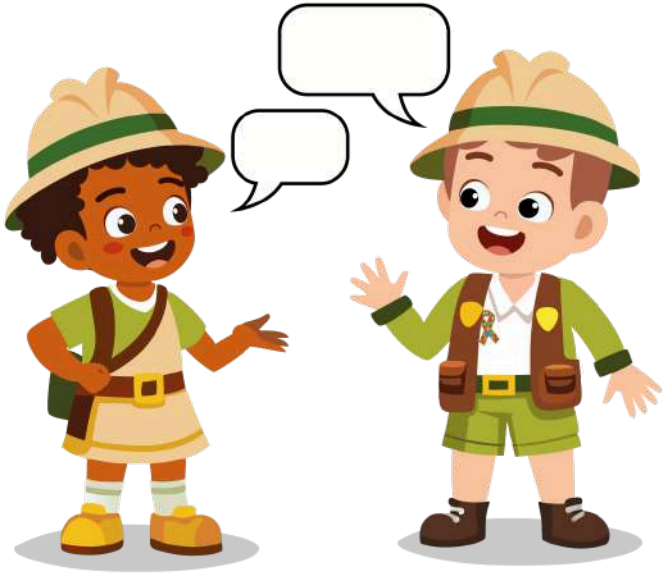

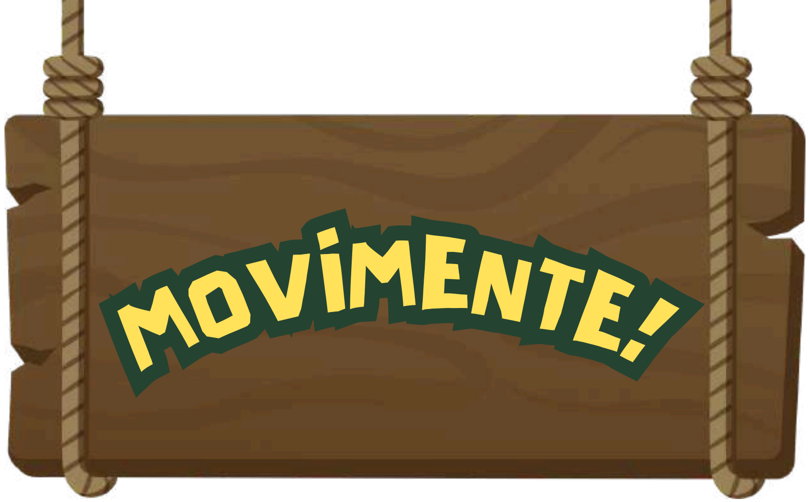

1. João agora se sentia melhor para brincar com a sua tropa. Os amigos foram brincar de arremessar a bola na parede.

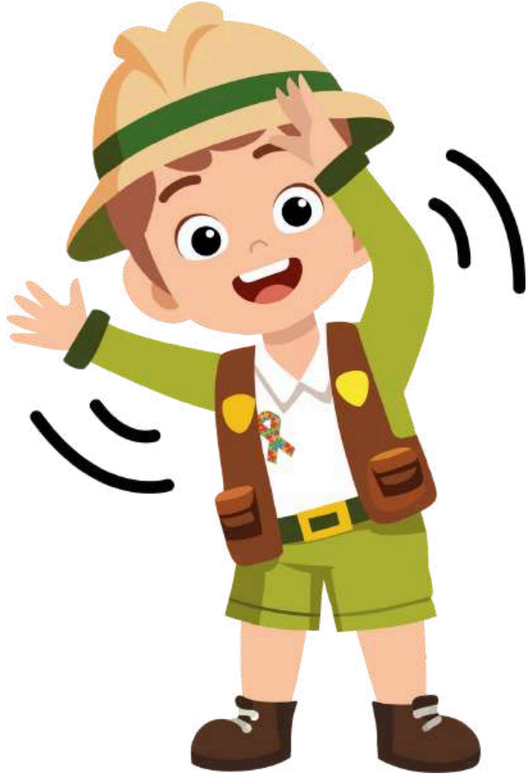

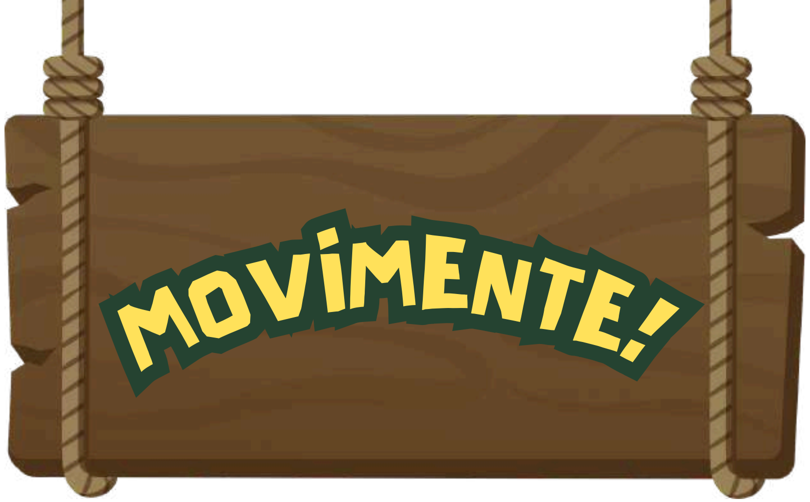

2. Depois, a tropa decidiu apostar quem conseguia andar mais rápido na ponte estreita.

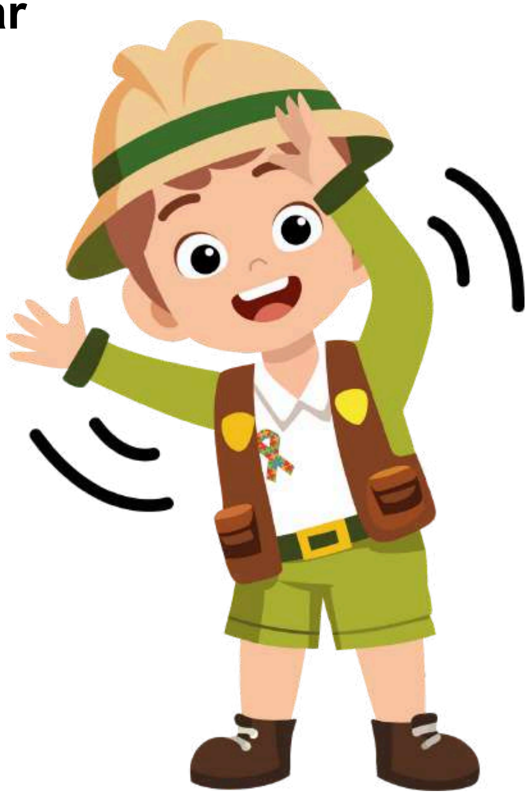

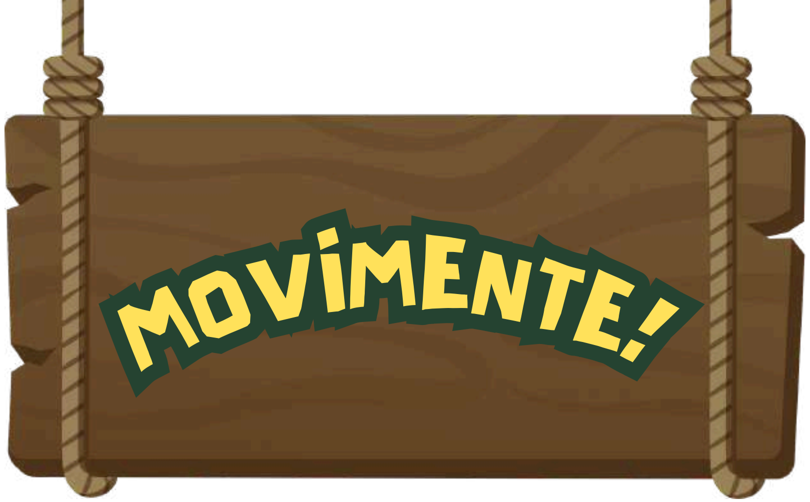

3. Por fim, a tropa decidiu correr no acampamento, se desviando de obstáculos.

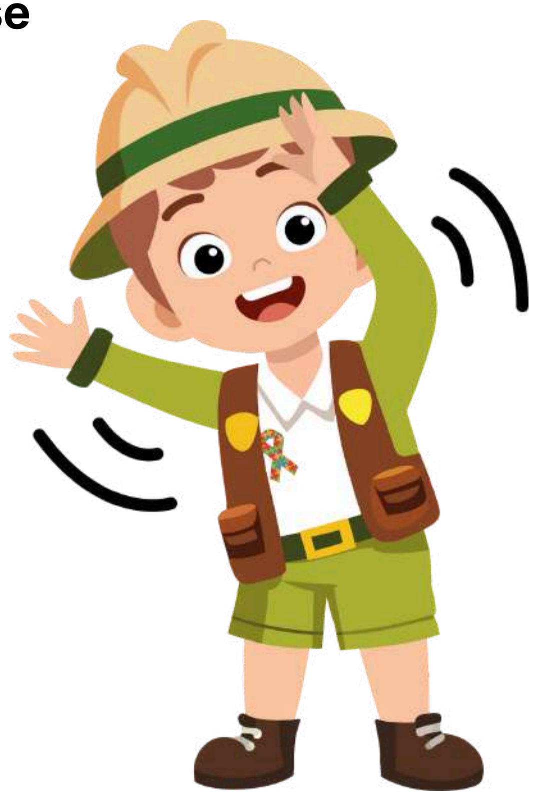

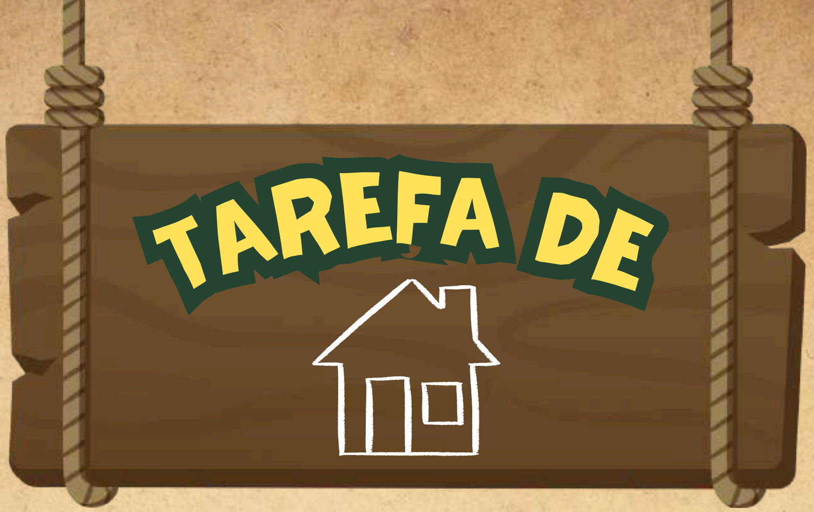

DESENHE NO LIVRO DE AVENTURAS SITUAÇÕES EM QUE VOCÊ SINTA CADA UMA DAS EMOÇÕES:

- **ALEGRIA**
- **TRISTEZA**
- **RAIVA**
- **MEDO**
- **NOJO**
- **SURPRESA**

JOÃO E O LIVRO DE AVENTURAS

Sessão 15

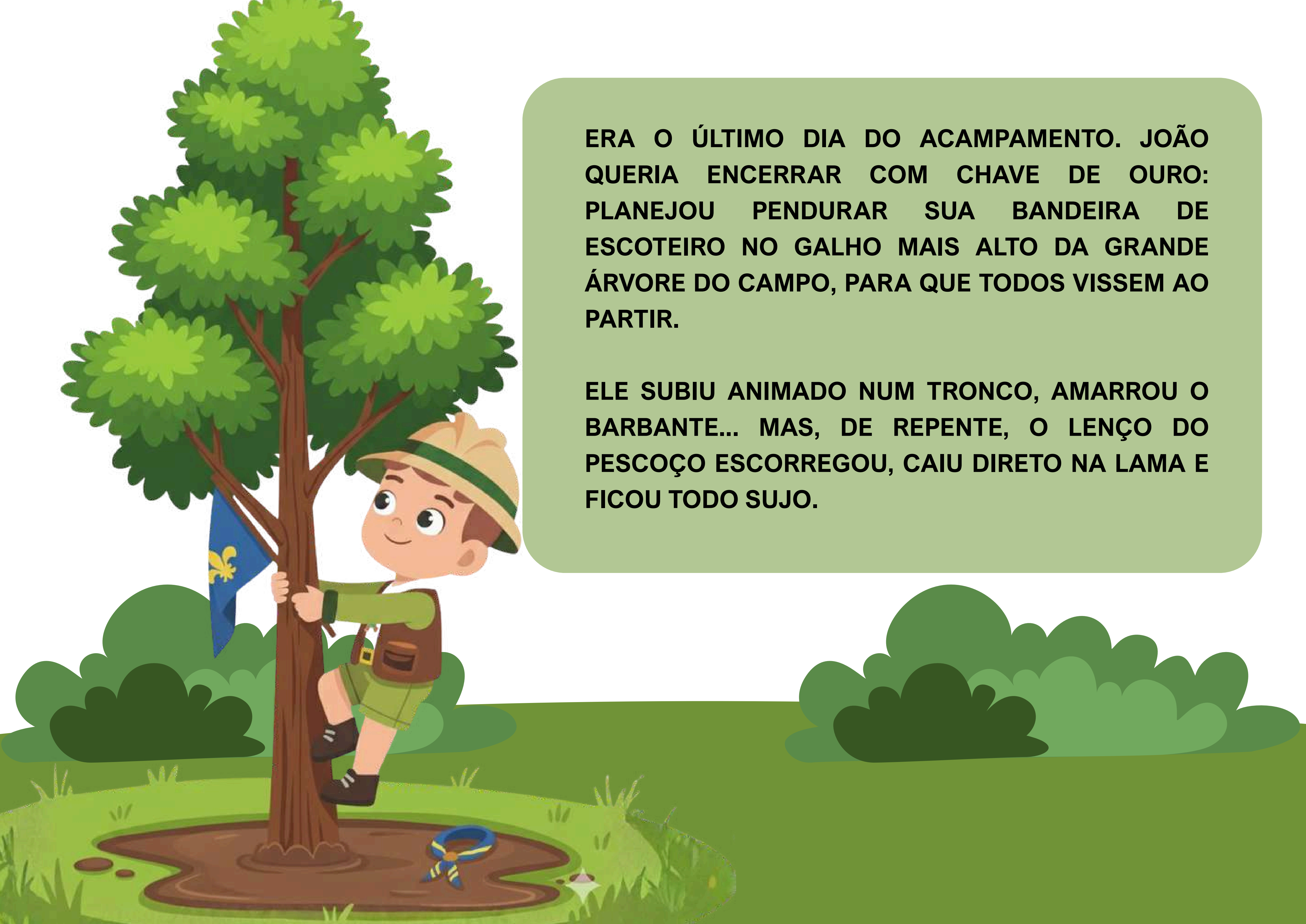

ERA O ÚLTIMO DIA DO ACAMPAMENTO. JOÃO QUERIA ENCERRAR COM CHAVE DE OURO: PLANEJOU PENDURAR SUA BANDEIRA DE ESCOTEIRO NO GALHO MAIS ALTO DA GRANDE ÁRVORE DO CAMPO, PARA QUE TODOS VISSEM AO PARTIR.

ELE SUBIU ANIMADO NUM TRONCO, AMARROU O BARBANTE... MAS, DE REPENTE, O LENÇO DO PESCOÇO ESCORREGOU, CAIU DIRETO NA LAMA E FICOU TODO SUJO.

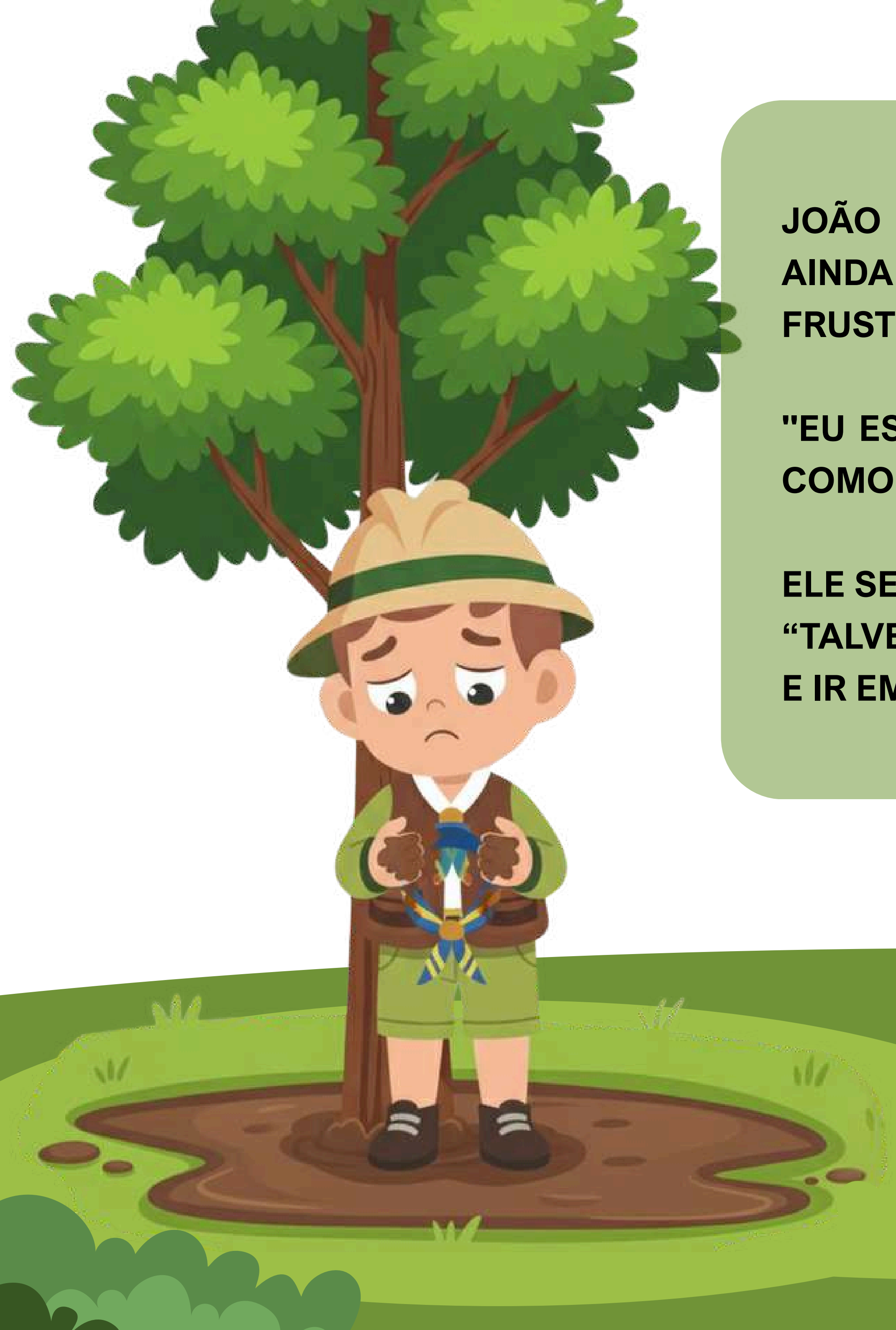

JOÃO TENTOU LIMPAR NA GRAMA, MAS ESPALHOU AINDA MAIS A SUJEIRA. SENTIU UM NÓ DE FRUSTRAÇÃO NO PEITO, OS OLHOS ARDERAM.

"EU ESTRAGUEI MEU ÚLTIMO DIA... NADA NUNCA SAI COMO EU QUERO."

ELE SEGUROU O CHORO, PENSOU EM DESISTIR:
“TALVEZ SEJA MELHOR GUARDAR TUDO NA MOCHILA E IR EMBORA LOGO.”

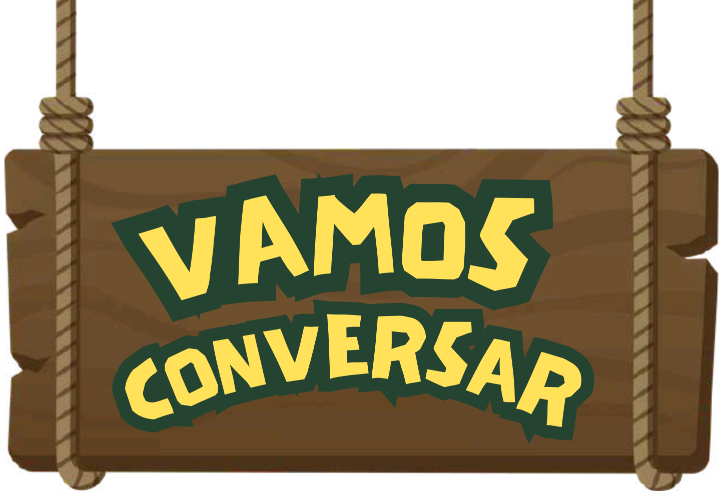

1. Qual é o personagem da história?
2. Qual é a situação inicial?
3. Qual é o problema da história?
4. Como o personagem se sentiu?

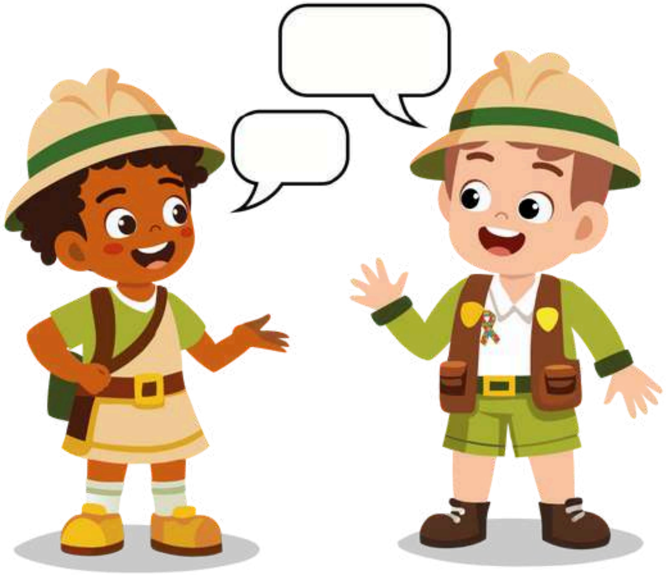

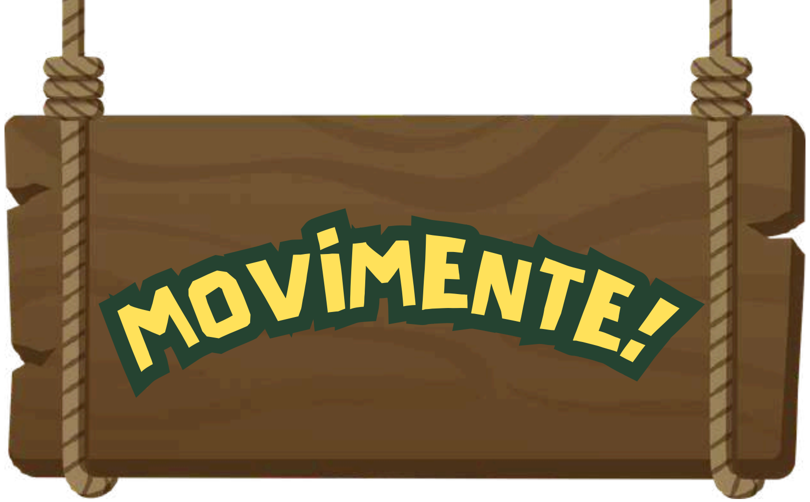

1. João estava muito desmotivado. Para tentar se animar, ele foi brincar, e usando uma rede no bambolê, ele fingiu pegar borboletas, enquanto o líder da tropa jogava bolinhas para ele.

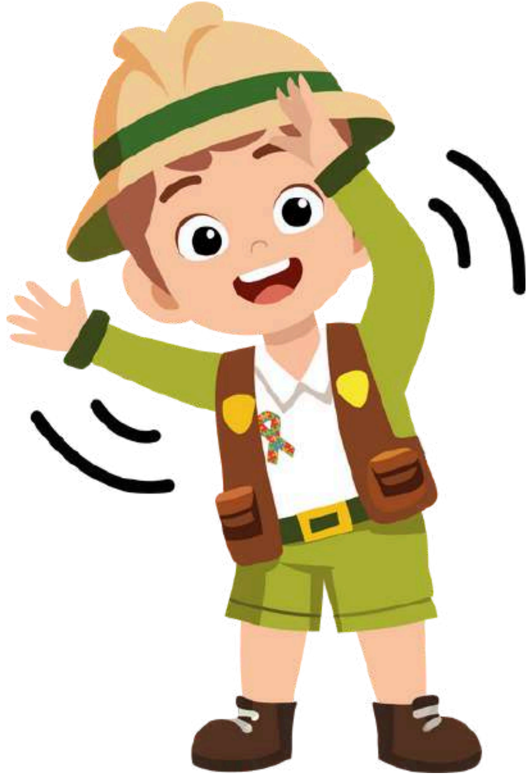

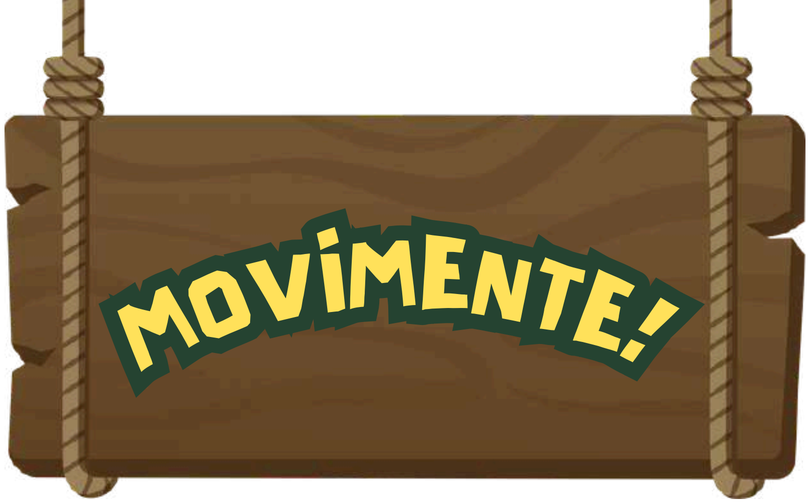

2. Depois, ele tentou outra atividade, que era deitar com os joelhos esticados encostando somente a ponta dos pés cotovelos e parte dos braços no chão. Exigia muita concentração e fazia ele esquecer dos problemas.

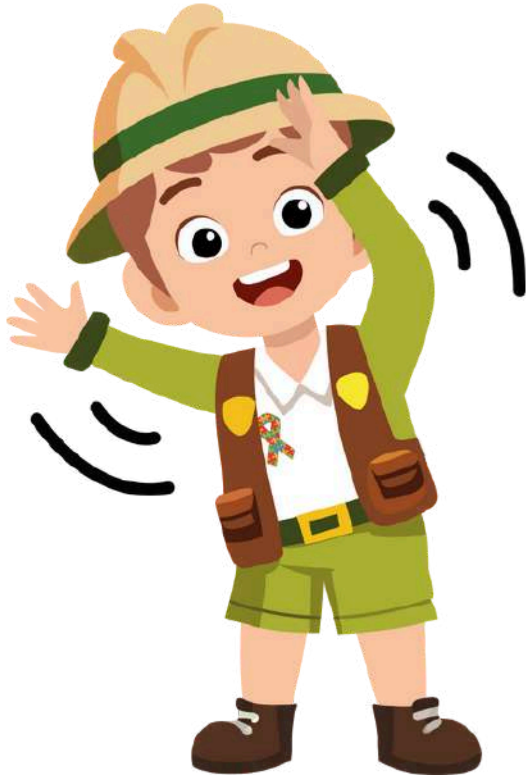

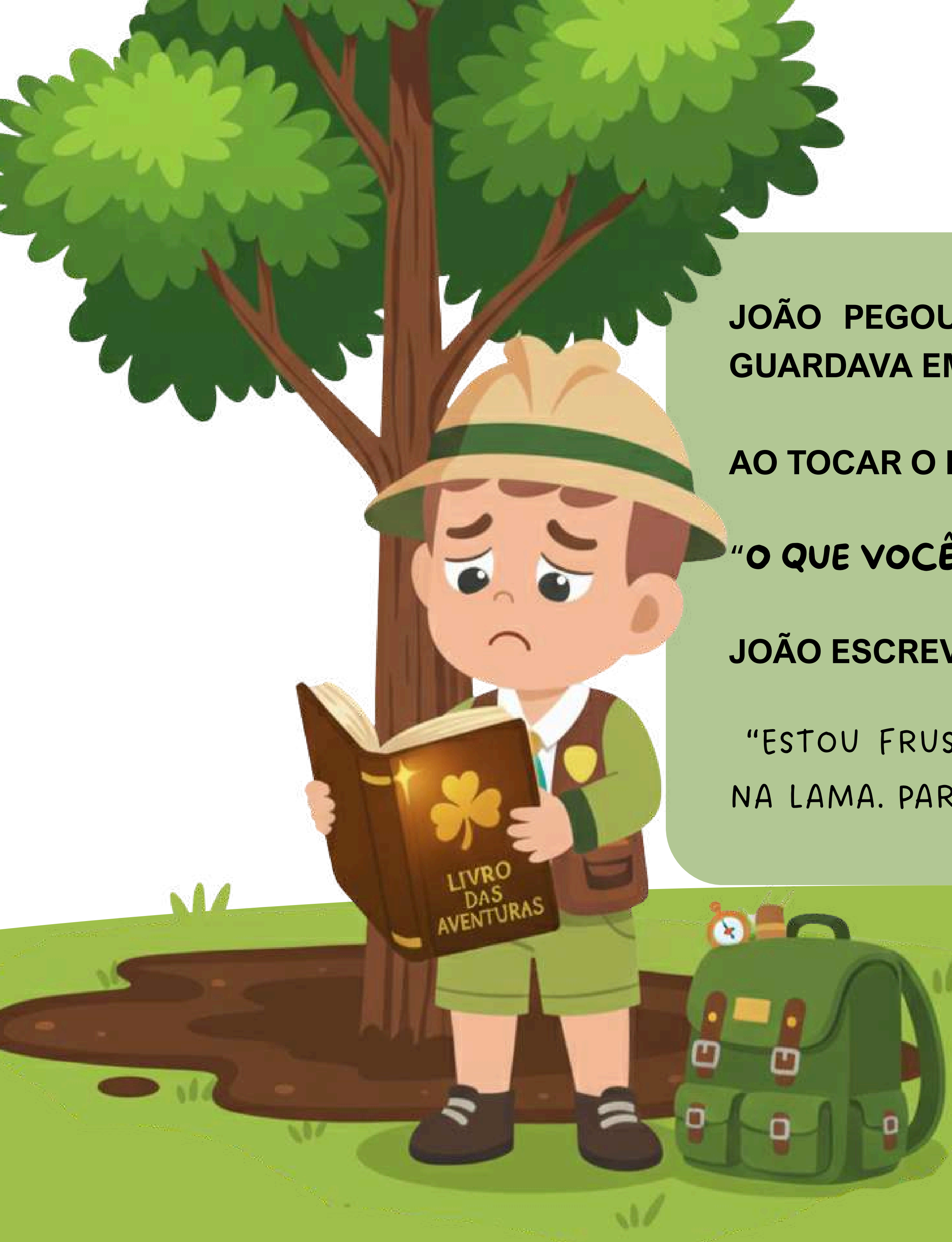

JOÃO PEGOU O SEU LIVRO DE AVENTURAS, QUE ELE GUARDAVA EM SUA MOCHILA DE ESCOTEIRO.

AO TOCAR O LIVRO, AS LETRAS DOURADAS SURGIRAM:

“O QUE VOCÊ ESTÁ SENTINDO?

JOÃO ESCREVEU:

“ESTOU FRUSTRADO POR TER DERRUBADO MEU LENÇO NA LAMA. PARECE QUE NADA DÁ CERTO PARA MIM!”

TRÊS CAMINHOS SURGIRAM DIANTE DELE:

1. GUARDAR O LENÇO NA MOCHILA E DESISTIR DA BANDEIRA.

2. PENDURAR A BANDEIRA MESMO COM O LENÇO TODO SUJO.

3. LAVAR O LENÇO NO POÇO, ESPERAR SECAR, E PENDURAR A BANDEIRA COM CUIDADO.

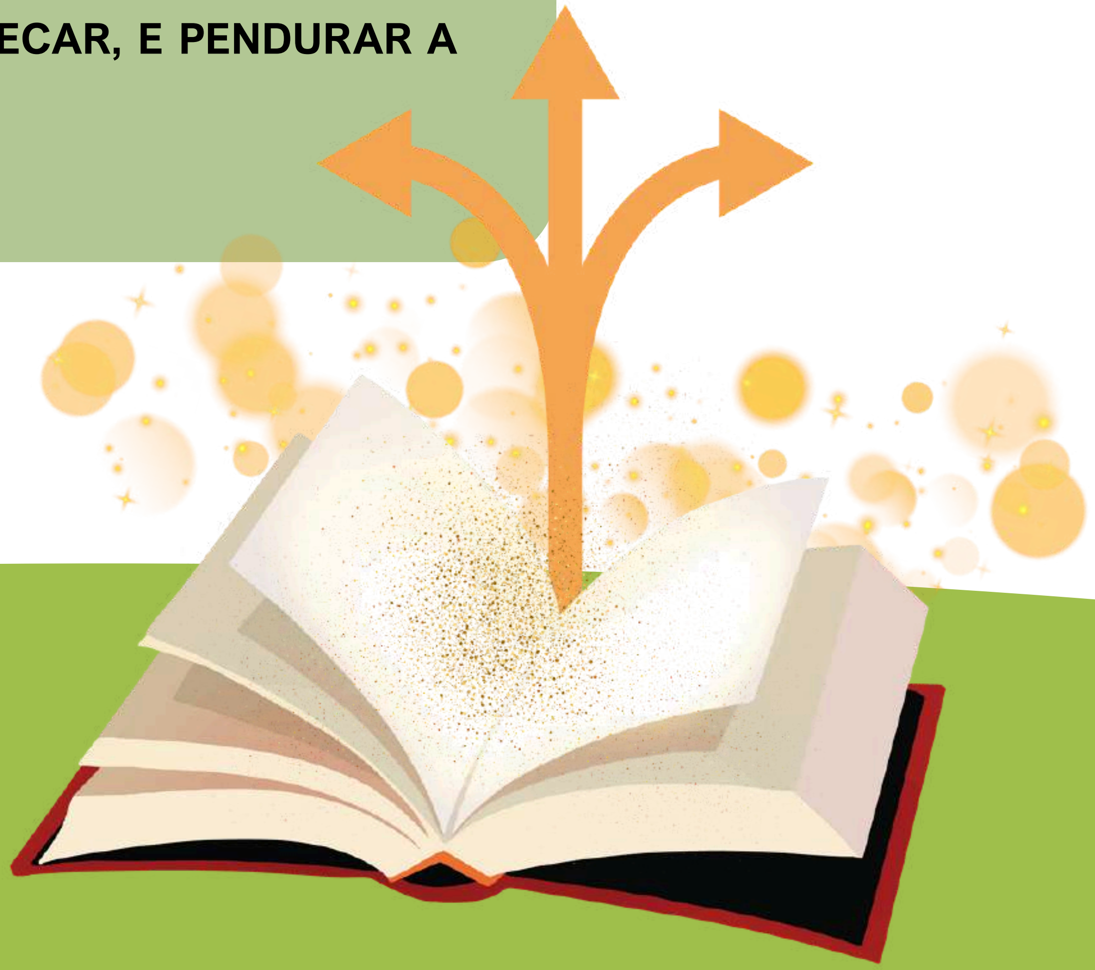

VAMOS CONVERSAR

1. O que você acha que vai acontecer se João seguir cada um desses caminhos?

2. Qual é a melhor opção?

3. Quais podem ser os planos de João para resolver o problema?

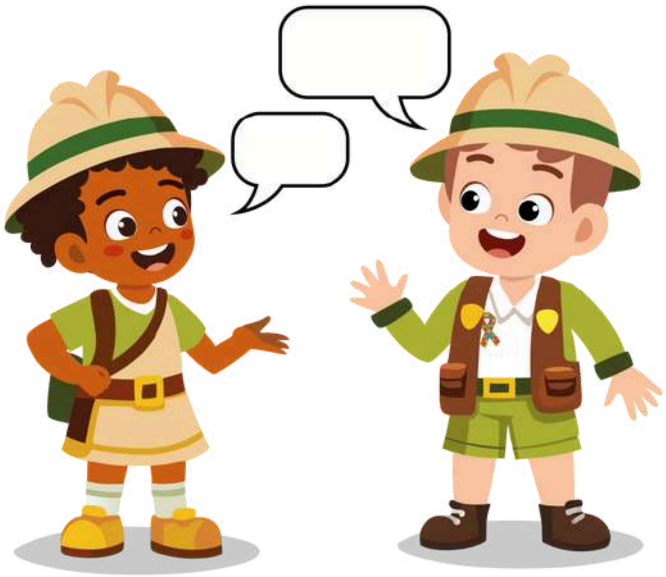

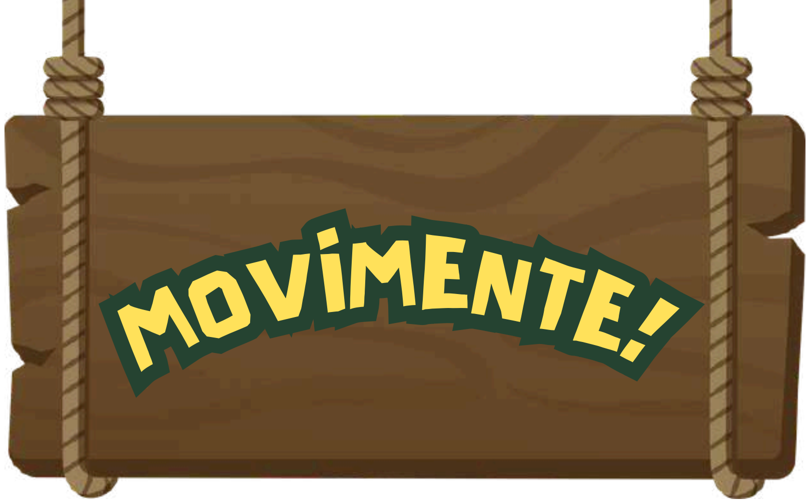

1. João guardou o lenço na mochila e quis desistir da bandeira. Começou a correr sem sair do lugar e com alguns comandos do lider, parava com um pé só.

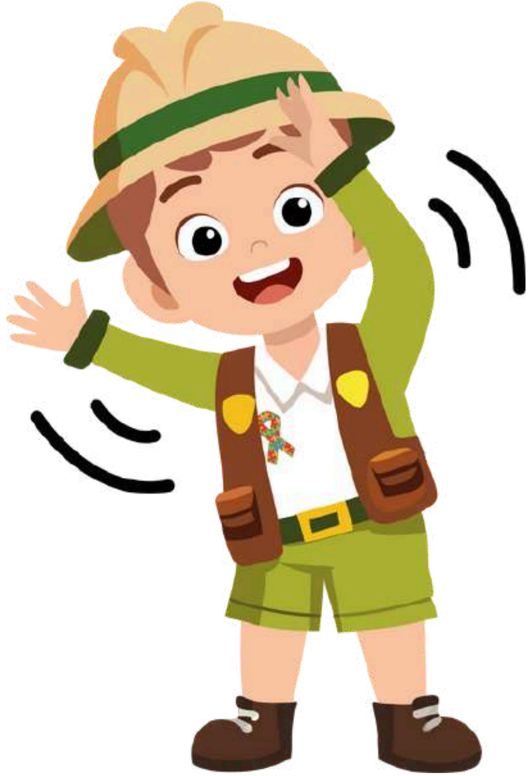

MOVIMENTE!

2. João pensou em pendurar a bandeira mesmo com o lenço todo sujo. Ele tentou saltar em pequenas "poças" de água, sem colocar o outro pé no chão até o final do caminho.

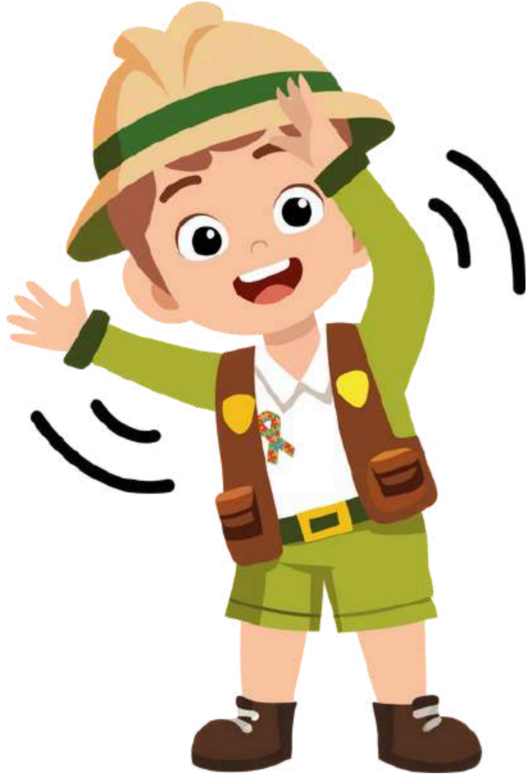

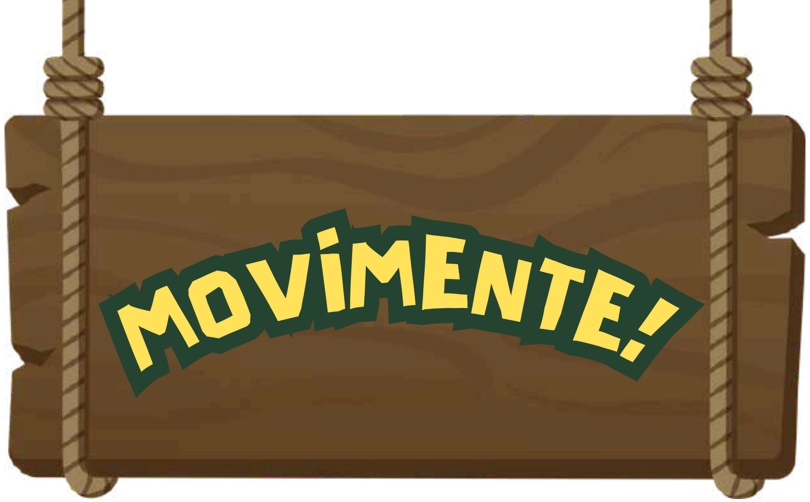

3. João, depois, lavou o lenço no poço,esperou secar, e pendurou a bandeira com cuidado. Para chegar no lugar, precisou andar como se tivesse fazendo o movimentodo *Avanço* deslocando para frente.

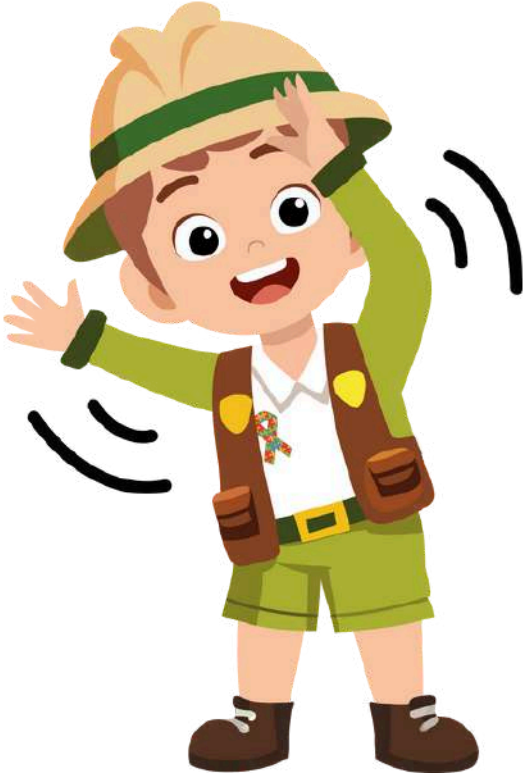

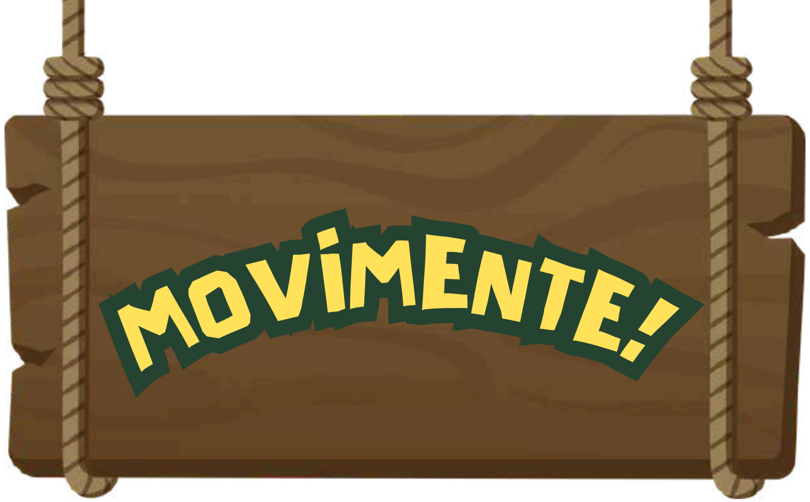

4. Para alcançar o espaço com ajuda do líder da tropa, João andou de carrinho de mão, até o local onde deixaria a bandeira.

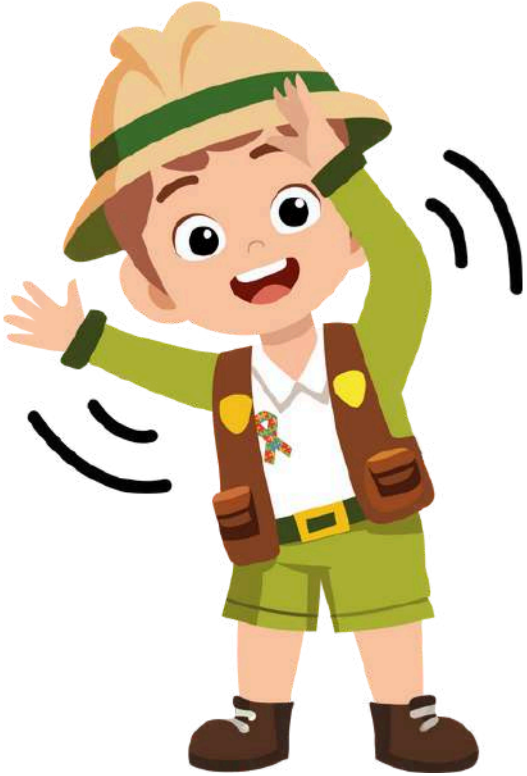

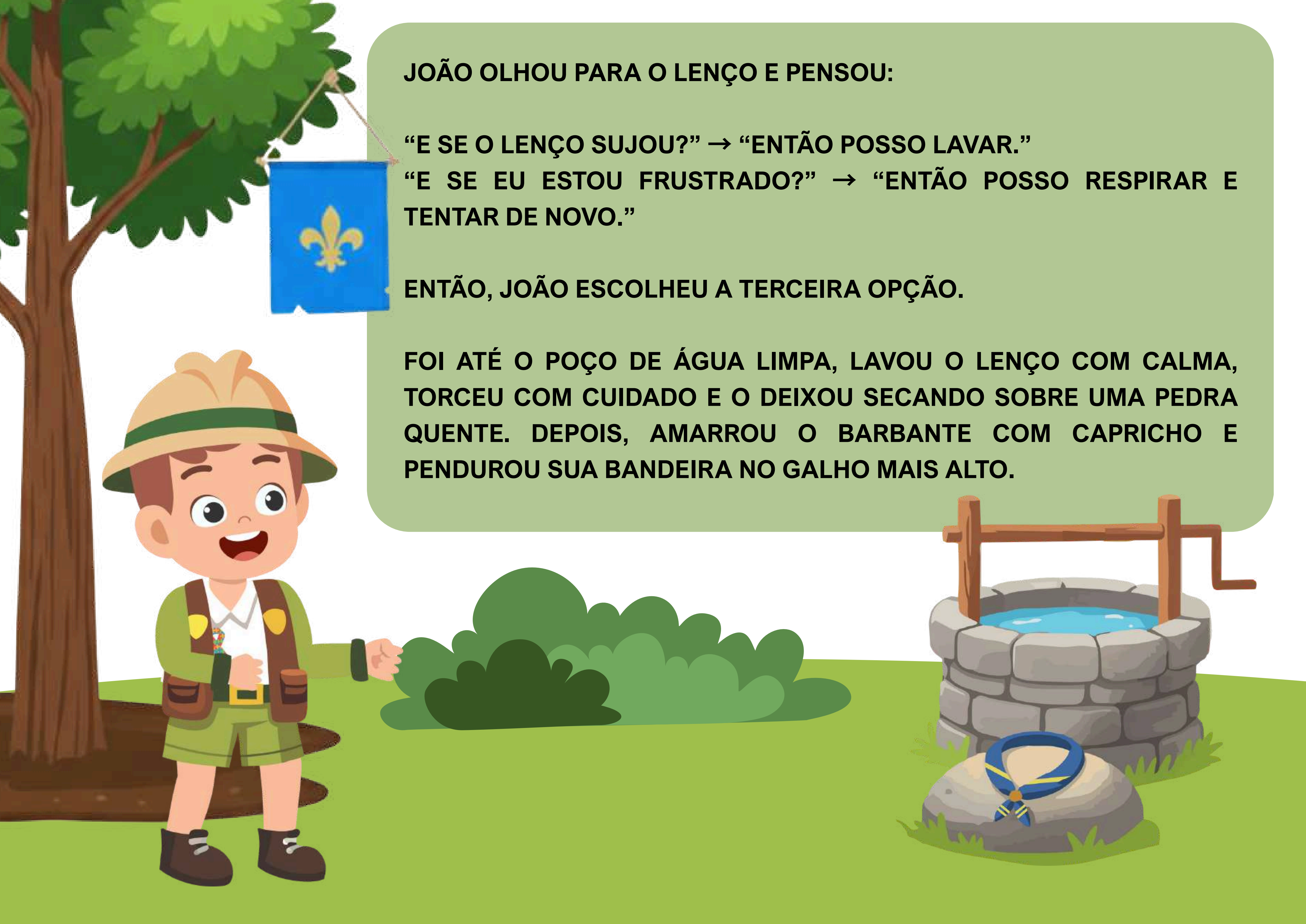

JOÃO OLHOU PARA O LENÇO E PENSOU:

“E SE O LENÇO SUJOU?” → “ENTÃO POSSO LAVAR.”
“E SE EU ESTOU FRUSTRADO?” → “ENTÃO POSSO RESPIRAR E TENTAR DE NOVO.”

ENTÃO, JOÃO ESCOLHEU A TERCEIRA OPÇÃO.

FOI ATÉ O POÇO DE ÁGUA LIMPA, LAVOU O LENÇO COM CALMA, TORCEU COM CUIDADO E O DEIXOU SECANDO SOBRE UMA PEDRA QUENTE. DEPOIS, AMARROU O BARBANTE COM CAPRICHO E PENDUROU SUA BANDEIRA NO GALHO MAIS ALTO.

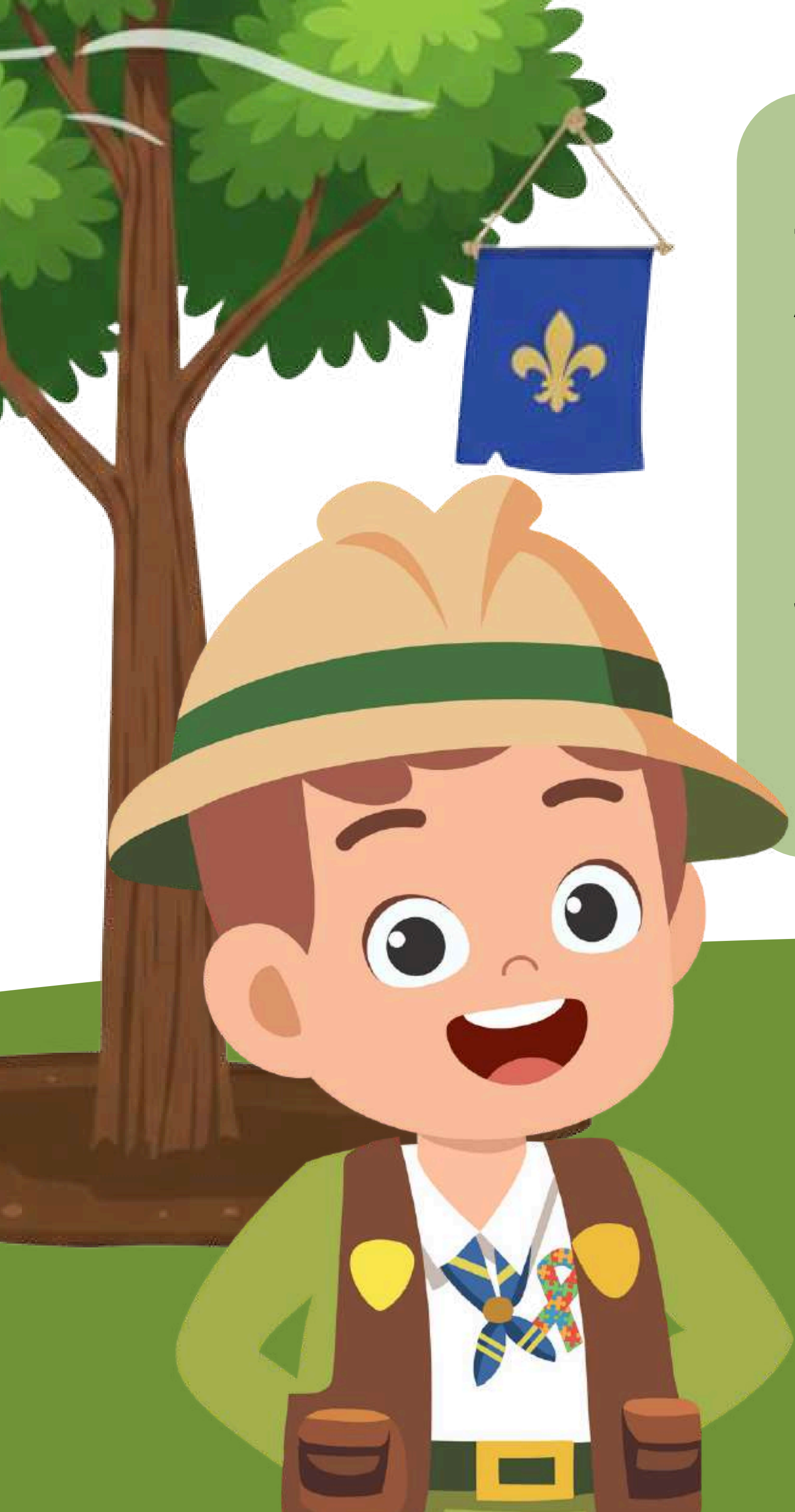

QUANDO TUDO FICOU PRONTO, JOÃO DEU UM PASSO PARA TRÁS E OBSERVOU SUA BANDEIRA TREMULANDO LÁ NO ALTO, COM O LENÇO AGORA LIMPO, AMARRADO COM ORGULHO.

O VENTO SOPROU LEVE, COMO UM AGRADECIMENTO SILENCIOSO.
JOÃO SORRIU.

"EU NÃO DEIXEI A FRUSTRAÇÃO ESTRAGAR MEU ÚLTIMO DIA. EU POSSO TENTAR DE NOVO, SEMPRE."

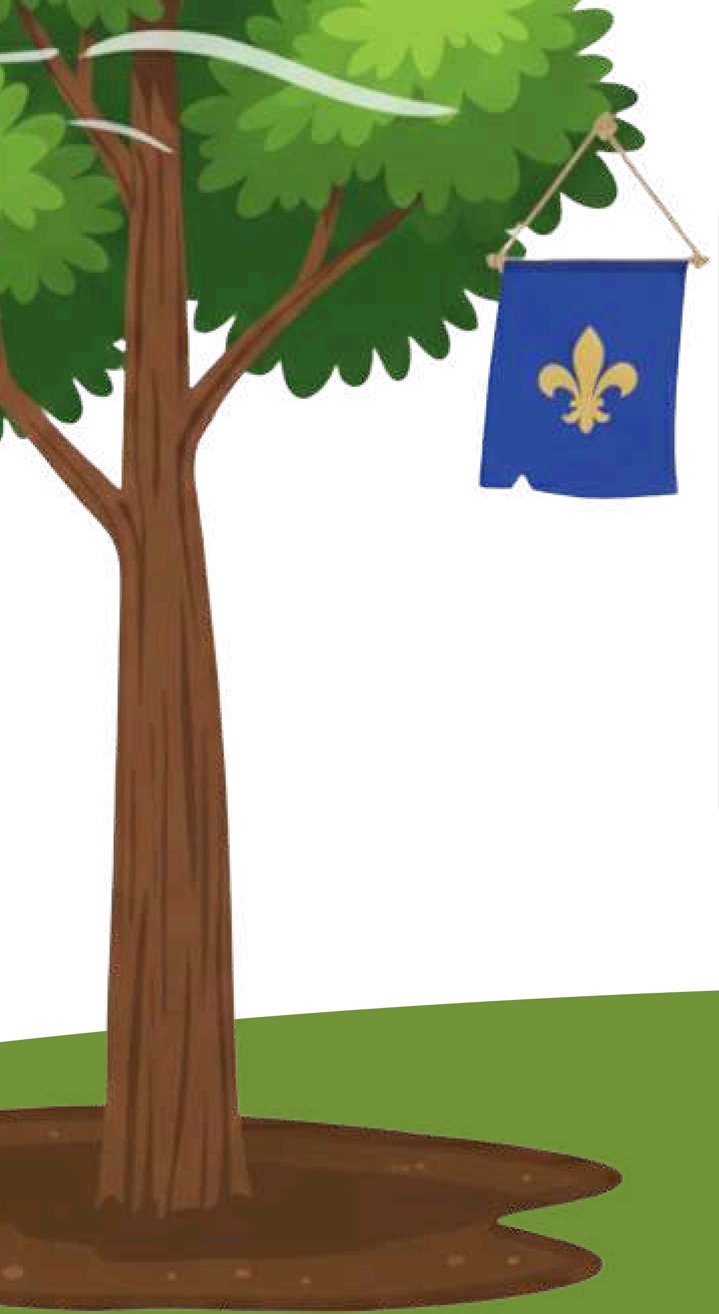

JOÃO ESCREVEU NO LIVRO DE AVENTURAS:

"HOJE FOI O ÚLTIMO DIA DO ACAMPAMENTO. TEVE SOL, LAMA, E UM LENÇO SUJO BEM NA HORA ERRADA. MAS EU LAVEI, ESPEREI, E PENDUREI A BANDEIRA MESMO ASSIM. NEM TUDO SAI PERFEITO. E TUDO BEM. PORQUE EU POSSO ME FRUSTRAR E MESMO ASSIM TERMINAR COM ORGULHO."

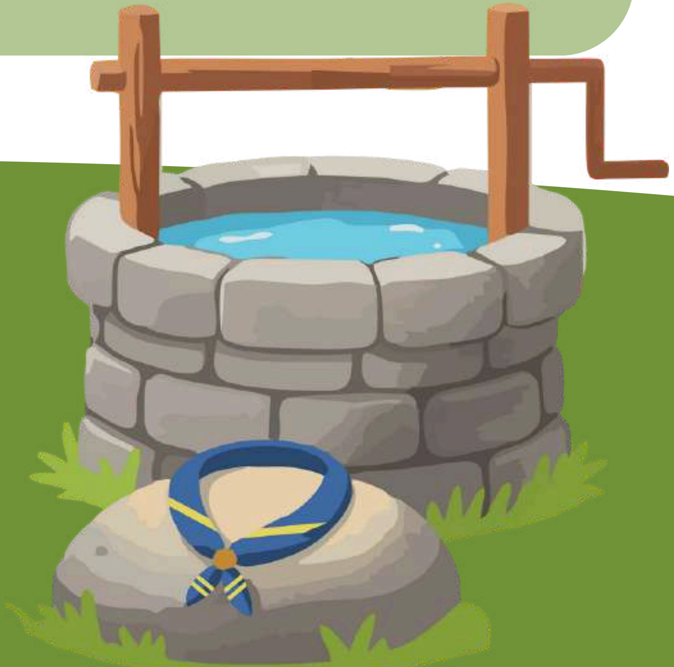

VAMOS CONVERSAR

1. Qual foi a ação de João?
2. Qual é a situação final da história?
3. Reconte a história com as suas palavras.

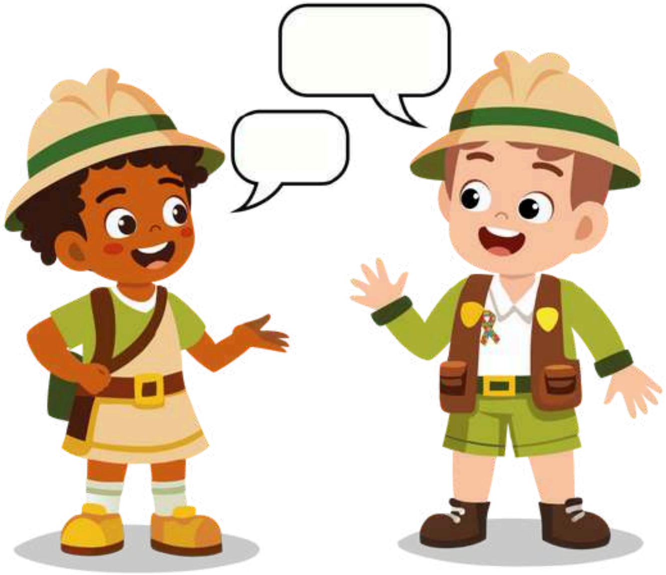

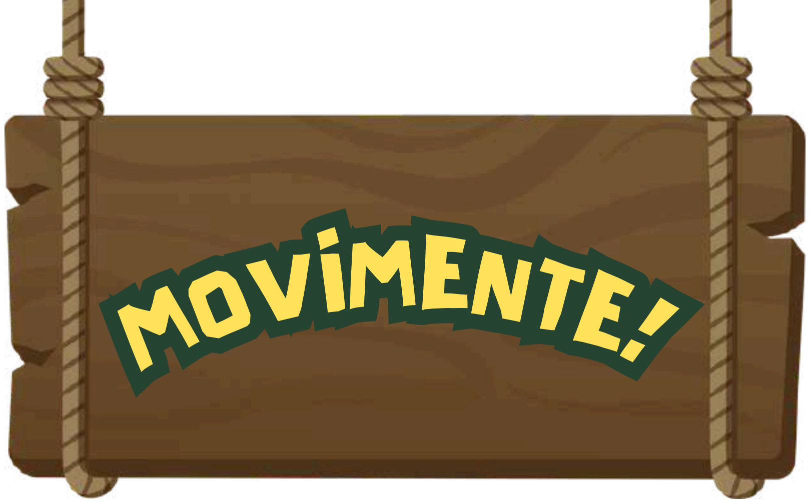

1. Durante o trajeto, para se divertir, ele e o líder fizeram troca de passes com os pés.

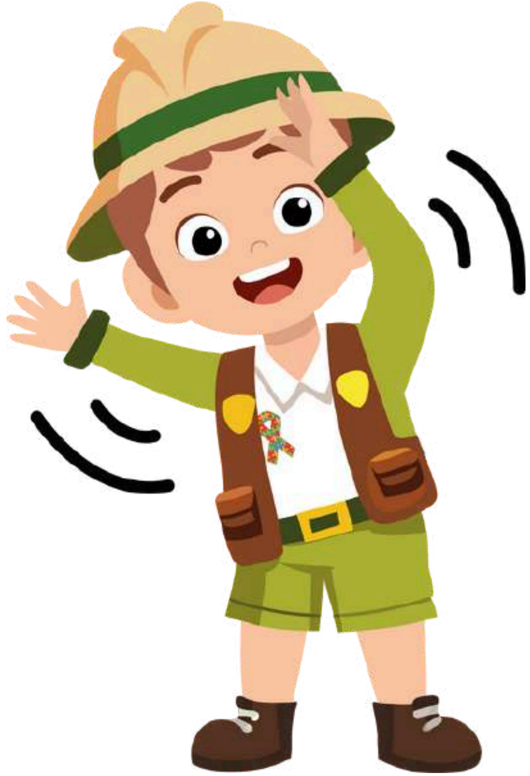

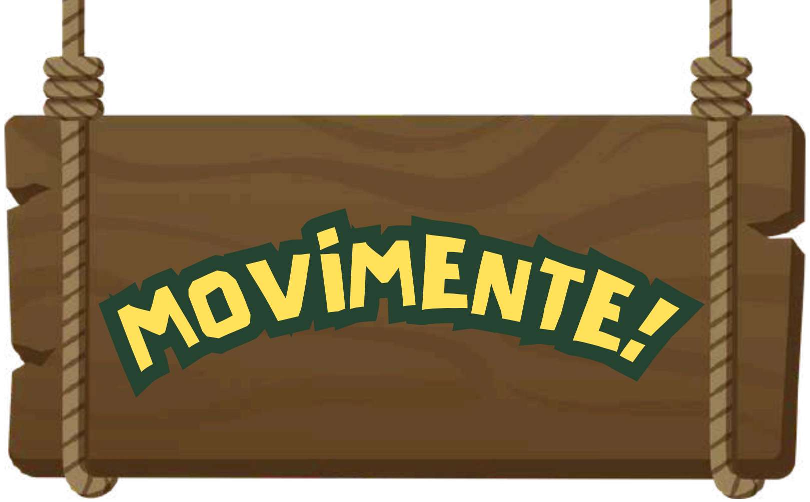

2. Em uma parte do trajeto, eles usaram a corda. Eles pularam com a corda para atravessar um caminho mais sombrio e não ter medo.

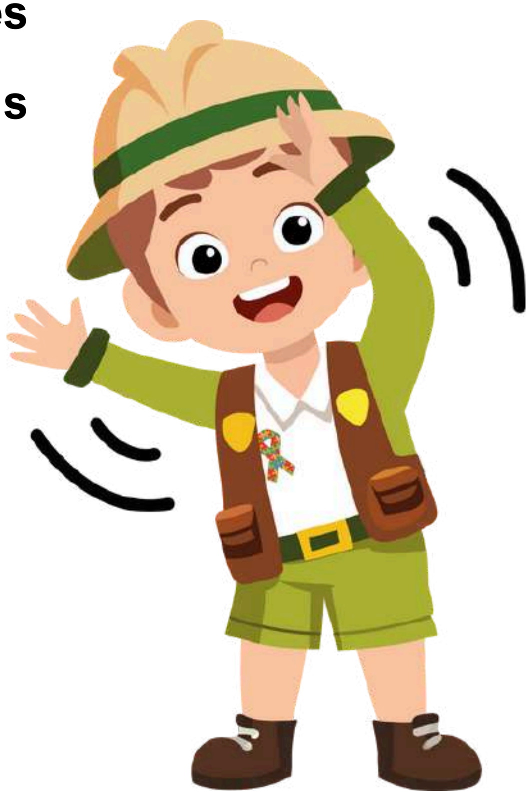

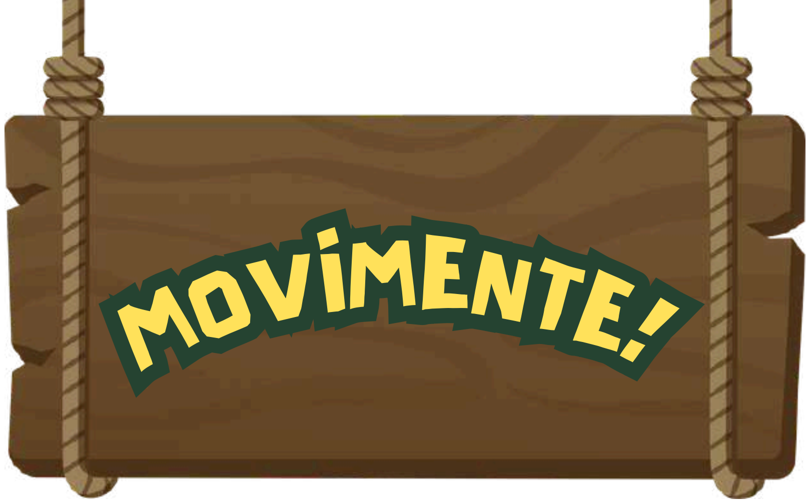

3. No final do trajeto, havia uma parede de pedra atrapalhando o caminho. Para conseguir passar, eles usaram uma bola, chutando em pontos específicos da parede até ela cair (chute a gol).

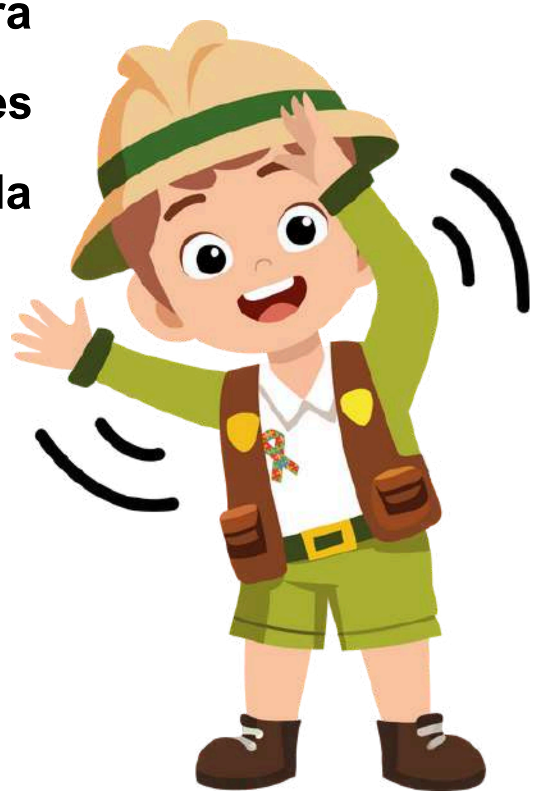

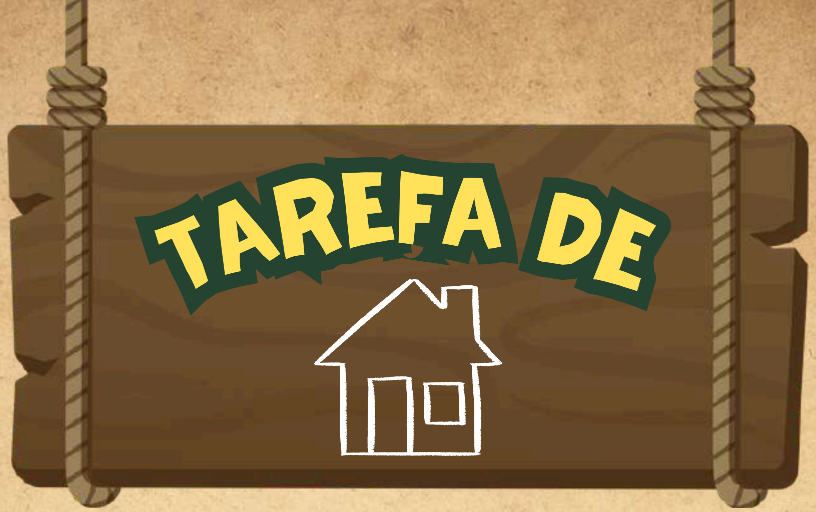

NOSSO ÚLTIMO DIA DE JORNADA🌟

ASSIM COMO JOÃO, QUE ENCONTROU FORÇAS PARA LAVAR O LENÇO E PENDURAR SUA BANDEIRA NO ALTO DA ÁRVORE, VOCÊ TAMBÉM COMPLETOU SUA AVENTURA. DURANTE ESSAS 15 SESSÕES, APRENDEU A RECONHECER AS CINCO EMOÇÕES PRINCIPAIS E DESCOBRIU CAMINHOS PARA LIDAR COM O MEDO, A RAIVA E A FRUSTRAÇÃO.

VOCÊ RESPIROU FUNDO, PENSOU EM NOVAS ESTRATÉGIAS, PEDIU AJUDA QUANDO PRECISOU E MOSTROU QUE SEMPRE É POSSÍVEL TENTAR DE NOVO. NEM TUDO SAI PERFEITO, E TUDO BEM. O IMPORTANTE É LEMBRAR QUE CADA PASSO, ATÉ OSMAIS DIFÍCEIS, FAZ PARTE DO APRENDIZADO.AGORA, NO SEU LIVRO DE AVENTURAS, ESCREVA UMA NOVA PÁGINA CHAMADA:

✨ “MEU PLANO PARA QUANDO EU SENTIR MEDO DE ERRAR” ✨

ALI, DESENHE OU ESCREVA DUAS COISAS QUE VÃO AJUDÁ-LO QUANDO O MEDO APARECER, COMO RESPIRAR FUNDO, OLHAR DE NOVO, PEDIR AJUDA OU IR MAIS DEVAGAR.
